# Molecular diversity of the base-promoted reaction of phenacylmalononitriles with dialkyl but-2-ynedioates

**DOI:** 10.3762/bjoc.18.99

**Published:** 2022-08-08

**Authors:** Hui Zheng, Ying Han, Jing Sun, Chao-Guo Yan

**Affiliations:** 1 College of the Chemistry & Chemical Engineering, Yangzhou University, China

**Keywords:** carboxamide, cycloaddition, cyclopentene, electron-deficient alkyne, phenacylmalononitrile

## Abstract

In the presence of tetrabutylammonium bromide (TBAB), the cycloaddition reaction of phenacylmalononitriles with dialkyl but-2-ynedioates in acetonitrile at room temperature resulted in 3,3-dicyano-5-hydroxy-5-arylcyclopent-1-ene-1,2-dicarboxylates in high yields. More importantly, the DABCO-promoted domino reaction of two molecules of each phenacylmalononitrile and dialkyl but-2-ynedioate in acetonitrile at room temperature afforded unique multifunctionalized carboxamide-bridged dicyclopentenes in moderate to good yields and with high diastereoselectivity.

## Introduction

**Scheme 1:**
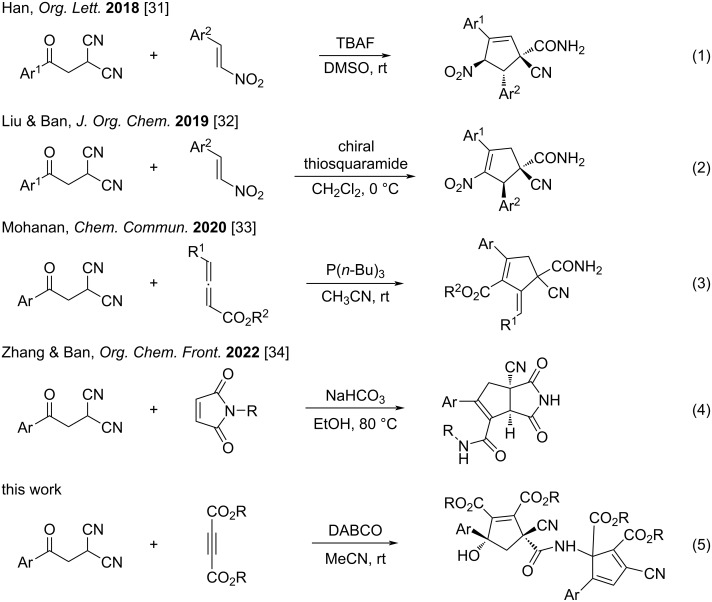
Representative cycloaddition reactions of phenacylmalononitriles.

Phenacylmalononitrile is one of the privileged functionalized compounds [1–5], because it contains one carbonyl group, two cyano groups, and an activated methylene unit. Phenacylmalononitrile exhibits versatile reactivity, high synthetic efficiency and molecular diversity and has been widely employed in many synthetic reactions [6–10]. On the other hand, phenacylmalononitrile is also a readily available substrate, which can be easily prepared through a base-promoted substitution reaction of phenacyl bromide with malononitrile under mild conditions [11–16]. In many practical cases, phenacylmalononitriles could be conveniently generated in situ by directly using a mixture of phenacyl bromide and malononitrile in the reaction system [17–22]. As a consequence, the unique features of phenacylmalononitriles make them good candidates for the efficient construction of diverse carbocyclic and heterocyclic compounds [23–30]. For example, Han and co-workers successfully developed a tetrabutylammonium fluoride-catalyzed cycloaddition of phenacylmalononitriles and nitroolefins for the diastereoselective synthesis of multifunctionalized cyclopent-2-ene-1-carboxamides [31] (reaction 1 in Scheme 1). Liu and Ban furnished a chiral thiosquaramide-catalyzed tandem Michael–Henry reaction of phenacylmalononitriles and nitroolefins for the enantioselective synthesis of cyclopent-3-ene-1-carboxamides [32] (reaction 2 in Scheme 1). Mohanan and co-workers reported a PBu_3_-catalyzed [3 + 2] annulation of phenacylmalononitriles and allenoates for the tunable synthesis of multifunctionalized cyclopentene carboxamides and cyclopentenols [33] (reaction 3 in Scheme 1). Recently, Zhang and Ban reported a NaHCO_3_-promoted reaction of phenacylmalononitriles and *N*-alkylmaleimides to give the novel cyclopenta[*c*]pyrrole-4-carboxamides with unprecedented rearrangement of the alkyl group [34] (reaction 4 in Scheme 1). Inspired by these novel reactions and in continuation of our aim to develop domino reactions of electron-deficient alkynes [35–50], we have investigated the base-promoted reactions between phenacylmalononitriles and dialkyl but-2-ynedioates. Here we wish to report the selective synthesis of cyclopent-1-ene-1,2-dicarboxylates and complex carboxamide-bridged dicyclopentene derivatives in good yields and with high diastereoselectivity (reaction 5 in Scheme 1).

## Results and Discussion

Initially, the reaction conditions were briefly optimized by using *p*-methylphenacylmalononitrile (**1a**) and diethyl but-2-ynedioate (**2a**) as standard according to the previous reported work [31]. In the presence of potassium carbonate and tetrabutylammonium chloride (TBAC), the reaction in acetonitrile at room temperature gave diethyl 3,3-dicyano-5-hydroxy-5-(*p*-methylphenyl)cyclopent-1-ene-1,2-dicarboxylate (**3a**) in moderate yield (Table 1). In the presence of tetrabutylammonium bromide (TBAB), the reaction in acetonitrile afforded product **3a** in 85% yield. The reaction in DCM gave the product **3a** in 65% yield. However, no product **3a** was obtained when the reaction was carried out in toluene at room temperature or in acetonitrile at 0 °C. The yield of **3a** remained the same when the reaction was carried out at elevated temperature. At last, using of higher loading of TBAB and prolonging the reaction time could not increase the yield of the product **3a**. Therefore, the functionalized 5-hydroxy-cyclopent-1-ene derivatives can be conveniently prepared in satisfactory yield in very simple reaction conditions. It should be pointed that a similar triethylamine-promoted multicomponent reaction of phenacyl bromide, malononitrile, dialkyl but-2-ynedioate, and triphenylphosphine has been already reported, in which diethyl 3-phenyl-5,5-dicyanocyclopent-2-ene-1,2-dicarboxylates were produced by further elimination of a hydroxy group [30]. In the present reaction, the hydroxy group is still remained in the product **3a**. This result might be due to the weak basic system and the milder conditions. An attempt to develop a three-component reaction by directly using the phenacyl bromide and malononitrile to replace the previously prepared phenacylmalononitrile in the reaction was not successful. The reaction gave a very complex mixture of products.

**Table 1:** Optimization of reaction conditions.^a^

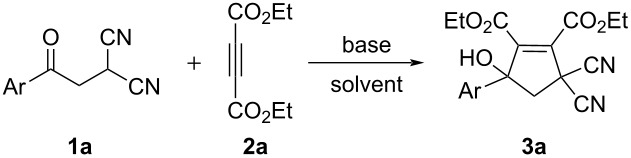					

Entry	Base	Solvent	Temp. (°C)	Time (h)	Yield (%)^b^

1	K_2_CO_3_	MeCN	rt	12	50
2	TBAC	MeCN	rt	12	35
3	**TBAB**	**MeCN**	**rt**	**12**	**85**
4	TBAB	DCM	rt	12	65
5	TBAB	PhMe	rt	12	–
6	TBAB	MeCN	0	12	–
7	TBAB	MeCN	50	12	75
8	TBAB^c^	MeCN	rt	12	84
9	TBAB	MeCN	rt	24	84

^a^Reaction conditions: *p*-methylphenacylmalononitrile (0.5 mmol), dialkyl but-2-ynedioate (0.6 mmol), base (0.25 mmol), solvent (5.0 mL). ^b^Isolated yields. ^c^TBAB (0.5 mmol) was used.

**Figure 1:**
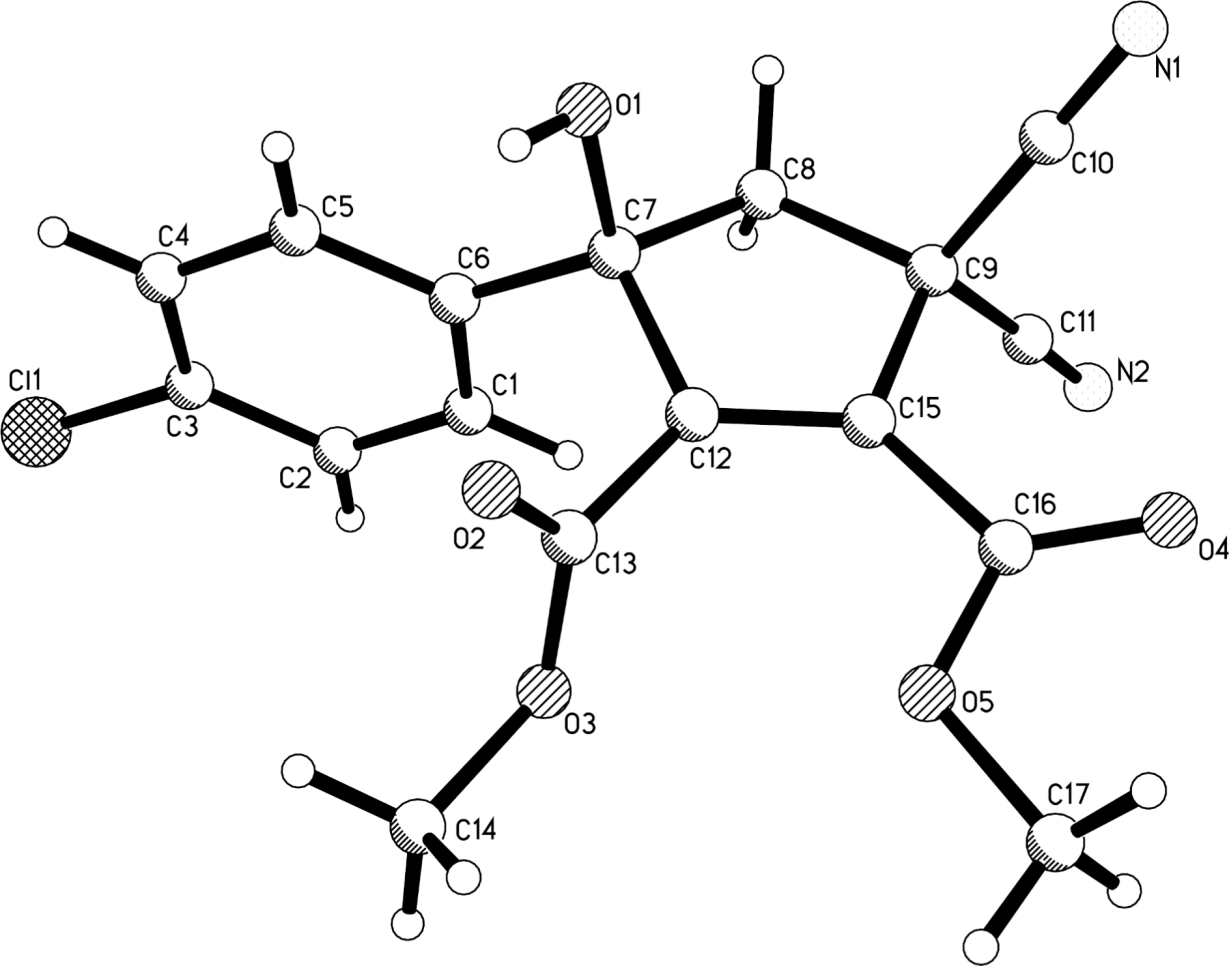
Single crystal structure of compound **3k**.

Under the optimized reaction conditions (Table 1, entry 3), the scope of the reaction was investigated by using various substrates and the results are summarized in Table 2. It can be seen that all reactions proceeded smoothly to give the expected functionalized 5-hydroxycyclopentenes **3a–l** in good to excellent yields. The phenacylmalononitriles with electron-donating groups usually gave higher yields than that of substrates bearing an electron-donating chloro, bromo and nitro group. Both diethyl and dimethyl but-2-ynedioates can be successfully employed in the reaction. Because there is only one chiral carbon atom in the molecule, there are no diastereoisomers in the obtained products **3a**–**l**. The chemical structures of compounds **3a**–**l** were fully characterized by IR, HRMS, ^1^H and ^13^C NMR spectra. As for an example, the ^1^H NMR spectrum of compound **3i** displayed a singlet at 3.52 ppm for the hydroxy group and two singlets at 3.24, 3.96 ppm with *J* = 14.8 Hz for the two diastereotopic protons of the cyclic methylene unit. The single crystal structure of compound **3k** was successfully determined by X-ray diffraction analysis (Figure 1). From Figure 1, it can be seen that the C–C double bond is connected to two methoxycarbonyl groups. Though one hydroxy group exists on the reactive allyl position and benzyl position, it still is present in the molecule and did not give the cyclopentadiene by further elimination of water.

**Table 2:** Synthesis of functionalized cyclopentenes **3a**–**l**.^a^

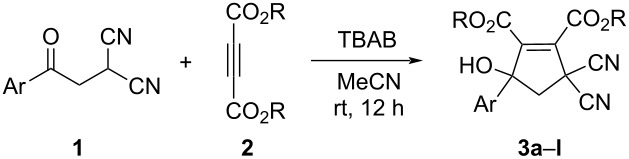				

Entry	Product	Ar	R	Yield (%)^b^

1	**3a**	*p*-CH_3_C_6_H_4_	Et	85
2	**3b**	C_6_H_5_	Et	75
3	**3c**	*m*-CH_3_OC_6_H_4_	Et	73
4	**3d**	*p-*CH_3_OC_6_H_4_	Et	88
5	**3e**	*o*-CH_3_OC_6_H_4_	Et	62
6	**3f**	*p*-ClC_6_H_4_	Et	60
7	**3g**	*p-*BrC_6_H_4_	Et	62
8	**3h**	*p*-NO_2_C_6_H_4_	Et	56
9	**3i**	*p-*CH_3_C_6_H_4_	Me	78
10	**3j**	*p-*CH_3_OC_6_H_4_	Me	80
11	**3k**	*p*-ClC_6_H_4_	Me	52
12	**3l**	*p-*BrC_6_H_4_	Me	55

^a^Reaction conditions: phenacylmalononitrile (0.5 mmol), dialkyl but-2-ynedioate (0.6 mmol), TBAB (0.25 mmol), CH_3_CN (5.0 mL). rt, 12 h. ^b^Isolated yields.

**Figure 2:**
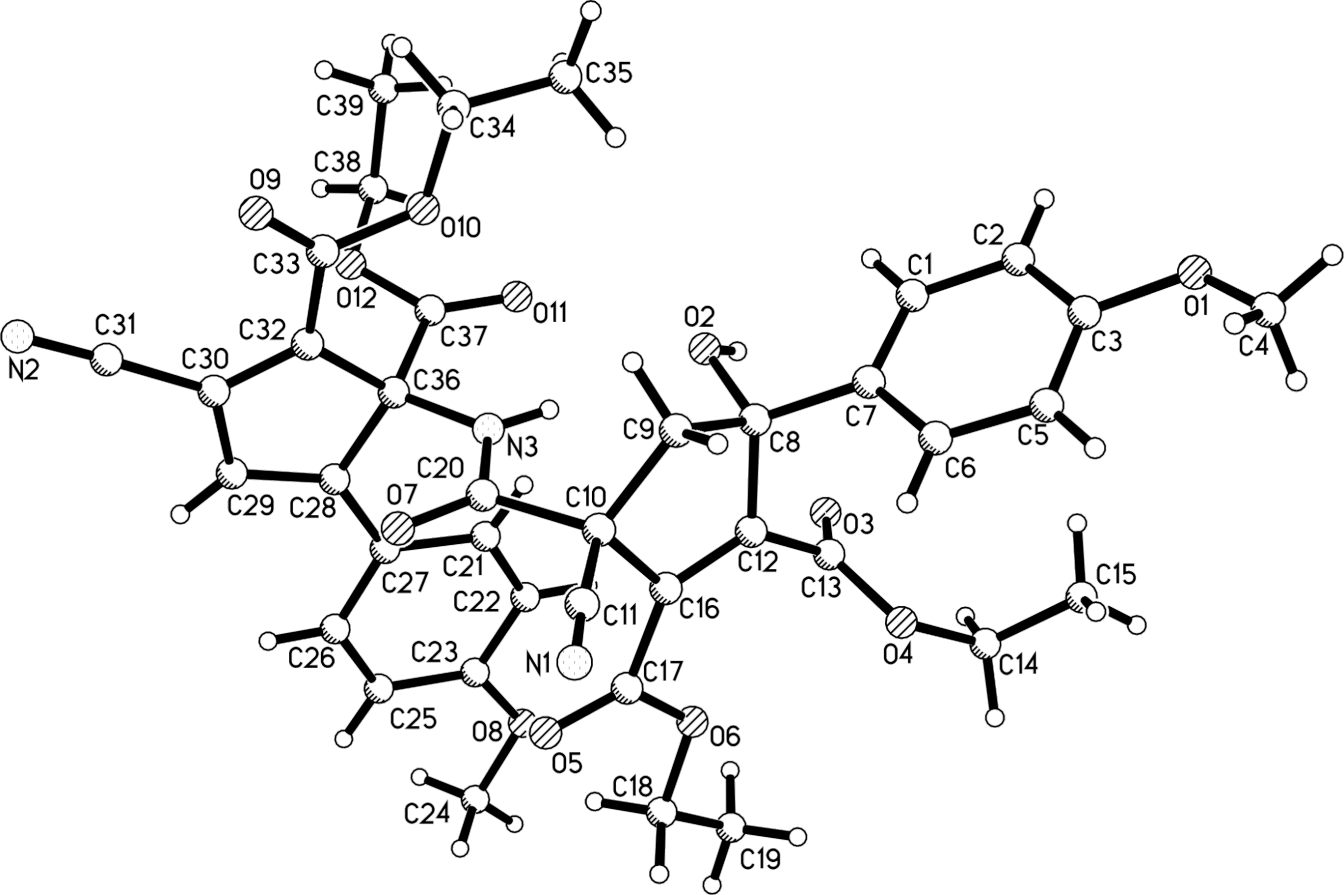
Single crystal structure of compound **4a**.

**Figure 3:**
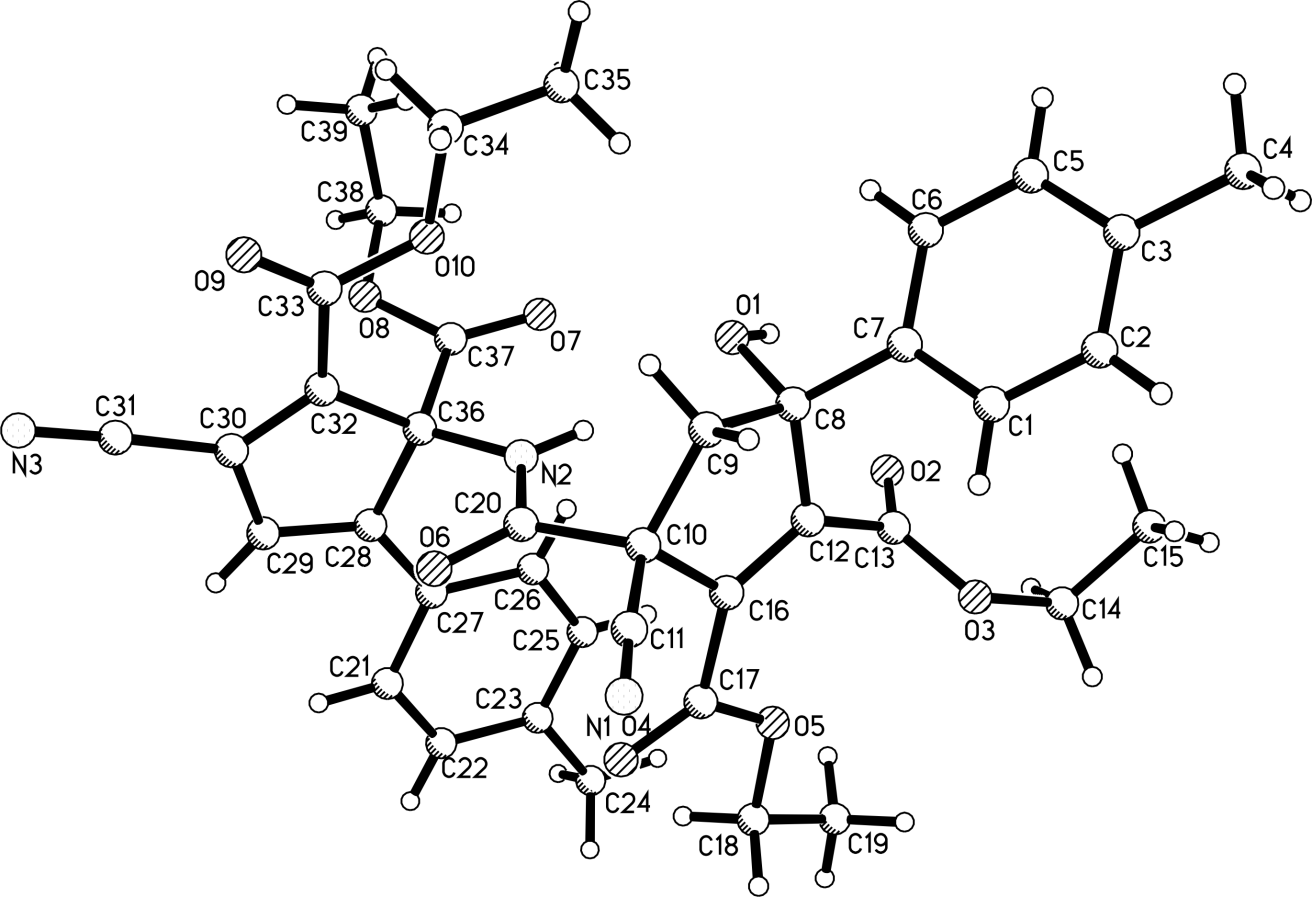
Single crystal structure of compound **4c**.

In order to obtain the corresponding products with elimination of water, alternative conditions for the base-promoted reaction of phenacylmalononitrile and dialkyl but-2-ynedioates were tested. After carefully examining the reaction conditions, we found that the reaction of phenacylmalononitriles and dialkyl but-2-ynedioates in acetonitrile at room temperature gave the unexpected products **4a–k** in moderate to good yields in the presence of 1,4-diazabicyclo[2.2.2]octane (DABCO) as base promoter and the results are summarized in Table 3. It can be seen that the products **4a–k** contain two scaffolds of each phenacylmalononitrile and dialkyl but-2-ynedioate. Thus, a quasi-four-component reaction led to the final product. There are two chiral carbon atoms in the molecules, thus two diastereoisomers would be formed in the reaction. However, the ^1^H and ^13^C NMR spectra gave only one set of resonances for the characteristic groups, which clearly indicated that only one diastereoisomer was actually produced in the reaction. The chemical structures of the compounds **4a**–**k** were established by various spectroscopy methods. Additionally, the single crystal structures of compounds **4a** and **4c** were successfully determined (Figure 2 and Figure 3). From the two figures, it can be seen that a cyclopentadiene moiety is connected to a cyclopent-1-ene moiety by a carboxamide unit (CONH). Although one hydroxy group is eliminated to give the cyclopentadiene ring, another hydroxy group is still present in the cyclopent-1-ene ring. Additionally, in the cyclopent-1-ene ring, the aryl group and the cyano group are in *cis*-orientation, while the hydroxy group and carboxamide group exist on the other side of the ring. On the basis of the NMR spectra and the single crystal structures, it can be concluded that all the obtained products **4a–k** have this kind of relative configuration.

**Table 3:** Synthesis of compounds **4a**–**k**.^a^

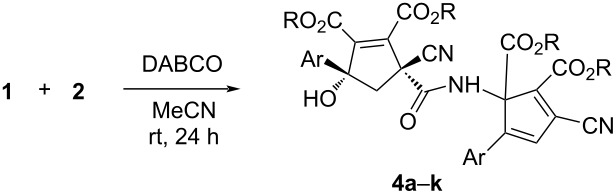				

Entry	Product	Ar	R	Yield (%)^b^

1	**4a**	*p-*CH_3_OC_6_H_4_	Et	62
2	**4b**	*m-*CH_3_OC_6_H_4_	Et	40
3	**4c**	*p-*CH_3_C_6_H_4_	Et	60
4	**4d**	C_6_H_5_	Et	56
5	**4e**	*p-*FC_6_H_4_	Et	35
6	**4f**	*p*-ClC_6_H_4_	Et	42
7	**4g**	*p-*BrC_6_H_4_	Et	46
8	**4h**	*p-*CH_3_OC_6_H_4_	CH_3_	54
9	**4i**	C_6_H_5_	CH_3_	53
10	**4j**	*p*-ClC_6_H_4_	CH_3_	38
11	**4k**	*p-*BrC_6_H_4_	CH_3_	42

^a^Reaction conditions: phenacylmalononitrile (0.5 mmol), dialkyl but-2-ynedioate (0.6 mmol), DABCO (1.0 mmol), CH_3_CN (5.0 mL), rt, 24 h. ^b^Isolated yields.

**Scheme 2:**
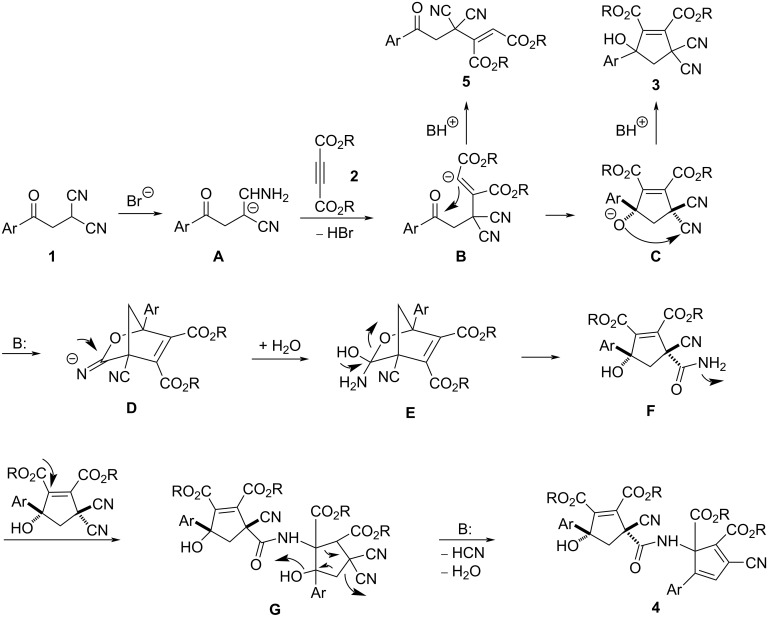
Proposed reaction mechanism for compounds **3**, **4**, and **5**.

**Figure 4:**
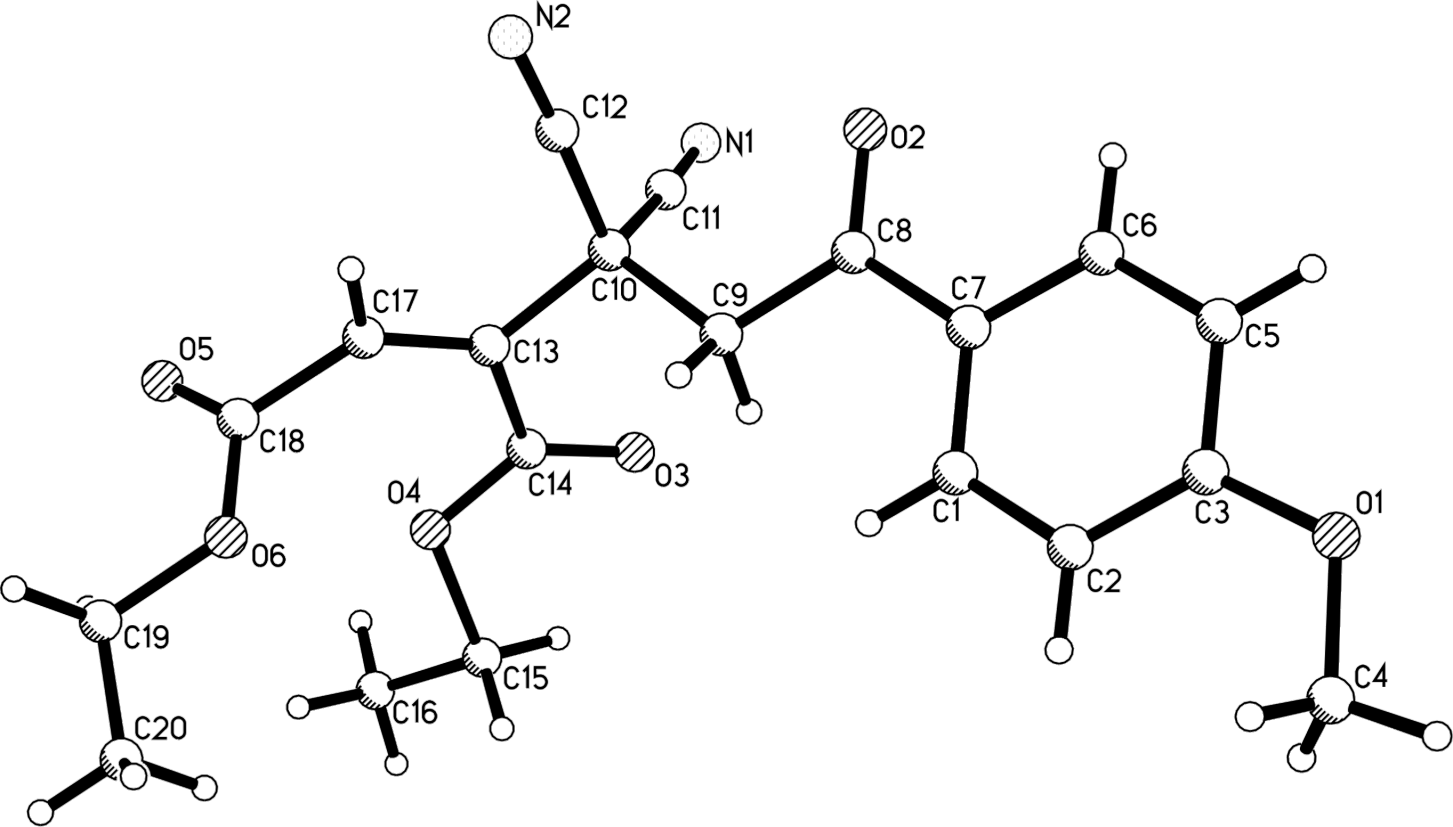
Single crystal structure of compound **5**.

For explaining the formation of the two kinds of the compounds, a plausible reaction mechanism was proposed on the basis of the previous works [30–35] and the present experimental results (Scheme 2). As described in the previous work, TBAF can act as an effective base to catalyze the C–C bond formation via a Michael addition of active methylene groups [31]. Therefore, in the presence of TBAB, the bromide assisted with the deprotonation of the phenacylmalononitrile to give a carbanion intermediate **A**. Secondly, the nucleophilic addition of carbanion **A** to electron-deficient alkyne resulted in adduct **B**. Thirdly, the intramolecular addition of the carbanion to the carbonyl group afforded species **C**, which in turn converted to the final product **3** by the protonation of the species **C**. The protonated species **5** could be successfully isolated in 12% yield after six hours when the reaction was carried out in weak basic solution (Supporting Information File 1) and its single crystal structure was determined by X-ray diffraction (Figure 4). When DABCO was used as a base, the further addition of the alkoxide ion to the cyano group in *cis*-position of the cyclopentyl ring produced a bridged cyclic 2-oxabicyclo[2.2.1]hept-5-ene **D**. The further hydration of intermediate **D** gave intermediate **E**, which was in turn transferred to cyclopentenyl intermediate **F** by the ring opening of the bridge ring. In the cyclopentenyl intermediate **F**, the hydroxy group and the amide group clearly exist in *cis-*position. Sequentially, nucleophilic addition of the amino group of intermediate **F** to the C–C double bond connecting the two alkoxycarbonyl groups in molecule **3** resulted in the formation of intermediate **G**. At last, the base-catalyzed dehydration and elimination of hydrocyanide gave the final product **4**. It is due to the in situ formation and ring-opening of 2-oxabicyclo[2.2.1]hept-5-ene, that only one diastereoisomer **4** with the aryl group and the remaining cyano group in *cis*-position was selectively formed in the domino reaction process.

**Scheme 3:**
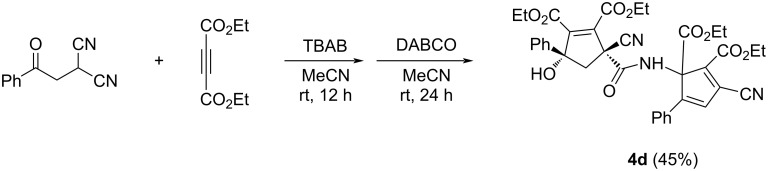
Control experiment.

In order to shed light on the proposed reaction mechanism, some control experiments were carried out. After finishing the reaction of phenacylmalononitrile and diethyl but-2-ynedioate in the presence of TBAB under the standard reaction conditions, DABCO was directly added to the reaction system (Scheme 3). The further reaction at room temperature for 24 hours afforded the expected product **4d** in 45% yield. This result clearly showed that the initially formed functionalized cyclopentene **3b** could be smoothly transferred to the carboxamide-bridged dicyclopentene **4d**, which also strongly supported the above proposed reaction mechanism.

## Conclusion

In summary, we have investigated the cycloaddition of phenacylmalononitriles and dialkyl but-2-ynedioates under different reaction conditions and identified convenient synthetic protocols for the synthesis of functionalized cyclopent-2-ene and complex dicyclopentene derivatives in satisfactory yields. The stereochemistry of the reactions was clearly elucidated and a rational reaction mechanism was proposed. The reactions have the advantages of using readily available reagents, mild reaction conditions, good yields, and high diastereoselectivity, and have potential synthetic applications in organic and medicinal chemistry.

## Experimental

**1. General procedure for the preparation of functionalized cyclopent-2-enes 3a**–**l:** To a round-bottomed flask was added phenacylmalononitrile (0.5 mmol), dialkyl but-2-ynedioate (0.6 mmol), tetrabutylammonium bromide (0.25 mmol), and acetonitrile (5.0 mL). The solution was stirred at room temperature for twelve hours. After removing the solvent by rotatory evaporation at reduced pressure, the residue was subjected to column chromatography with a mixture of ethyl acetate, petroleum ether and methylene dichloride 1:9:3 (v/v/v) as eluent to give the pure product for analysis.

**Diethyl 3,3-dicyano-5-hydroxy-5-(*p*-tolyl)cyclopent-1-ene-1,2-dicarboxylate (3a)**: white solid, 85%; mp 151–153 °C; ^1^H NMR (400 MHz, CDCl_3_) δ 7.29–7.27 (m, 2H, ArH), 7.21–7.20 (m, 2H, ArH), 4.47–4.41 (m, 2H, OCH_2_), 4.21–4.17 (m, 2H, OCH_2_), 3.52 (s, 1H, OH), 3.23 (d, *J* = 14.4 Hz, 1H, CH_2_), 2.96 (d, *J* = 14.4 Hz, 1H, CH_2_), 2.36 (s, 3H, CH_3_), 1.41 (t, *J* = 6.8 Hz, 3H, CH_3_), 1.14 (t, *J* = 6.8 Hz, 3H, CH_3_); ^13^C NMR (100 MHz, CDCl_3_) δ 163.2, 159.2, 152.7, 139.0, 136.8, 129.6, 129.5, 124.7, 113.7, 113.5, 86.9, 63.2, 62.7, 52.3, 38.1, 21.1, 13.9, 13.7; HRMS–ESI (*m*/*z*): [M + Na]^+^ calcd for C_20_H_20_NaN_2_O_5_, 391.1270; found, 391.1271.

**2. General procedure for the preparation of functionalized carboxamide-bridged dicyclopentenes 4a–k:** To a round-bottomed flask was added phenacylmalononitrile (0.5 mmol), dialkyl but-2-ynedioate (0.6 mmol), DABCO (1.0 mmol), and acetonitrile (5.0 mL). The solution was stirred at room temperature for 24 hours. After removing the solvent by rotatory evaporation at reduced pressure, the residue was subjected to column chromatography with a mixture of ethyl acetate and petroleum ether 1:3 (v/v) as eluent to give the pure product for analysis.

**Diethyl 3-cyano-1-(1-cyano-2,3-bis(ethoxycarbonyl)-4-hydroxy-4-(4-methoxyphenyl)cyclopent-2-ene-1-carboxamido)-5-(4-methoxyphenyl)cyclopenta-2,4-diene-1,2-dicarboxylate (4a)**: yellow solid, 62%; mp 182–184 °C; ^1^H NMR (400 MHz, CDCl_3_) δ 8.92 (s, 1H, NH), 7.46 (d, *J* = 8.4 Hz, 2H, ArH), 7.34 (d, *J* = 8.4 Hz, 2H, ArH), 6.90 (s, 1H, CH), 6.88–6.84 (m, 4H, ArH), 4.62 (s, 1H, OH), 4.35–4.30 (m, 2H, OCH_2_), 4.27–4.19 (m, 3H, OCH_2_), 4.13–4.03 (m, 3H, OCH_2_), 3.81 (s, 3H, OCH_3_), 3.79 (s, 3H, OCH_3_), 2.74 (d, *J* = 14.8 Hz, 1H, CH_2_), 2.47 (d, *J* = 14.8 Hz, 1H, CH_2_), 1.39 (t, *J* = 7.2 Hz, 3H, CH_3_), 1.28 (t, *J* = 7.2 Hz, 3H, CH_3_), 1.15 (t, *J* = 7.2 Hz, 3H, CH_3_), 1.06 (t, *J* = 7.2 Hz, 3H, CH_3_); ^13^C NMR (100 MHz, CDCl_3_) δ 166.5, 164.2, 163.4, 161.1, 160.4, 159.5, 159.4, 153.7, 150.8, 142.6, 131.8, 129.9, 127.8, 126.9, 125.9, 124.8, 122.9, 117.5, 114.7, 113.6, 113.4, 86.0, 72.7, 64.3, 62.5, 62.0, 61.8, 55.4, 55.3, 52.9, 51.4, 13.9, 13.8, 13.7, 13.6; HRMS–ESI (*m*/*z*): [M + Na]^+^ calcd for C_39_H_39_NaN_3_O_12_, 764.2431; found, 764.2413.

## Supporting Information

The crystallographic data of the compounds **3k** (CCDC 2176733), **4a** (CCDC 2176734), **4b** (CCDC 2176735), and **5** (CCDC 2176736) have been deposited at the Cambridge Crystallographic Database Center (http://www.ccdc.cam.ac.uk).

File 1 Characterization data and ^1^H NMR, ^13^C NMR, and HRMS spectra of the compounds.
