# Supplementary material for: Molecular diversity of the base-promoted reaction of phenacylmalononitriles with dialkyl but-2-ynedioates
Source: Beilstein J Org Chem. 2022 Aug 8;18:991–8. doi: 10.3762/bjoc.18.99 (PMC9379639; doi:10.3762/bjoc.18.99)
Supplement: File 1 — Characterization data and 1H NMR, 13C NMR, and HRMS spectra of the compounds. [file Beilstein_J_Org_Chem-18-991-s001.pdf]

BEILSTEIN JOURNAL OF ORGANIC CHEMISTRY

Supporting Information

for

Molecular diversity of the base-promoted reaction of phenacylmalononitriles with dialkyl but-2-ynedioates

Hui Zheng, Ying Han, Jing Sun and Chao-Guo Yan

Beilstein J. Org. Chem. **2022,** *18,* 991–998. doi:10.3762/bjoc.18.99

Characterization data and ^{1}H NMR, ^{13}C NMR, and HRMS spectra of the compounds

License and Terms: This is a supporting information file under the terms of the Creative Commons Attribution License (https://creativecommons.org/licenses/by/4.0). Please note that the reuse, redistribution and reproduction in particular requires that the author(s) and source are credited and that individual graphics may be subject to special legal provisions.
The license is subject to the *Beilstein Journal of Organic Chemistry* terms and conditions: (https://www.beilstein-journals.org/bjoc/terms)

Diethyl 3,3-dicyano-5-hydroxy-5-(*p*-tolyl)cyclopent-1-ene-1,2-dicarboxylate (3a):

For characterization data set, see main text.

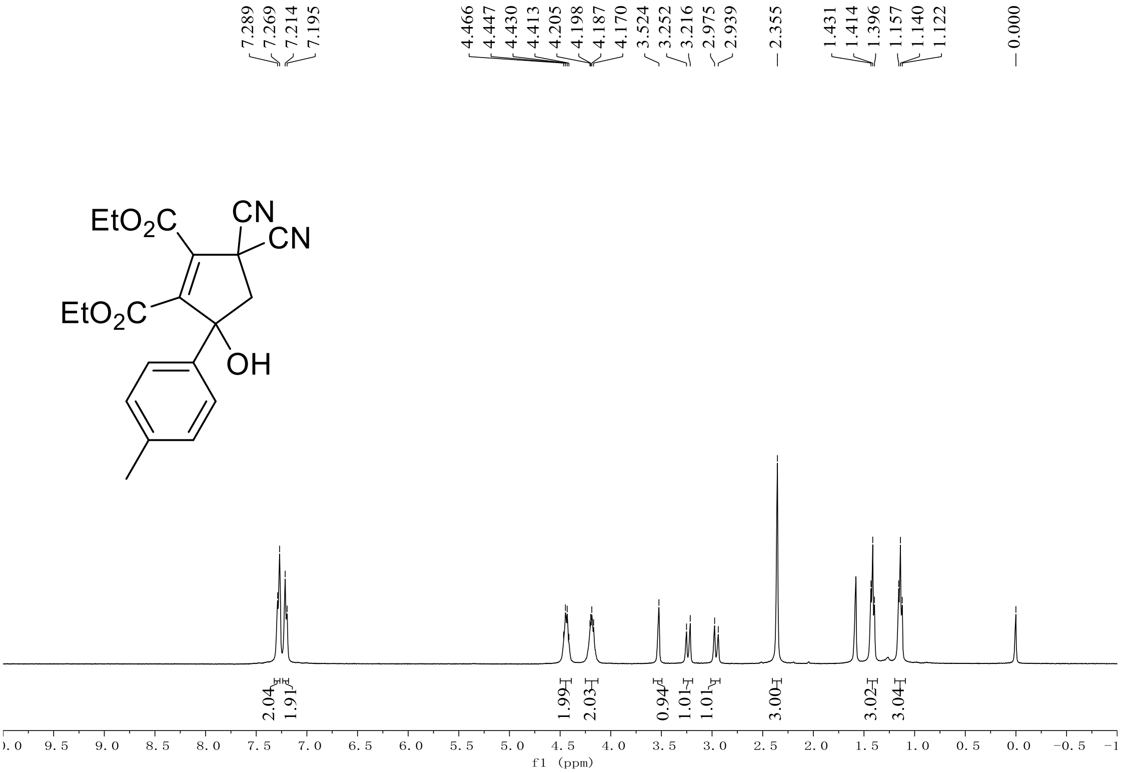

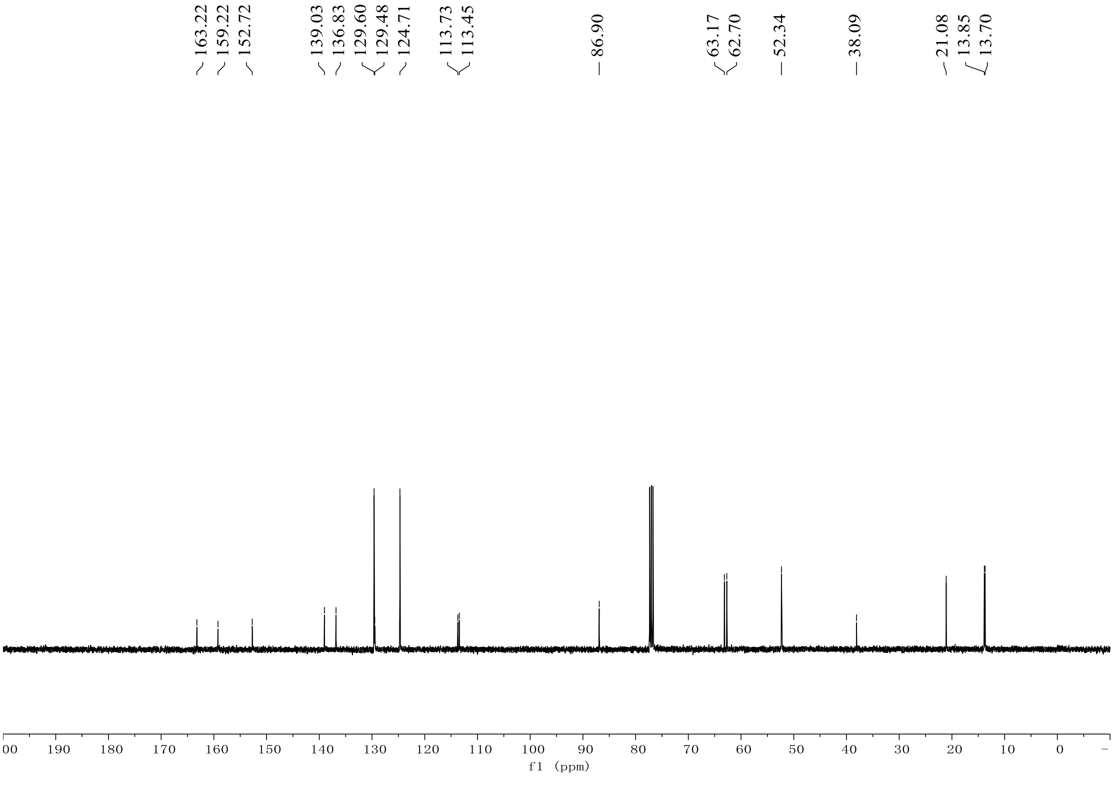

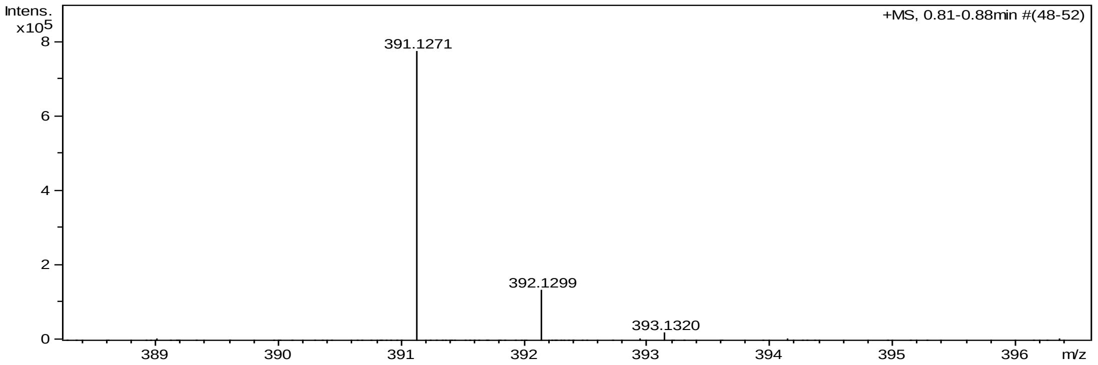

Diethyl 3,3-dicyano-5-hydroxy-5-phenylcyclopent-1-ene-1,2-dicarboxylate (3b): white solid, 75%, m.p. 97-99 °C; ^{1}H NMR (400 MHz, $CDCl_3$) δ 7.41-7.40 (m, 4H, ArH), 7.38-7.35 (m, 1H, ArH), 4.47-4.42 (m, 2H, OCH_2), 4.21-4.13 (m, 2H, OCH_2), 3.63 (s, 1H, OH), 3.26 (d, J = 14.4 Hz, 1H, CH_2), 2.99 (d, J = 14.4 Hz, 1H, CH_2), 1.41 (t, J = 7.2 Hz, 3H, CH_3), 1.11 (t, J = 7.2 Hz, 3H, CH_3). ^{13}C NMR (100 MHz, $CDCl_3$) δ 163.2, 159.2, 152.5, 139.8, 129.7, 129.1, 129.0, 124.8, 113.7, 113.4, 89.0, 63.2, 62.8, 52.3, 38.2, 13.9, 13.7. MS (m/z): HRMS (ESI) Calcd. for $C_{19}H_{18}NaN_2O_5$ ([M+Na]$^+$): 377.1113, found: 377.1116.

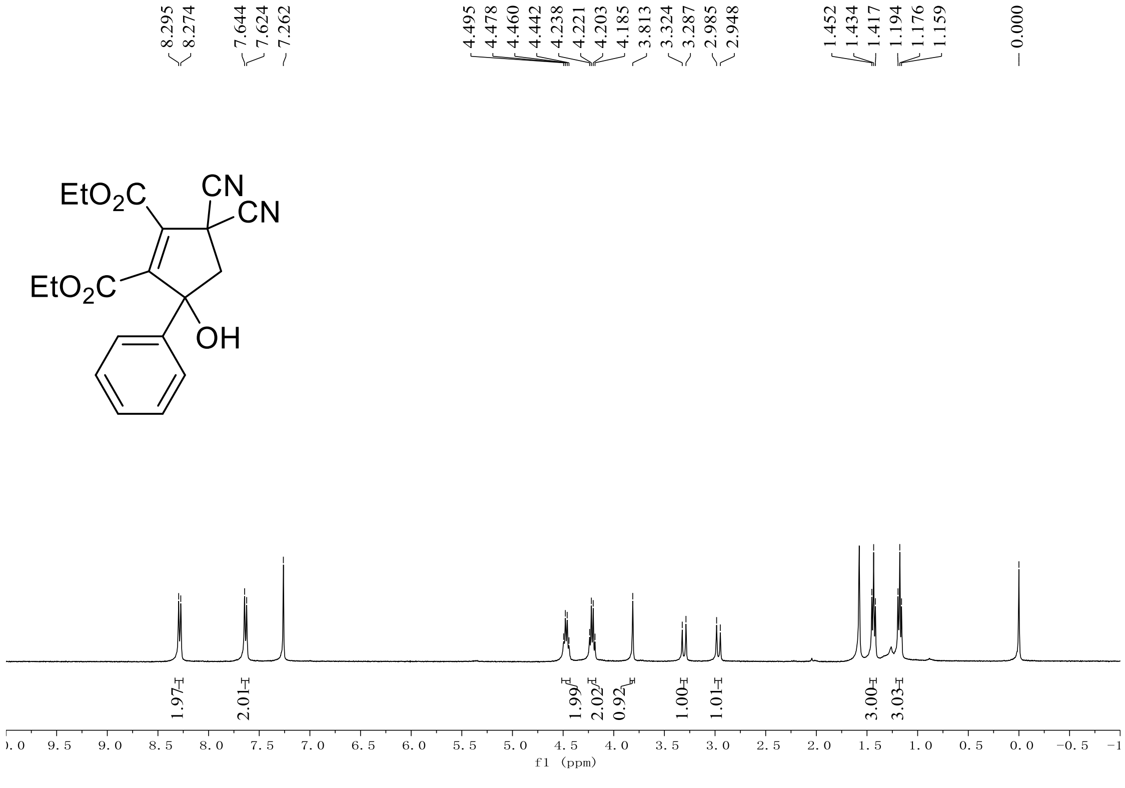

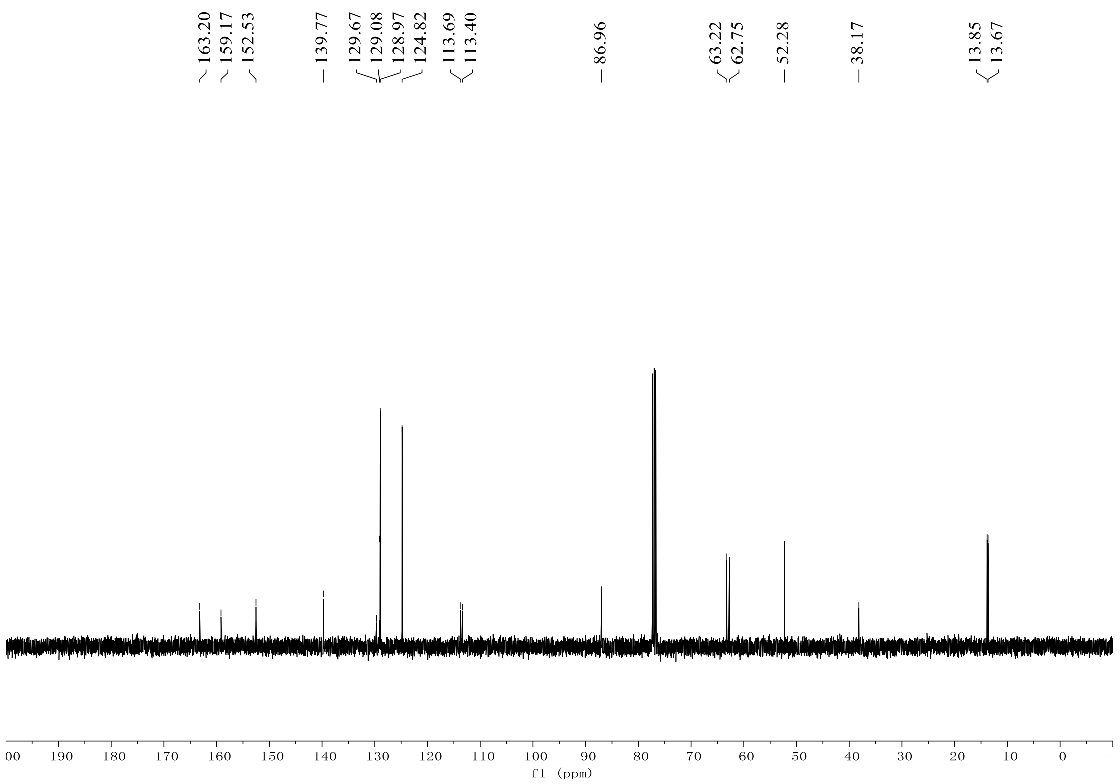

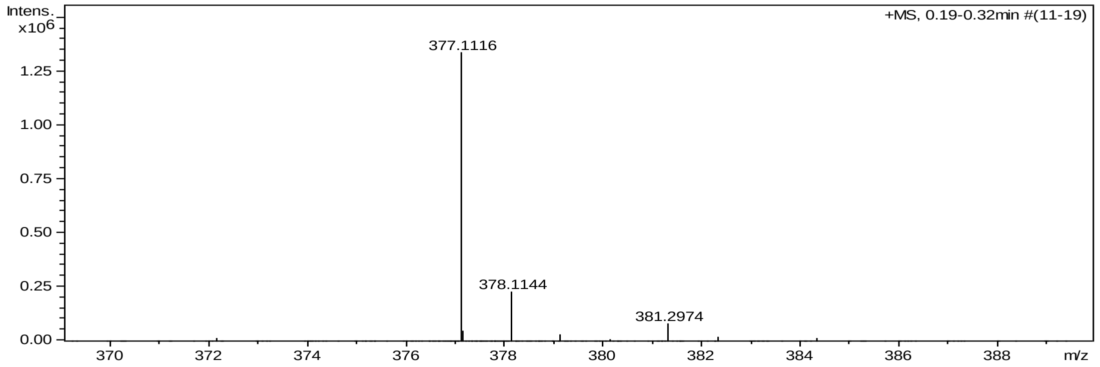

Diethyl 3,3-dicyano-5-hydroxy-5-(3-methoxyphenyl)cyclopent-1-ene-1,2-dicarboxylate (3c): white solid, 73%, m.p. 127-128 °C; ^{1}H NMR (400 MHz, $CDCl_3$) δ 7.31 (m, 1H, ArH), 6.97-6.96 (m, 1H, ArH), 6.93 (t, *J* = 8.0 Hz, 1H, ArH), 6.90-6.87 (m, 1H, ArH), 4.48-4.40 (m, 2H, OCH_2), 4.25-4.16 (m, 2H, OCH_2), 3.82(s, 3H, OCH_3), 3.60 (s, 1H, OH), 3.24 (d, *J* = 14.4 Hz, 1H, CH_2), 2.97 (d, *J* = 14.4 Hz, 1H, CH_2), 1.42 (t, *J* = 7.2 Hz, 3H, CH_3), 1.15 (t, *J* = 7.2 Hz, 3H, CH_3). ^{13}C NMR (100 MHz, $CDCl_3$) δ 163.2, 160.1, 159.2, 152.3, 141.5, 130.2, 129.8, 117.0, 114.6, 113.7, 113.4, 110.4, 86.9, 63.2, 62.8, 55.4, 52.2, 38.2, 13.8, 13.7. MS (*m*/*z*): HRMS (ESI) Calcd. for $C_{20}H_{20}NaN_2O_6$ ($[M+Na]^+$): 407.1219, found: 407.1220.

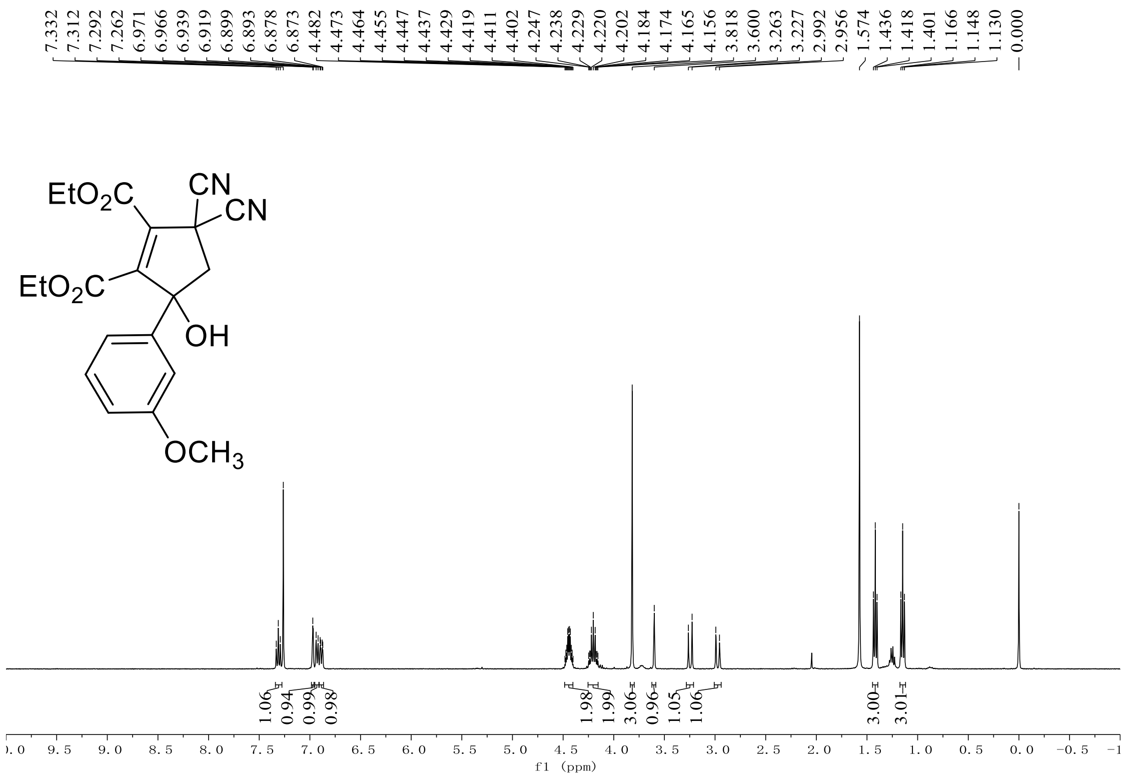
7.332
7.312
7.292
7.262
6.971
6.966
6.939
6.919
6.899
6.893
6.878
6.873
4.482
4.473
4.464
4.455
4.447
4.437
4.429
4.419
4.411
4.402
4.247
4.238
4.229
4.220
4.202
4.184
4.174
4.165
4.156
3.818
3.600
3.263
3.227
2.992
2.956
1.574
1.436
1.418
1.401
1.166
1.148
1.130
0.000
EtO2C
CN
CN
EtO2C
OH
OCH3
1.06
0.94
0.99
0.98
1.98
1.99
3.06
0.96
1.05
1.06
3.00
3.01
f1 (ppm)

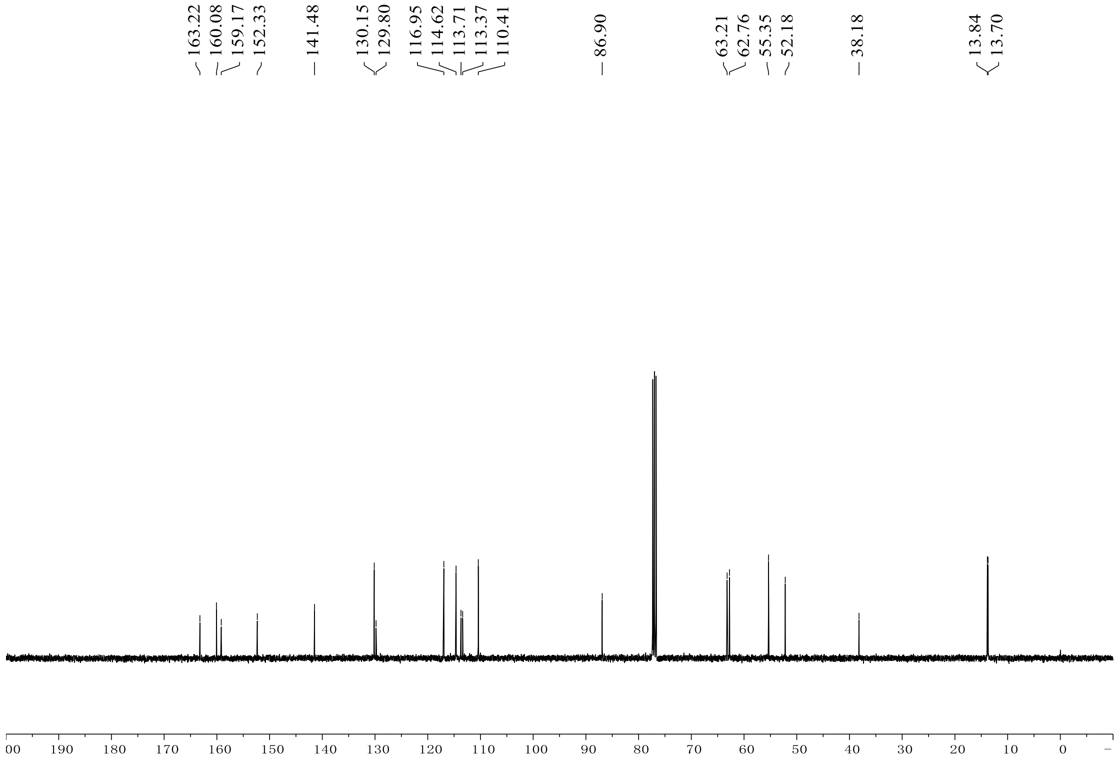
163.22
160.08
159.17
152.33
141.48
130.15
129.80
116.95
114.62
113.71
113.37
110.41
86.90
63.21
62.76
55.35
52.18
38.18
13.84
13.70
f1 (ppm)

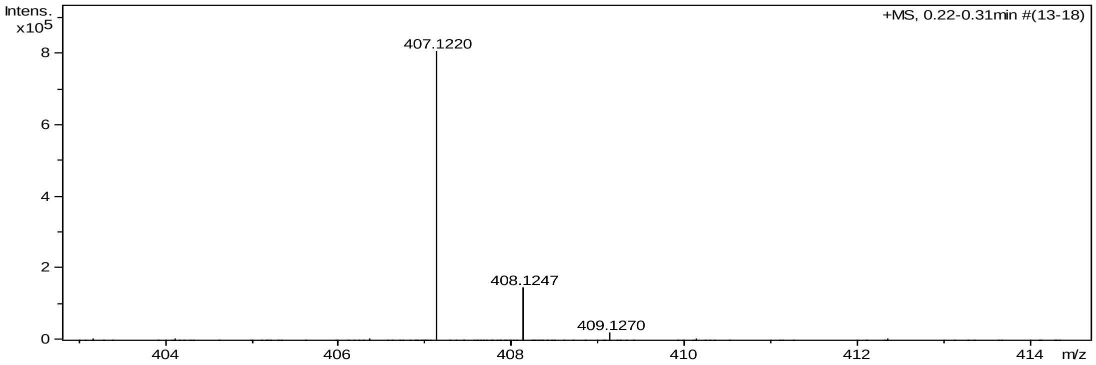

Diethyl 3,3-dicyano-5-hydroxy-5-(4-methoxyphenyl)cyclopent-1-ene-1,2-dicarboxylate (3d): white solid, 88%, m.p. 151-152 °C; ^{1}H NMR (400 MHz, $CDCl_3$) δ 7.32 (d, *J* = 8.8 Hz, 2H, ArH), 6.92 (d, *J* = 8.8 Hz, 2H, ArH), 4.47-4.41 (m, 2H, OCH_2), 4.24-4.15 (m, 2H, OCH_2), 3.82 (s, 3H, OCH_3), 3.50 (s, 1H, OH), 3.23 (d, *J* = 14.4 Hz, 1H, CH_2), 2.95 (d, *J* = 14.4 Hz, 1H, CH_2), 1.41 (t, *J* = 7.2 Hz, 3H, CH_3), 1.15 (t, *J* = 7.2 Hz, 3H, CH_3). ^{13}C NMR (100 MHz, $CDCl_3$) δ 163.3, 160.0, 159.2, 152.8, 131.7, 129.3, 126.2, 114.2, 113.7, 113.5, 86.7, 63.2, 62.7, 55.3, 52.4, 38.0, 13.9, 13.7. MS (*m/z*): HRMS (ESI) Calcd. for $C_{20}H_{20}NaN_2O_6$ ($[M+Na]^+$): 407.1219, found: 407.1221.

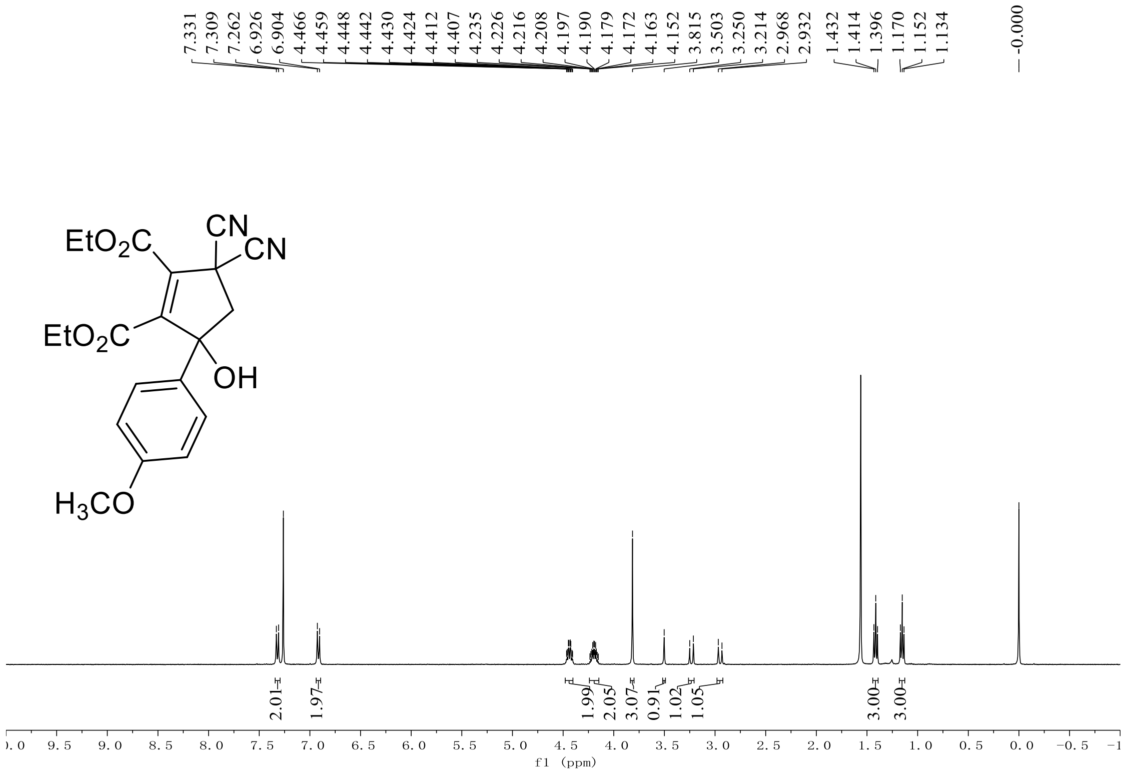

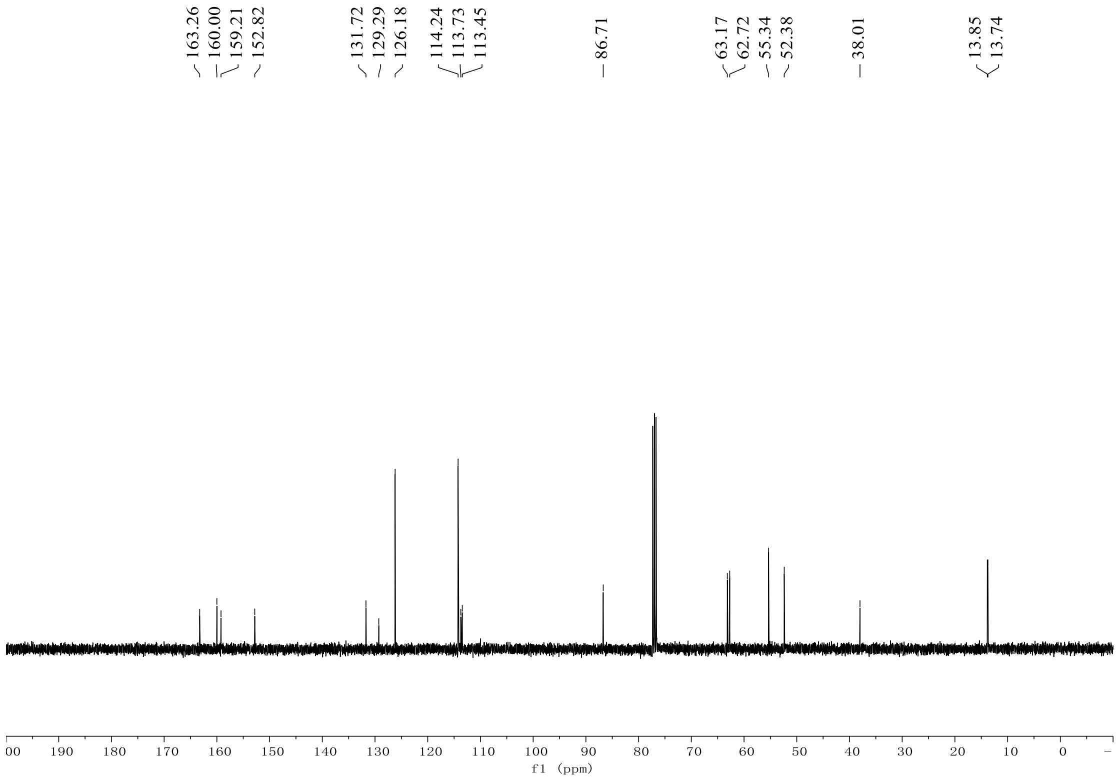

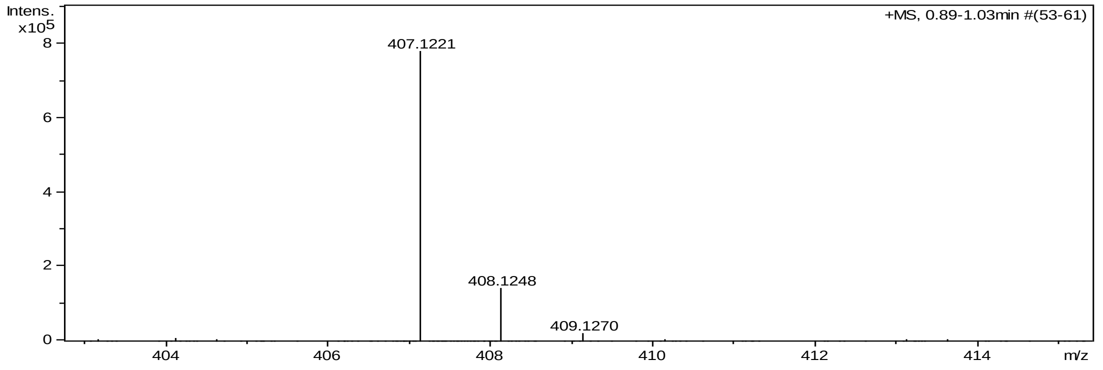

Diethyl 3,3-dicyano-5-hydroxy-5-(2-methoxyphenyl)cyclopent-1-ene-1,2-dicarboxylate (3e): white solid, 62%, m.p. 154-156 °C; ^{1}H NMR (400 MHz, $CDCl_3$) δ 7.59 (d, *J* = 7.6 Hz, 1H, ArH), 7.36 (d, *J* = 8.0 Hz, 1H, ArH), 7.02 (t, *J* = 7.6 Hz, 1H, ArH), 6.89 (d, *J* = 8.0 Hz, 1H, ArH), 4.45-4.38 (m, 2H, OCH_2), 4.18-4.06 (m, 2H, OCH_2), 3.83 (s, 3H, OCH_3), 3.63 (s, 1H, OH), 3.32 (d, *J* = 14.4 Hz, 1H, CH_2), 3.05 (d, *J* = 14.4 Hz, 1H, CH_2), 1.40 (t, *J* = 7.2 Hz, 3H, CH_3), 1.04 (t, *J* = 7.2 Hz, 3H, CH_3). ^{13}C NMR (100 MHz, $CDCl_3$) δ 163.4, 159.7, 155.5, 154.7, 130.7, 126.7, 126.4, 120.9, 114.3, 114.1, 110.9, 85.3, 62.9, 62.3, 55.3, 49.7, 38.3, 13.8, 13.7. MS (*m*/*z*): HRMS (ESI) Calcd. for $C_{20}H_{20}NaN_2O_6$ ([M+Na]$^+$): 407.1219, found: 407.1222.

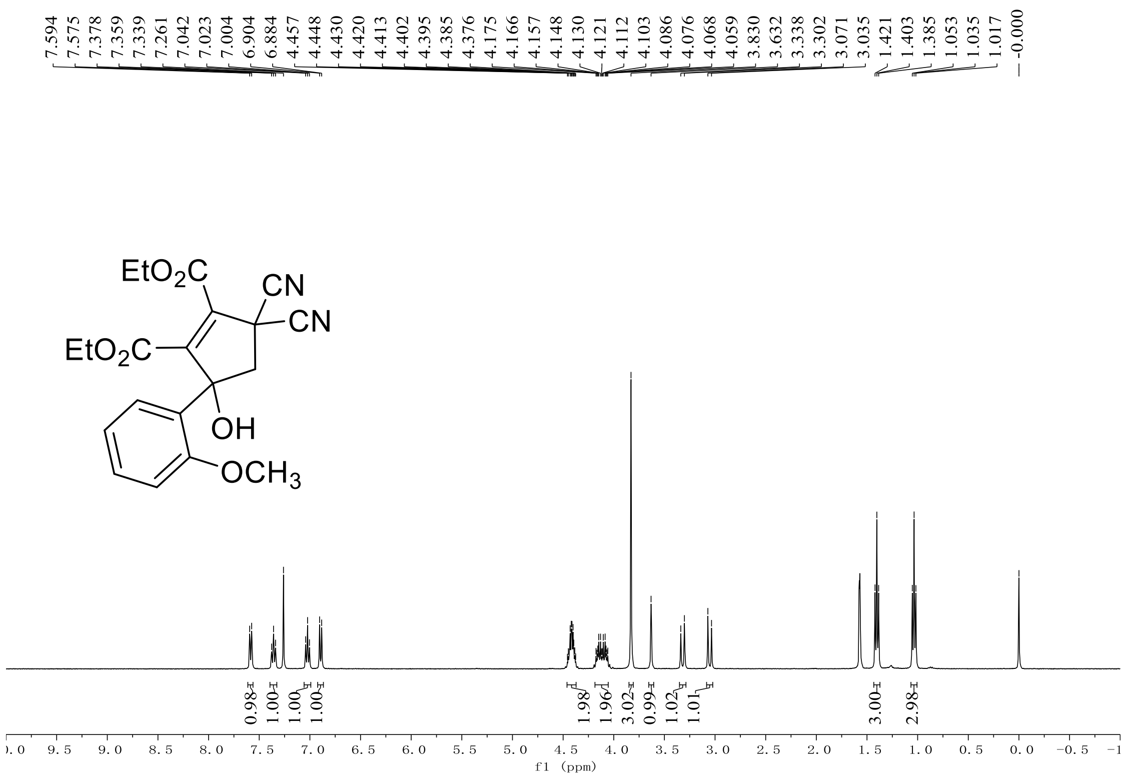
7.594 7.575 7.378 7.359 7.339 7.261 7.042 7.023 7.004 6.904 6.884 4.457 4.448 4.430 4.420 4.413 4.402 4.395 4.385 4.376 4.175 4.166 4.157 4.148 4.130 4.121 4.112 4.103 4.086 4.076 4.068 4.059 3.830 3.632 3.338 3.302 3.071 3.035 1.421 1.403 1.385 1.053 1.035 1.017 -0.000
EtO2C
CN
CN
EtO2C
OH
OCH3
0.98 1.00 1.00 1.00 1.98 1.96 3.02 0.99 1.02 1.01 3.00 2.98
9.5 9.0 8.5 8.0 7.5 7.0 6.5 6.0 5.5 5.0 4.5 4.0 3.5 3.0 2.5 2.0 1.5 1.0 0.5 0.0 -0.5
f1 (ppm)

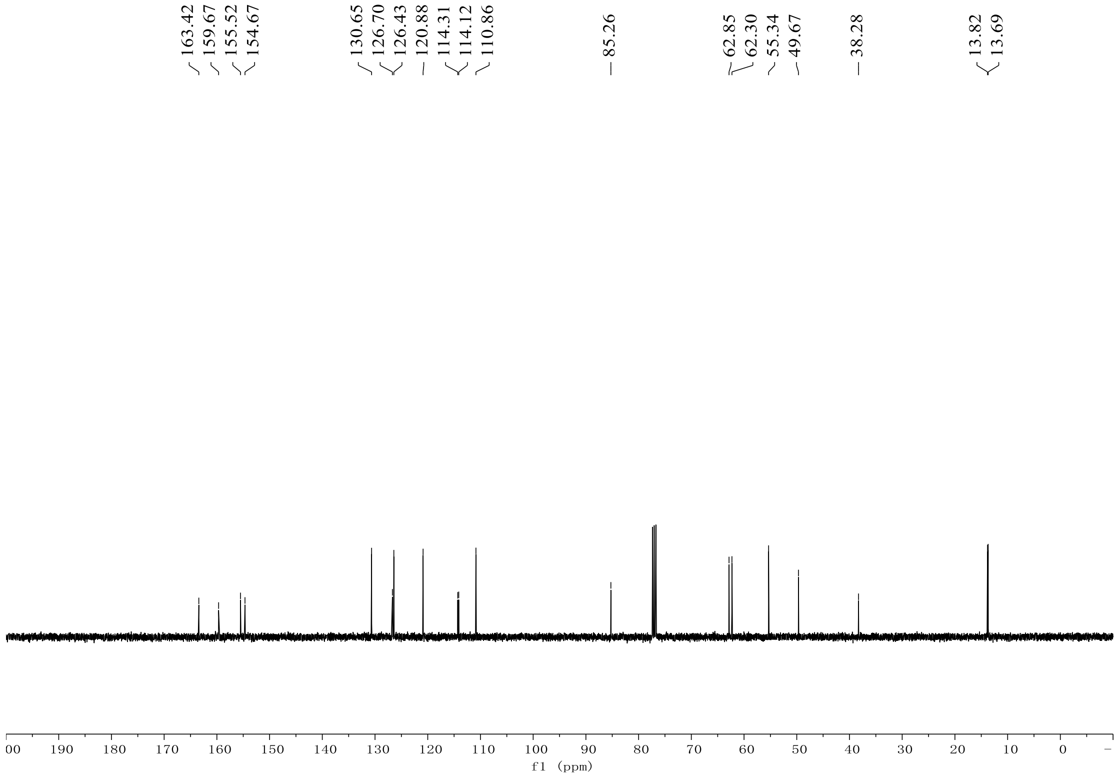
163.42 159.67 155.52 154.67 130.65 126.70 126.43 120.88 114.31 114.12 110.86 85.26 62.85 62.30 55.34 49.67 38.28 13.82 13.69
190 180 170 160 150 140 130 120 110 100 90 80 70 60 50 40 30 20 10 0
f1 (ppm)

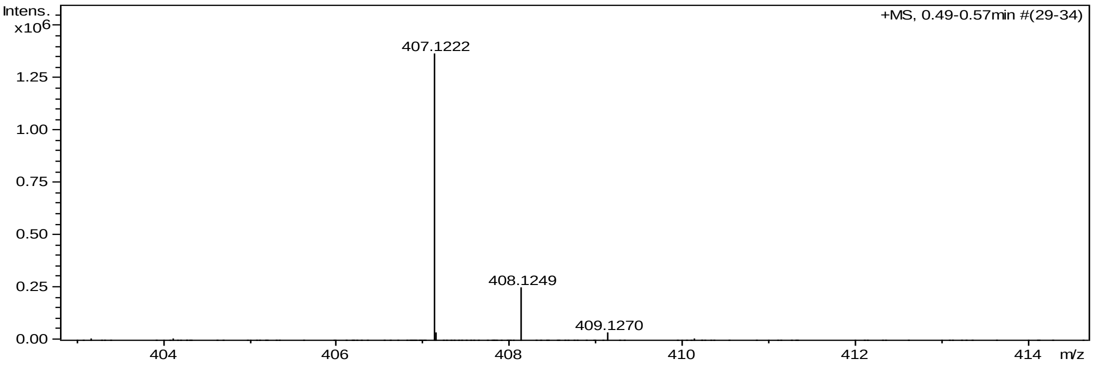

Diethyl 3-(4-chlorophenyl)-5,5-dicyano-3-hydroxycyclopent-1-ene-1,2-dicarboxylate (3f): white solid, 60%, m.p. 129-130 °C; ^{1}H NMR (400 MHz, $CDCl_3$) δ 7.40-7.34 (m, 4H, ArH), 4.48-4.42 (m, 2H, OCH_2), 4.23-4.18 (m, 2H, OCH_2), 3.62 (s, 1H, OH), 3.25 (d, J = 14.4 Hz, 1H, CH_2), 2.94 (d, J = 14.4 Hz, 1H, CH_2), 1.42 (t, J = 7.2 Hz, 3H, CH_3), 1.16 (t, J = 7.2 Hz, 3H, CH_3). ^{13}C NMR (100 MHz, $CDCl_3$) δ 163.1, 159.0, 151.8, 138.4, 135.2, 130.2, 129.2, 126.4, 113.5, 113.2, 86.5, 63.3, 63.0, 52.3, 38.1, 13.9, 13.7. MS (m/z): HRMS (ESI) Calcd. for $C_{19}H_{17}ClNaN_2O_5$ ([M+Na]$^+$): 411.0724, found: 411.0719.

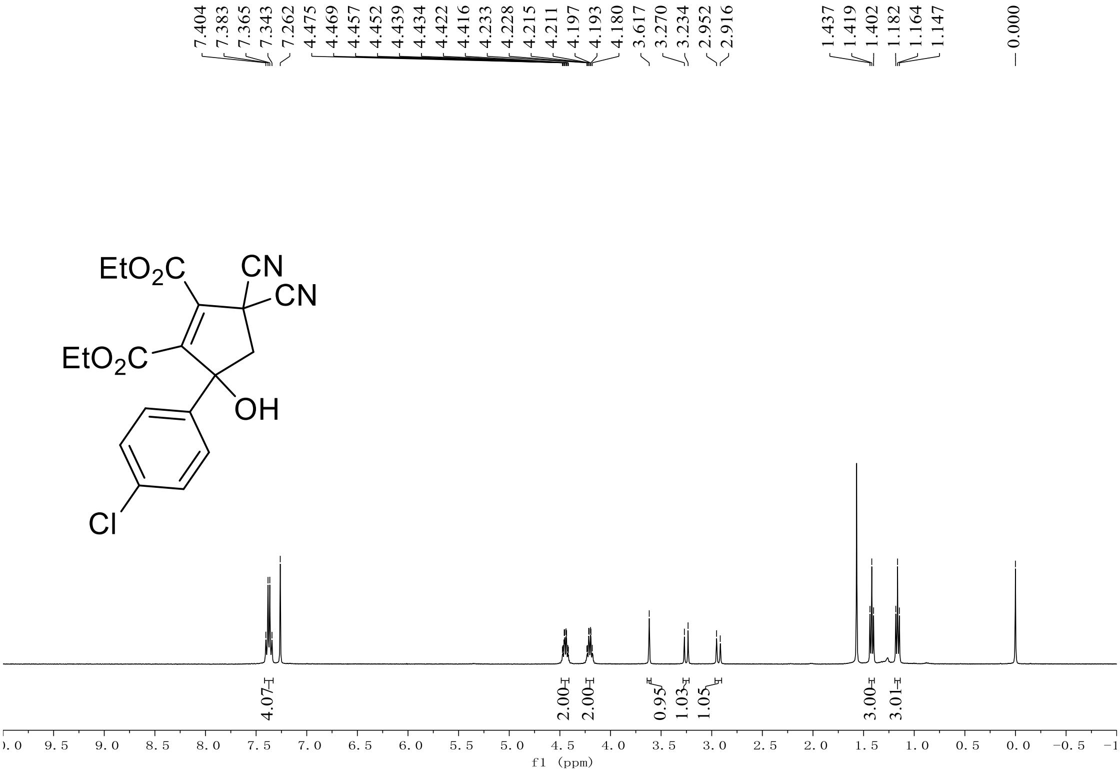

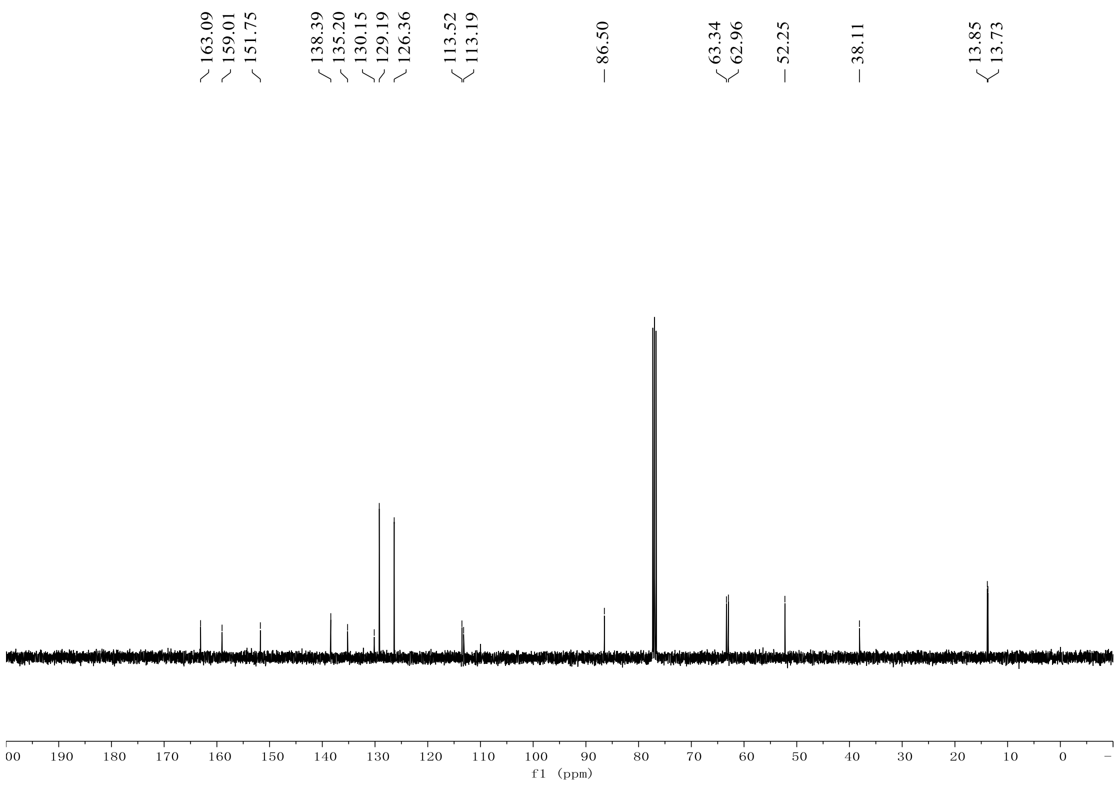

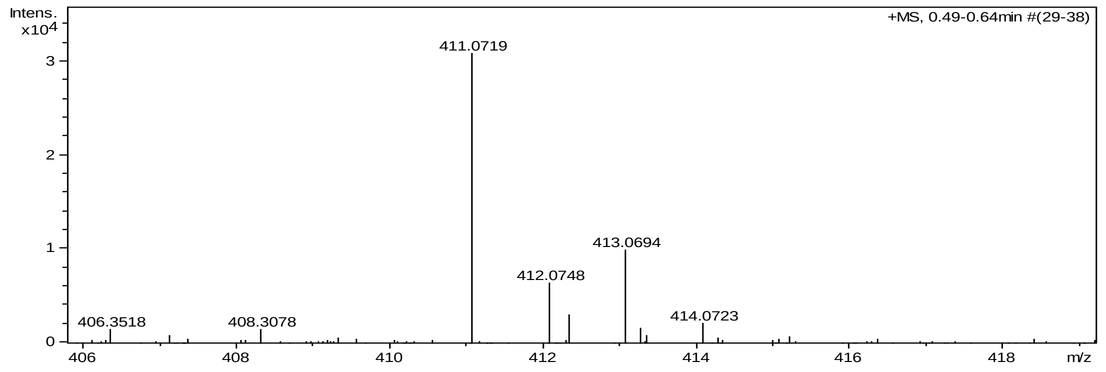

Diethyl 3-(4-bromophenyl)-5,5-dicyano-3-hydroxycyclopent-1-ene-1,2-dicarboxylate (3g): white solid, 62%, m.p. 149-150 °C; ^{1}H NMR (400 MHz, $CDCl_3$) δ 7.55 (d, *J* = 7.2 Hz, 2H, ArH), 7.29 (d, *J* = 7.2 Hz, 2H, ArH), 4.47-4.42 (m, 2H, OCH_2), 4.23-4.18 (m, 2H, OCH_2), 3.60 (s, 1H, OH), 3.25 (d, *J* = 14.4 Hz, 1H, CH_2), 2.93 (d, *J* = 14.4 Hz, 1H, CH_2), 1.42 (t, *J* = 7.2 Hz, 3H, CH_3), 1.17 (t, *J* = 7.2 Hz, 3H, CH_3). ^{13}C NMR (100 MHz, $CDCl_3$) δ 163.1, 159.0, 151.7, 138.9, 132.1, 130.1, 126.7, 123.4, 113.5, 113.2, 86.5, 63.3, 63.0, 52.2, 38.1, 13.9, 13.7. MS (*m*/*z*): HRMS (ESI) Calcd. for $C_{19}H_{17}BrNaN_2O_5$ ($[M+Na]^+$): 455.0219, found: 455.0245.

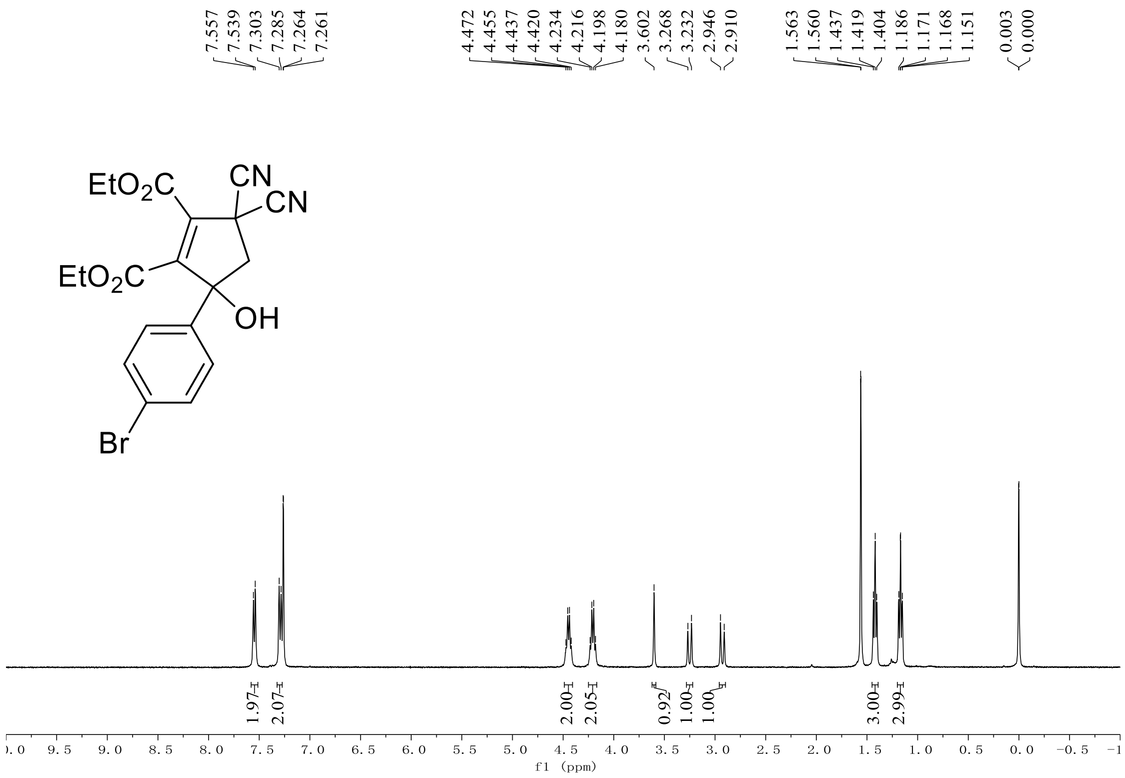
7.557
7.539
7.303
7.285
7.264
7.261
4.472
4.455
4.437
4.420
4.234
4.216
4.198
4.180
3.602
3.268
3.232
2.946
2.910
1.563
1.560
1.437
1.419
1.404
1.186
1.171
1.168
1.151
0.003
0.000
EtO2C
CN
CN
EtO2C
OH
Br
1.97
2.07
2.00
2.05
0.92
1.00
1.00
3.00
2.99
f1 (ppm)

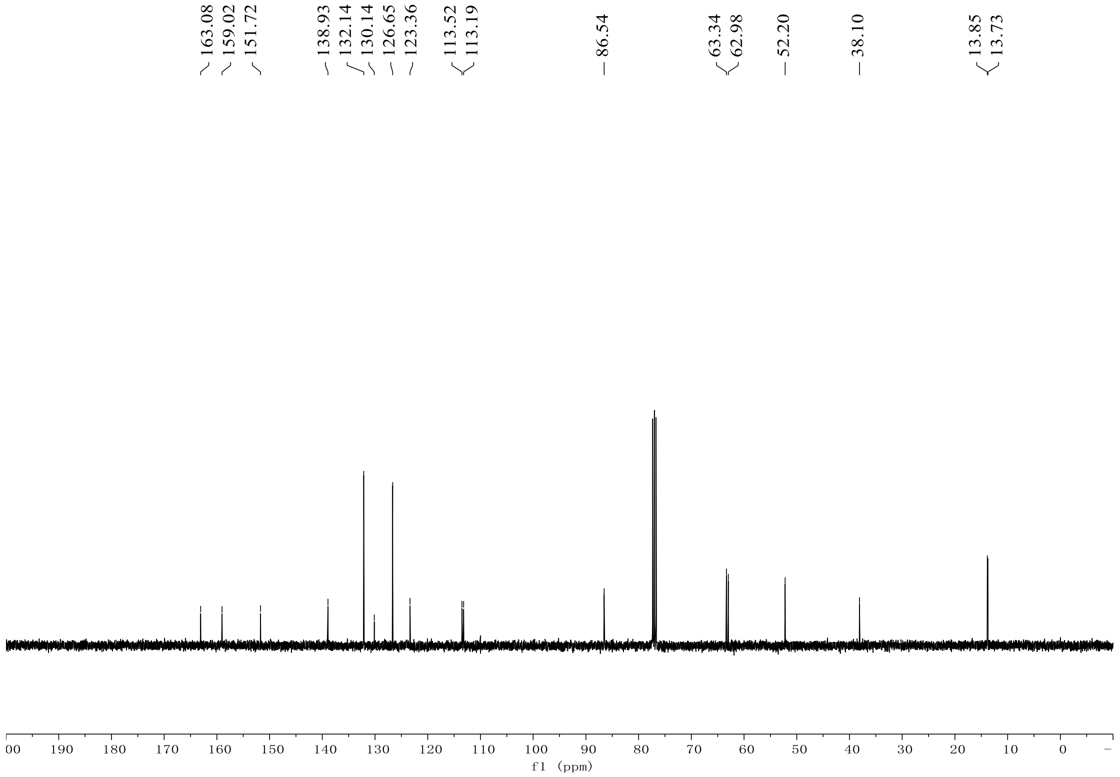
163.08
159.02
151.72
138.93
132.14
130.14
126.65
123.36
113.52
113.19
86.54
63.34
62.98
52.20
38.10
13.85
13.73
f1 (ppm)

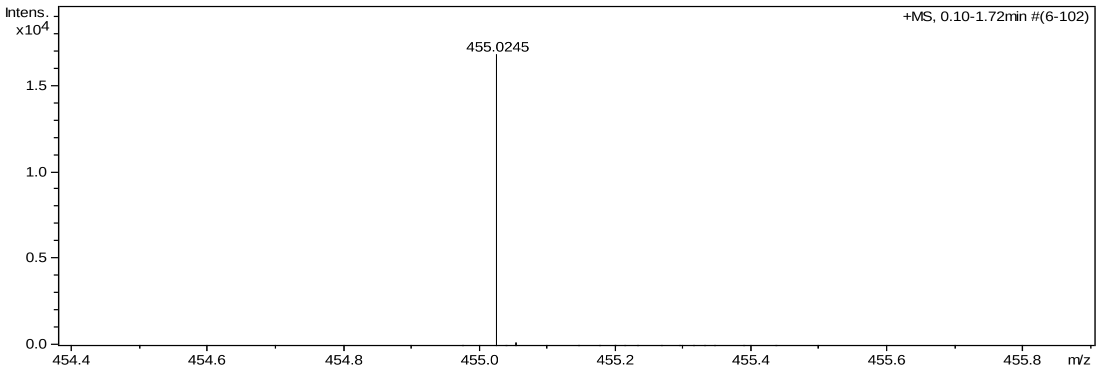

Diethyl 3,3-dicyano-5-hydroxy-5-(4-nitrophenyl)cyclopent-1-ene-1,2-dicarboxylate (3h): white solid, 56%, m.p. 139-140 °C; ^{1}H NMR (400 MHz, $CDCl_3$) δ 8.28 (d, *J* = 8.4 Hz, 2H, ArH), 7.63 (d, *J* = 8.0 Hz, 2H, ArH), 4.50-4.44 (m, 2H, OCH_2), 4.24-4.19 (m, 2H, OCH_2), 3.81 (s, 1H, OH), 3.30 (d, *J* = 14.8 Hz, 1H, CH_2), 2.96 (d, *J* = 14.8 Hz, 1H, CH_2), 1.43 (t, *J* = 7.2 Hz, 3H, CH_3), 1.18 (t, *J* = 7.2 Hz, 3H, CH_3). ^{13}C NMR (100 MHz, $CDCl_3$) δ 162.9, 158.8, 150.7, 148.3, 146.7, 131.0, 126.2, 124.2, 113.3, 112.9, 86.4, 63.5, 63.2, 52.2, 38.2, 13.8, 13.7. MS (*m/z*): HRMS (ESI) Calcd. for $C_{19}H_{17}NaN_3O_7$ ($[M+Na]^+$): 422.0964, found: 422.0957.

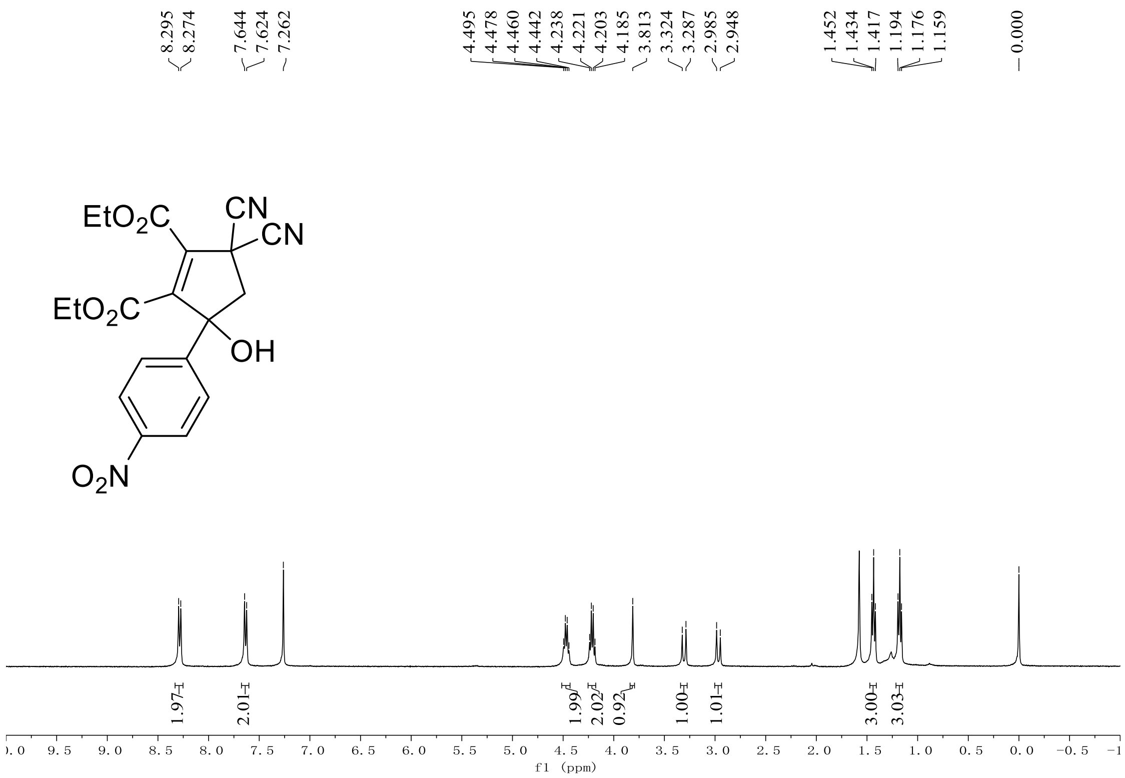

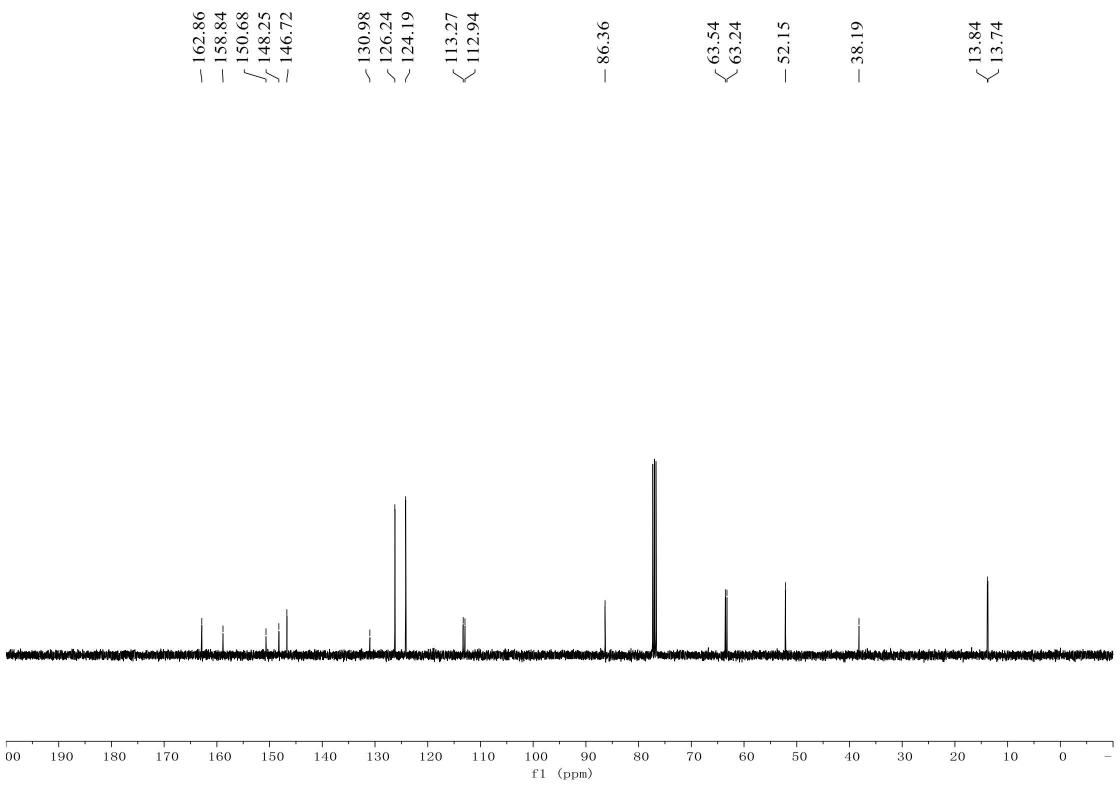

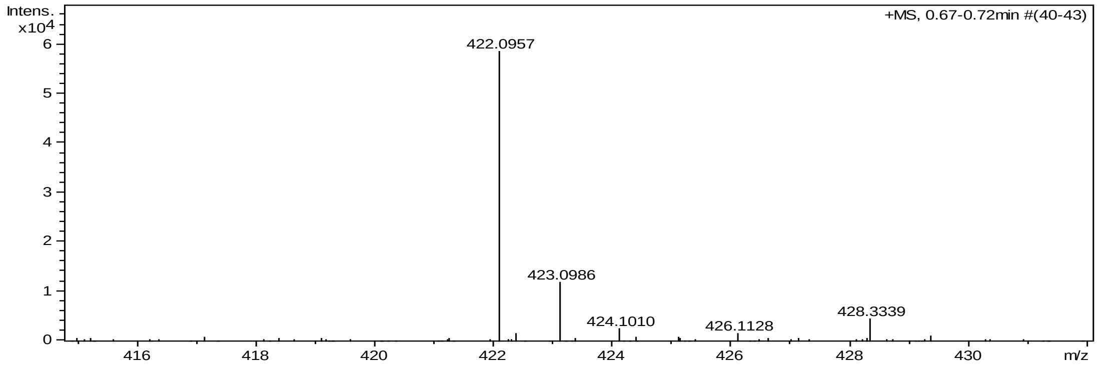

Dimethyl 3,3-dicyano-5-hydroxy-5-(*p*-tolyl)cyclopent-1-ene-1,2-dicarboxylate (3i): white solid, 78%, m.p. 141-142 °C; ^{1}H NMR (400 MHz, $CDCl_3$) δ 7.28-7.27 (m, 2H, ArH), 7.21 (d, J = 8.4 Hz, 2H, ArH), 3.99 (s, 3H, OCH_3), 3.74 (s, 3H, OCH_3), 3.52 (s, 1H, OH), 3.24 (d, J = 14.8 Hz, 1H, CH_2), 2.96 (d, J = 14.8 Hz, 1H, CH_2), 2.36 (s, 3H, CH_3). ^{13}C NMR (100 MHz, $CDCl_3$) δ 163.7, 159.7, 152.8, 139.1, 136.8, 129.7, 124.6, 113.6, 113.3, 86.9, 53.7, 53.3, 52.4, 38.0, 21.1. MS (*m/z*): HRMS (ESI) Calcd. for $C_{18}H_{16}NaN_2O_5$ ([M+Na]$^+$): 363.0957, found: 363.0957.

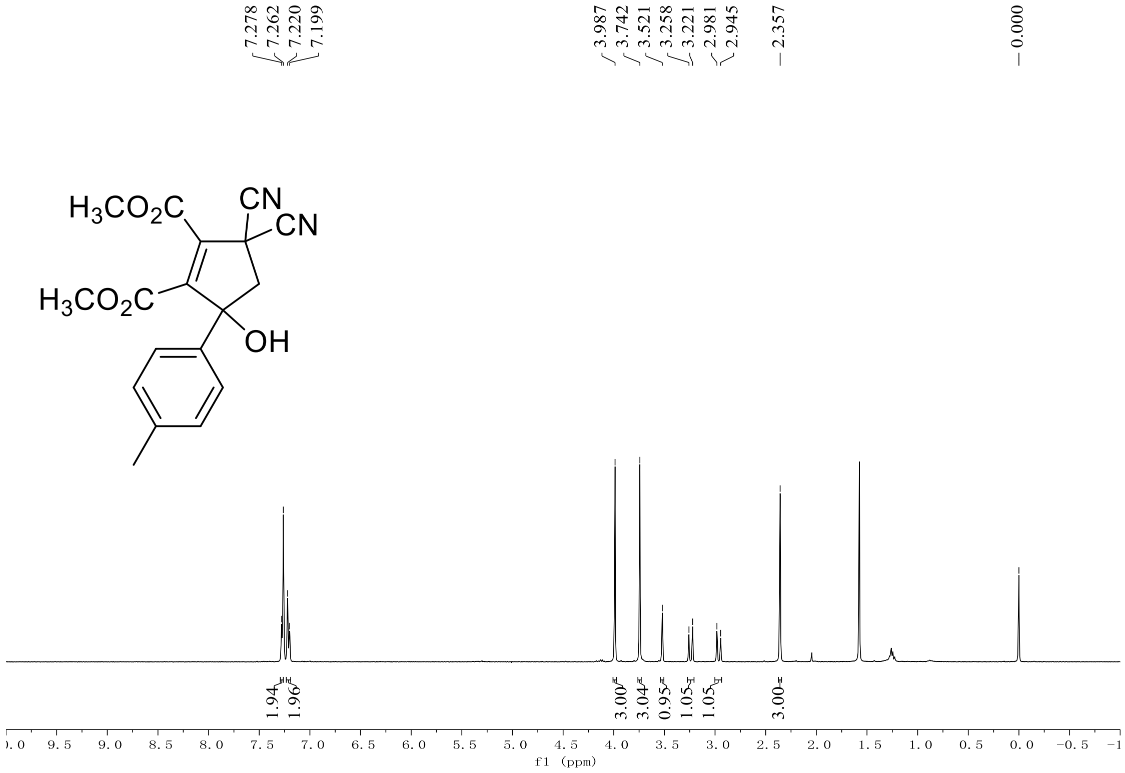
7.278
7.262
7.220
7.199
3.987
3.742
3.521
3.258
3.221
2.981
2.945
2.357
0.000
H3CO2C
CN
CN
H3CO2C
OH
1.94
1.96
3.00
3.04
0.95
1.05
1.05
3.00
f1 (ppm)

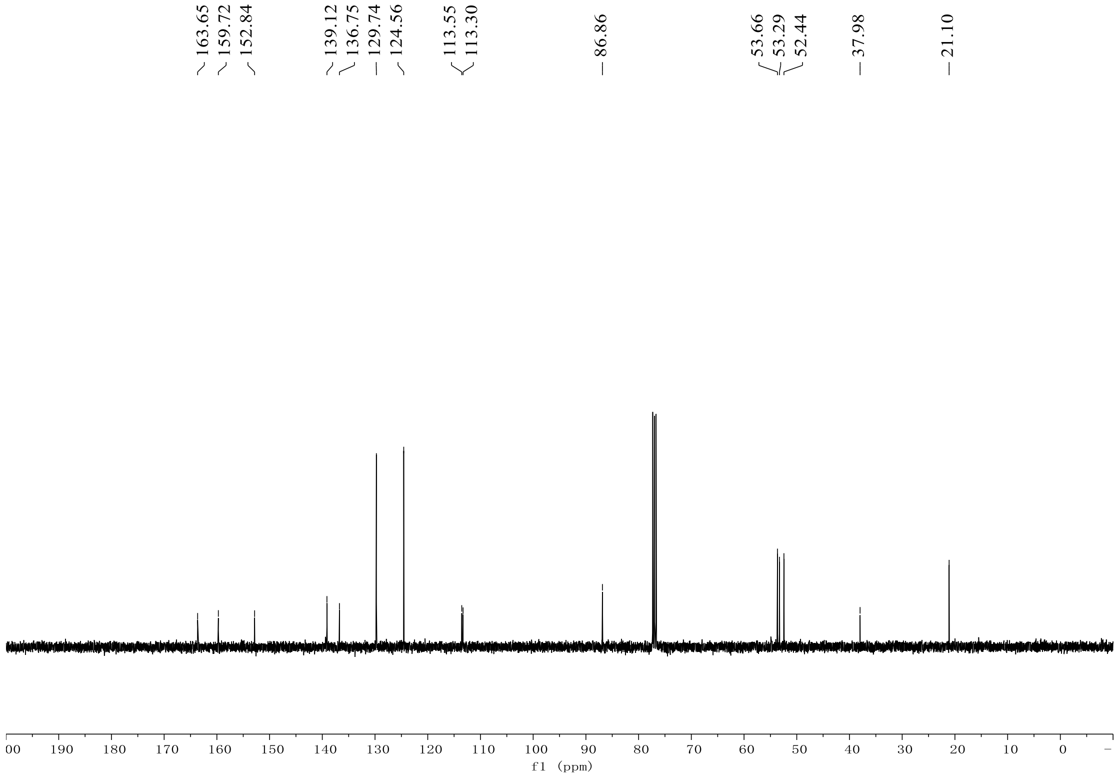
163.65
159.72
152.84
139.12
136.75
129.74
124.56
113.55
113.30
86.86
53.66
53.29
52.44
37.98
21.10
f1 (ppm)

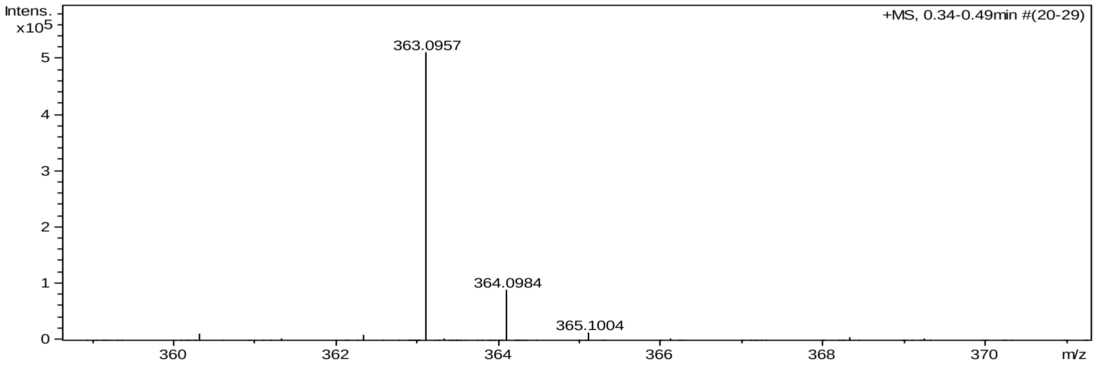

Dimethyl 3,3-dicyano-5-hydroxy-5-(4-methoxyphenyl)cyclopent-1-ene-1,2-dicarboxylate (3j): white solid, 80%, m.p. 150-152 °C; ^{1}H NMR (400 MHz, $CDCl_3$) δ 7.31 (d, *J* = 8.8 Hz, 2H, ArH), 6.92 (d, *J* = 8.8 Hz, 2H, ArH), 3.98 (s, 3H, OCH_3), 3.82 (s, 3H, OCH_3), 3.75 (s, 3H, OCH_3), 3.49 (s, 1H, OH), 3.23 (d, *J* = 14.4 Hz, 1H, CH_2), 2.96 (d, *J* = 14.8 Hz, 1H, CH_2). ^{13}C NMR (100 MHz, $CDCl_3$) δ 163.7, 160.0, 159.7, 153.1, 131.6, 129.3, 126.1, 114.4, 113.6, 113.3, 86.7, 55.3, 53.7, 53.3, 52.5, 37.9. MS (*m/z*): HRMS (ESI) Calcd. for $C_{18}H_{16}NaN_2O_6$ ($[M+Na]^+$): 379.0906, found: 379.0909.

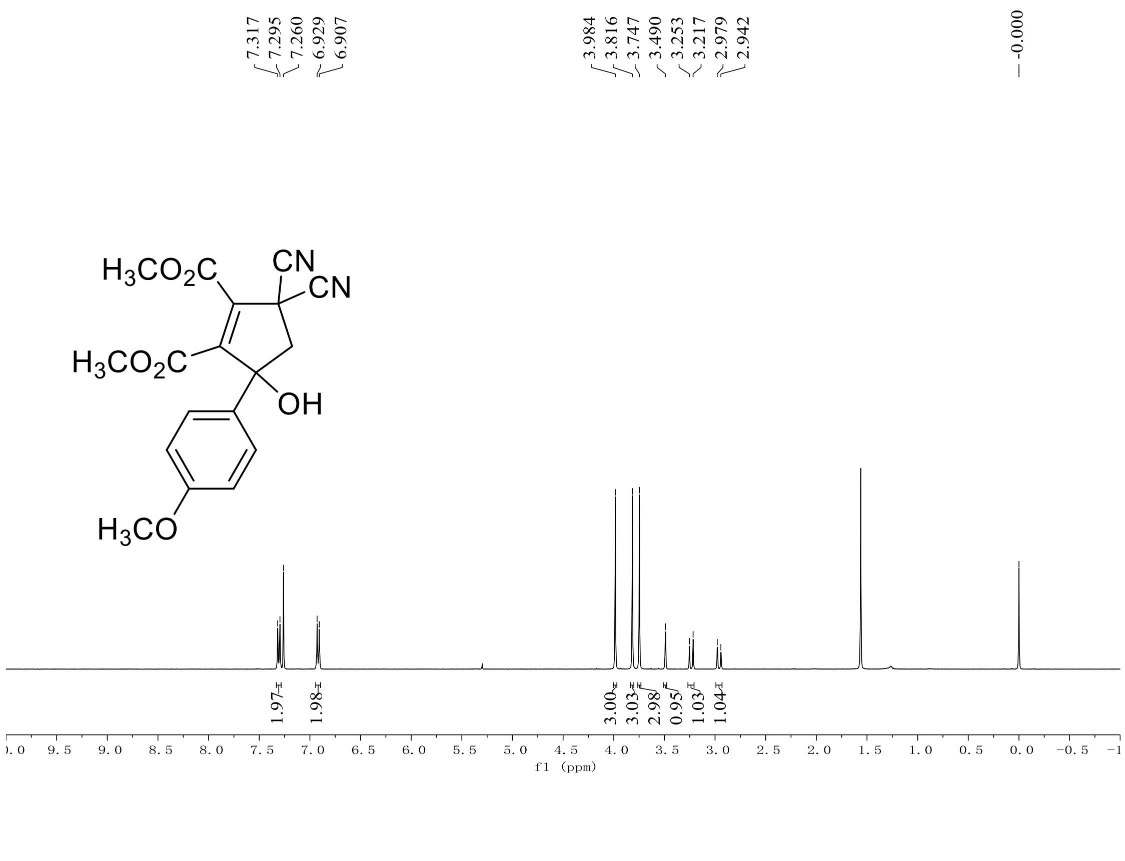

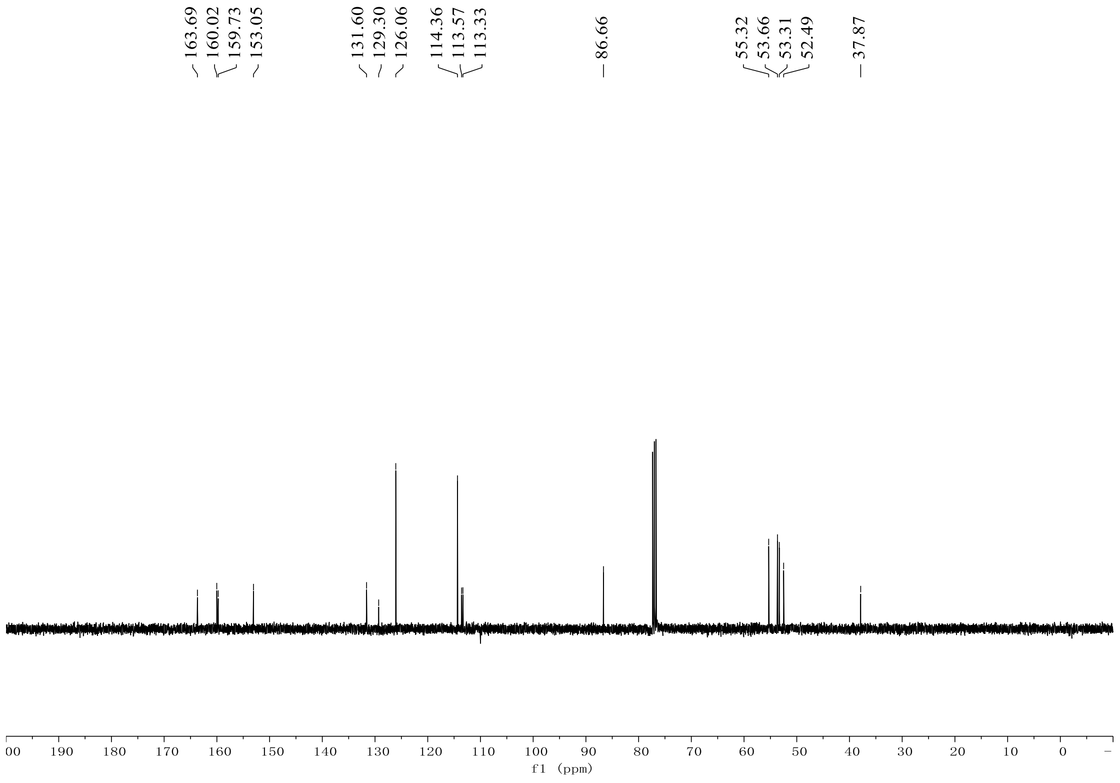

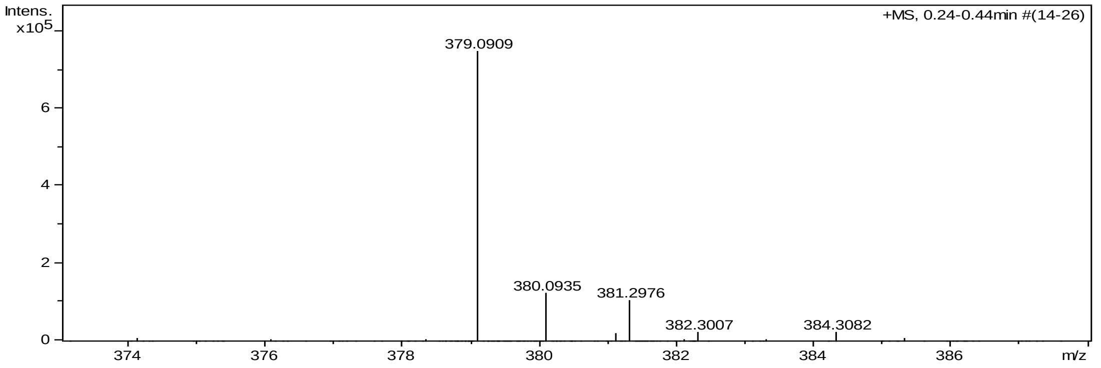

Dimethyl 3-(4-chlorophenyl)-5,5-dicyano-3-hydroxycyclopent-1-ene-1,2-dicarboxylate (3k): white solid, 52%, m.p. 176-178 °C; ^{1}H NMR (400 MHz, $CDCl_3$) δ 7.41-7.33 (m, 4H, ArH), 4.00 (s, 3H, OCH_3), 3.76 (s, 3H, OCH_3), 3.67 (s, 1H, OH), 3.26 (d, J = 14.4 Hz, 1H, CH_2), 2.94 (d, J = 14.4 Hz, 1H, CH_2). ^{13}C NMR (100 MHz, $CDCl_3$) δ 163.5, 159.6, 152.0, 138.3, 135.3, 130.1, 129.3, 126.2, 113.4, 113.1, 86.5, 53.8, 53.5, 52.4, 38.0. MS (*m*/*z*): HRMS (ESI) Calcd. for $C_{17}H_{13}ClNaN_2O_5$ ([M+Na]$^+$): 383.0411, found: 383.0409.

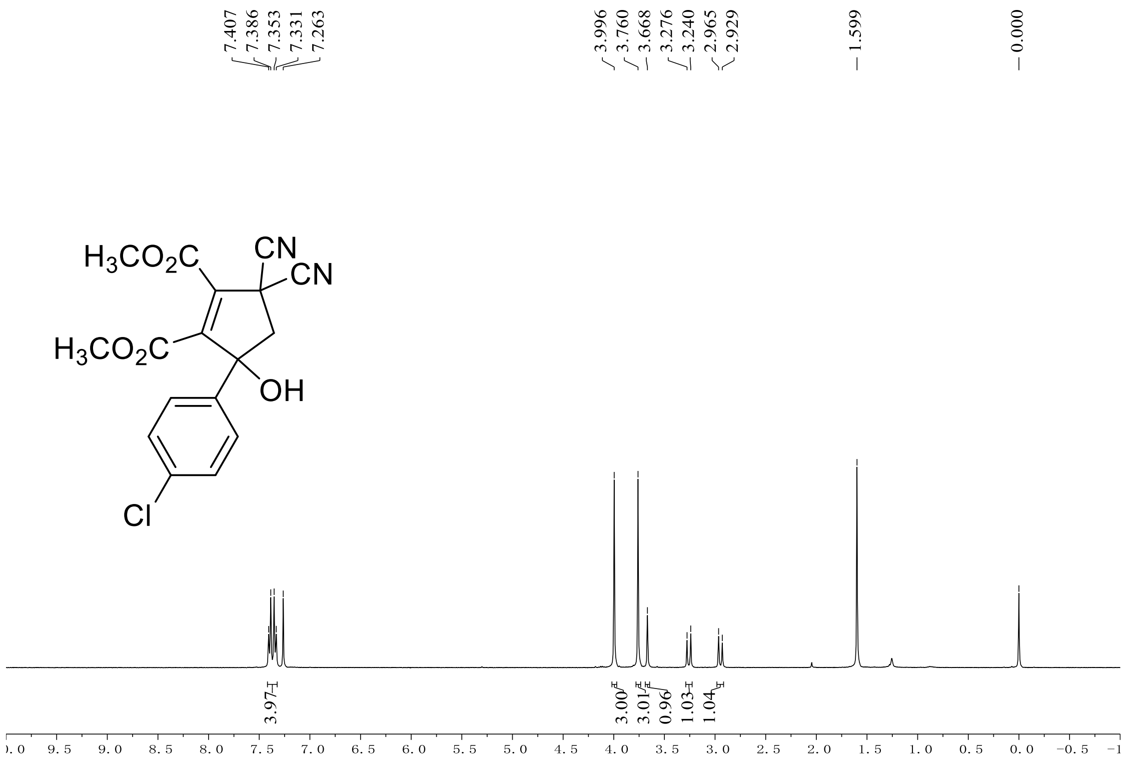
7.407
7.386
7.353
7.331
7.263
3.996
3.760
3.668
3.276
3.240
2.965
2.929
1.599
0.000
H3CO2C
CN
CN
H3CO2C
OH
Cl
3.97
3.00
3.01
0.96
1.03
1.04
f1 (ppm)

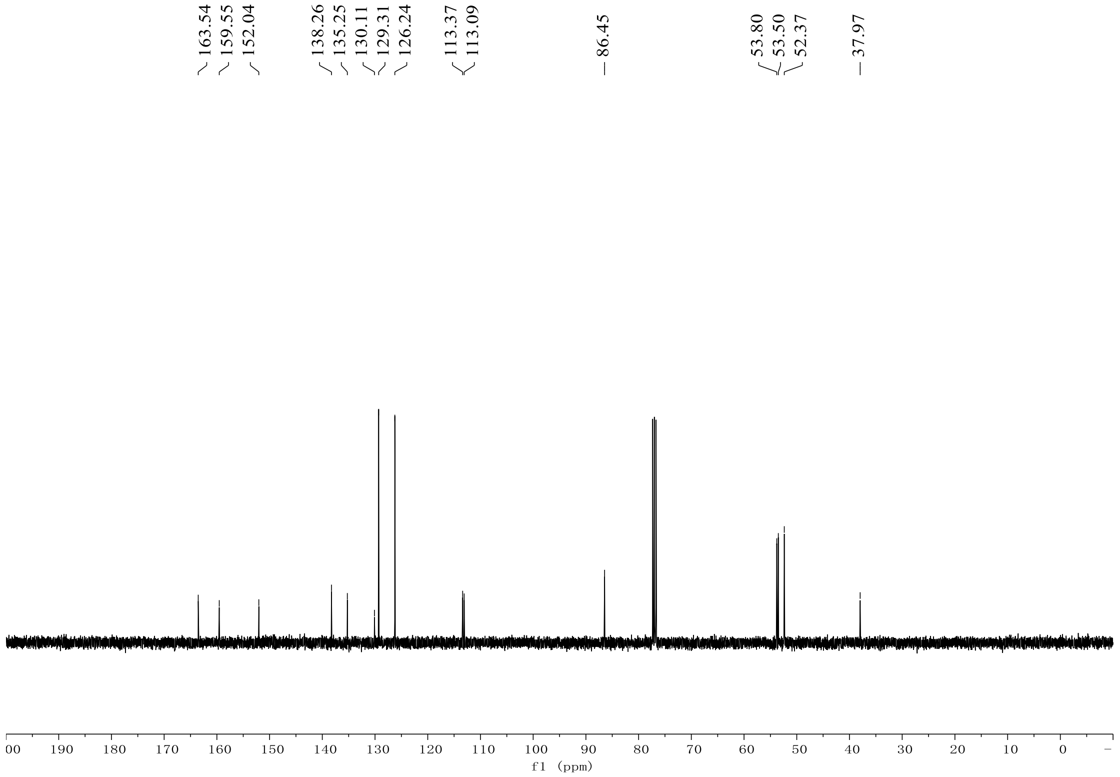
163.54
159.55
152.04
138.26
135.25
130.11
129.31
126.24
113.37
113.09
86.45
53.80
53.50
52.37
37.97
f1 (ppm)

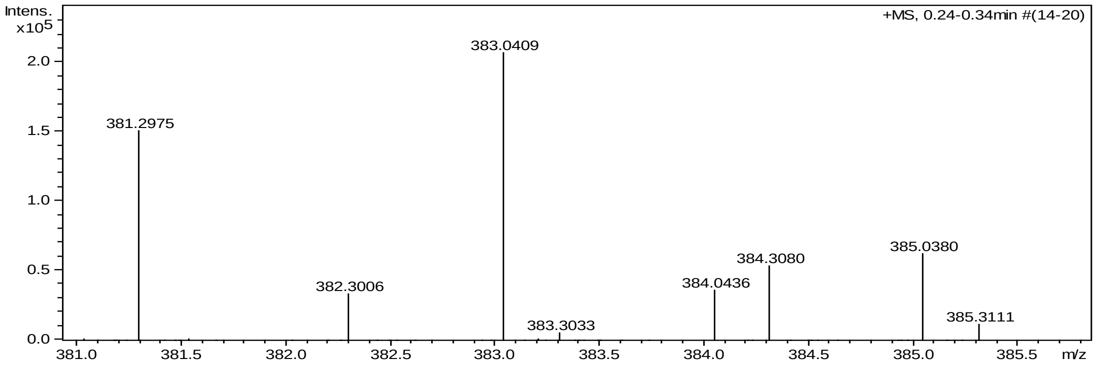

Dimethyl 3-(4-bromophenyl)-5,5-dicyano-3-hydroxycyclopent-1-ene-1,2-dicarboxylate (3l): white solid, 55%, m.p. 189-191 °C; ^{1}H NMR (400 MHz, $CDCl_3$) δ 7.55 (d, *J* = 8.4 Hz, 2H, ArH), 7.28 (d, *J* = 8.8 Hz, 2H, ArH), 4.00 (s, 3H, OCH_3), 3.76 (s, 3H, OCH_3), 3.64 (s, 1H, OH), 3.26 (d, *J* = 14.8 Hz, 1H, CH_2), 2.94 (d, *J* = 14.8 Hz, 1H, CH_2). ^{13}C NMR (100 MHz, $CDCl_3$) δ 163.5, 159.5, 152.0, 138.8, 132.3, 130.2, 126.5, 123.4, 113.4, 113.1, 86.5, 53.8, 53.5, 52.3, 38.0. MS (*m/z*): HRMS (ESI) Calcd. for $C_{17}H_{13}BrNaN_2O_5$ ($[M+Na]^+$): 426.9906, found: 426.9901.

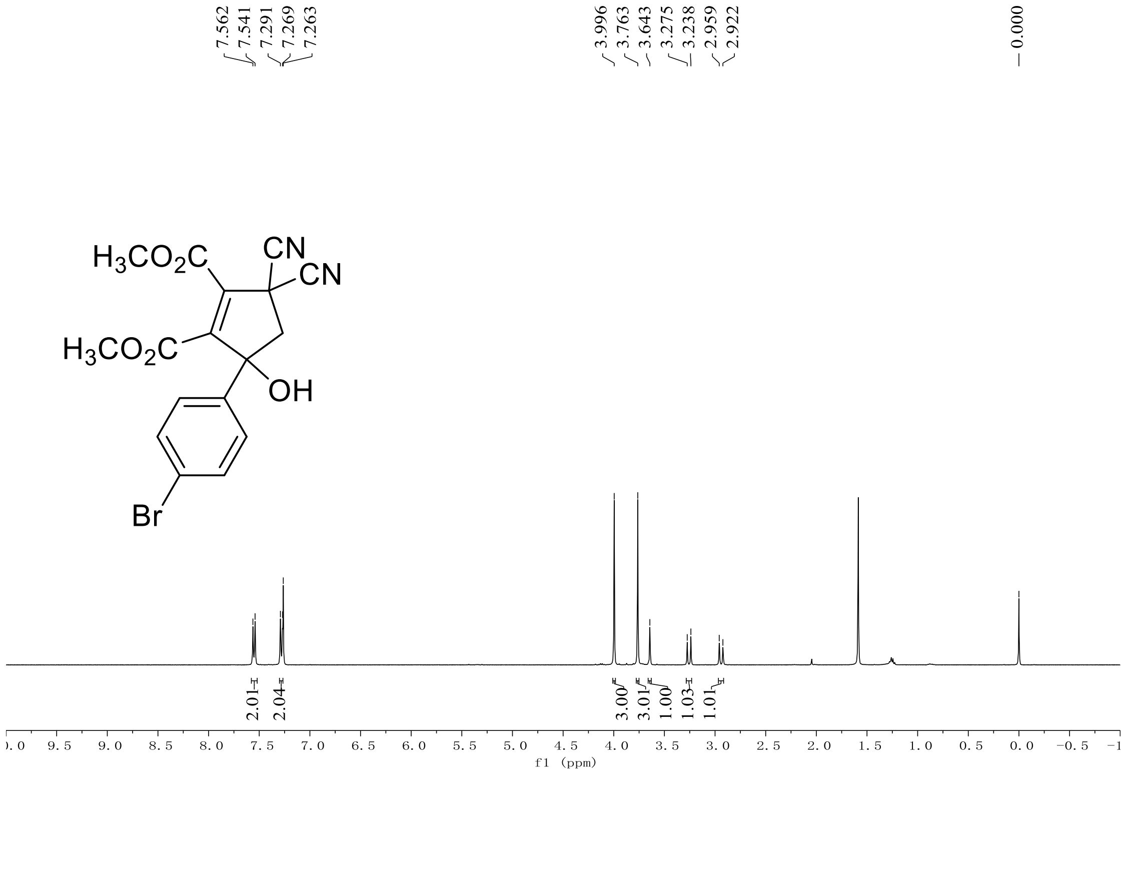

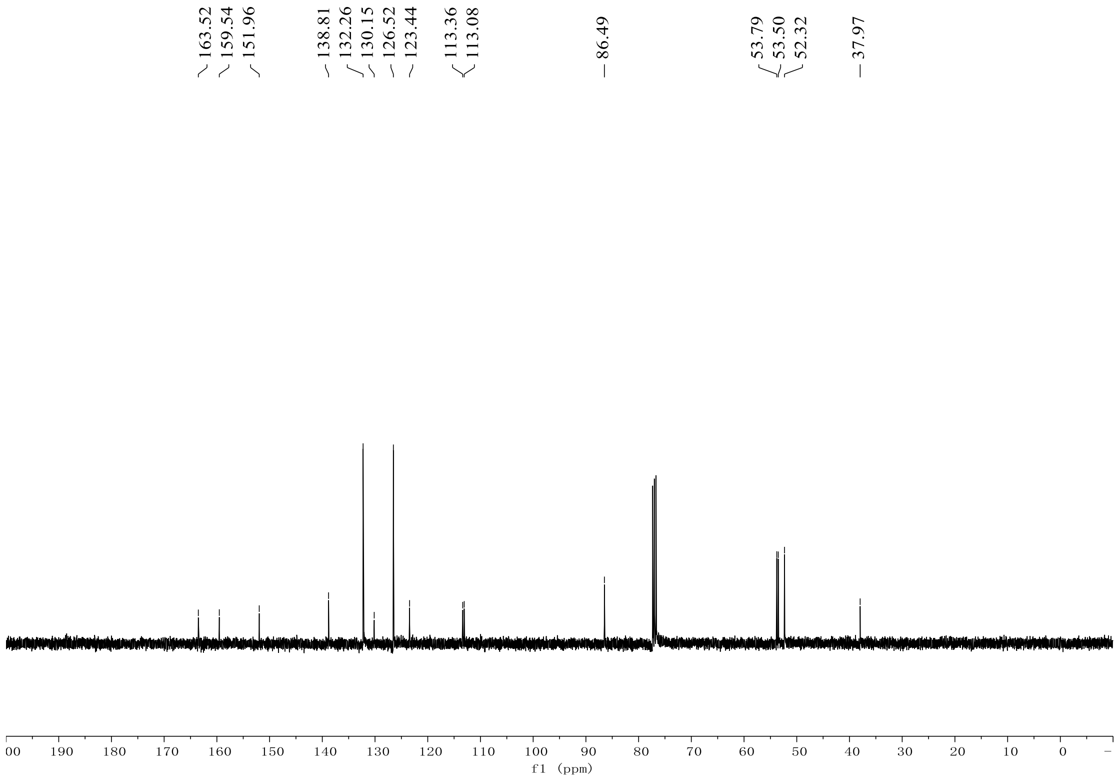

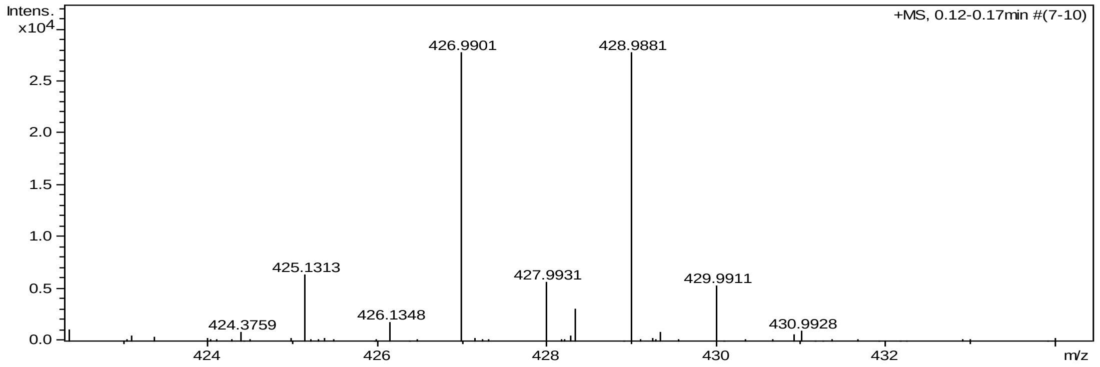

Diethyl 3-cyano-1-(1-cyano-2,3-bis(ethoxycarbonyl)-4-hydroxy-4-(4-methoxyphenyl)cyclo-pent-2-ene-1-carboxamido)-5-(4-methoxyphenyl)cyclopenta-2,4-diene-1,2-dicarboxylate (4a): For characterization data set, see main text.

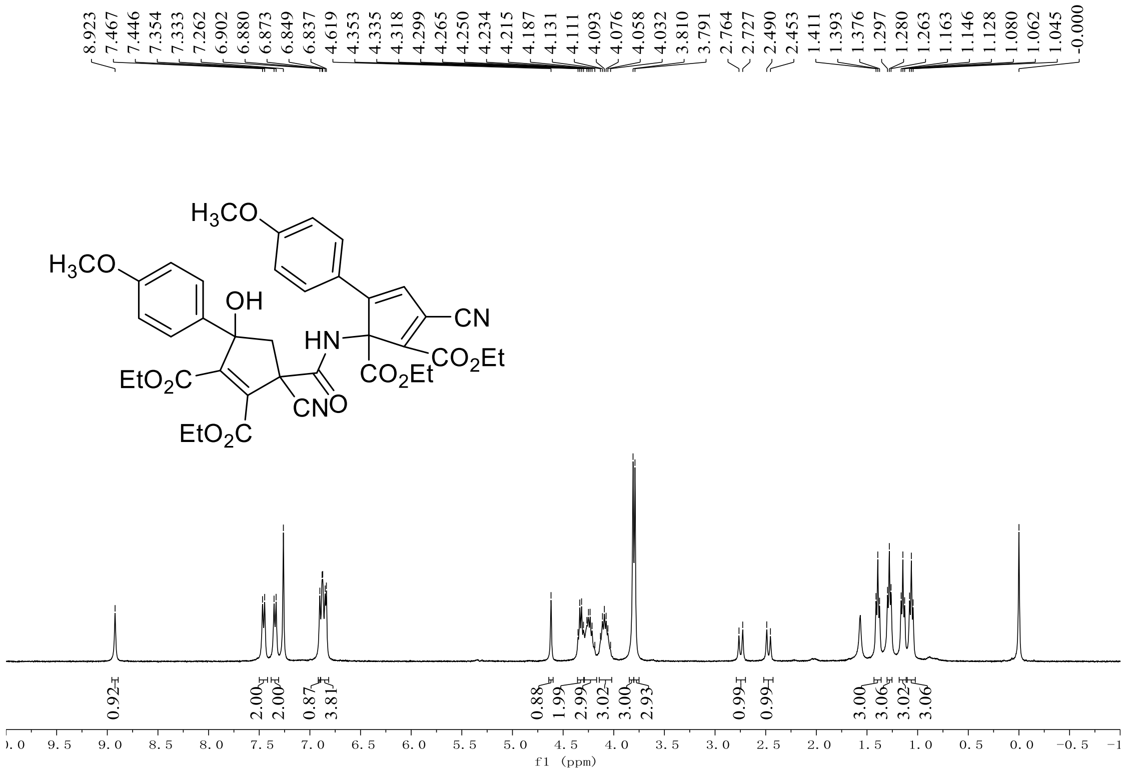
8.923 7.467 7.446 7.354 7.333 7.262 6.902 6.880 6.873 6.849 6.837 4.619 4.353 4.335 4.318 4.299 4.265 4.250 4.234 4.215 4.187 4.131 4.111 4.093 4.076 4.058 4.032 3.810 3.791 2.764 2.727 2.490 2.453 1.411 1.393 1.376 1.297 1.280 1.263 1.163 1.146 1.128 1.080 1.062 1.045 -0.000
H_3CO
H_3CO
OH
HN
CN
EtO_2C
CO_2Et
CO_2Et
CN
O
EtO_2C
0.92 2.00 2.00 0.87 3.81 0.88 1.99 2.99 3.02 3.00 2.93 0.99 0.99 3.00 3.06 3.02 3.06
f1 (ppm)

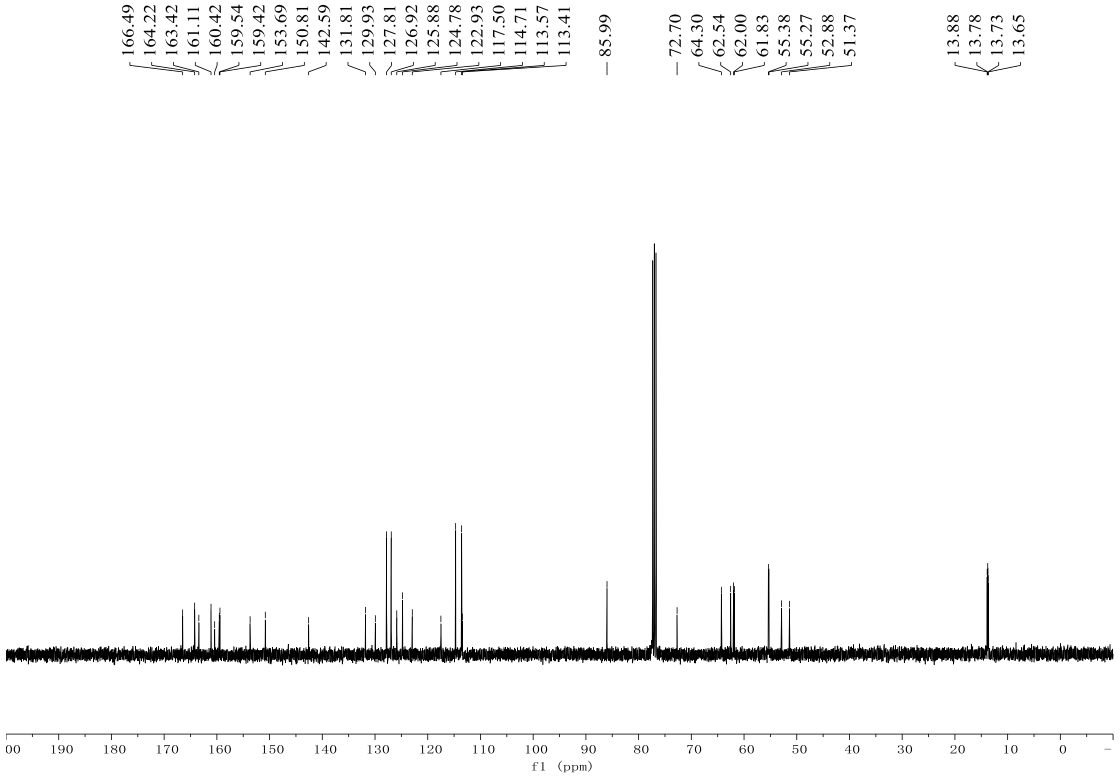
166.49 164.22 163.42 161.11 160.42 159.54 159.42 153.69 150.81 142.59 131.81 129.93 127.81 126.92 125.88 124.78 122.93 117.50 114.71 113.57 113.41 85.99 72.70 64.30 62.54 62.00 61.83 55.38 55.27 52.88 51.37 13.88 13.78 13.73 13.65
f1 (ppm)

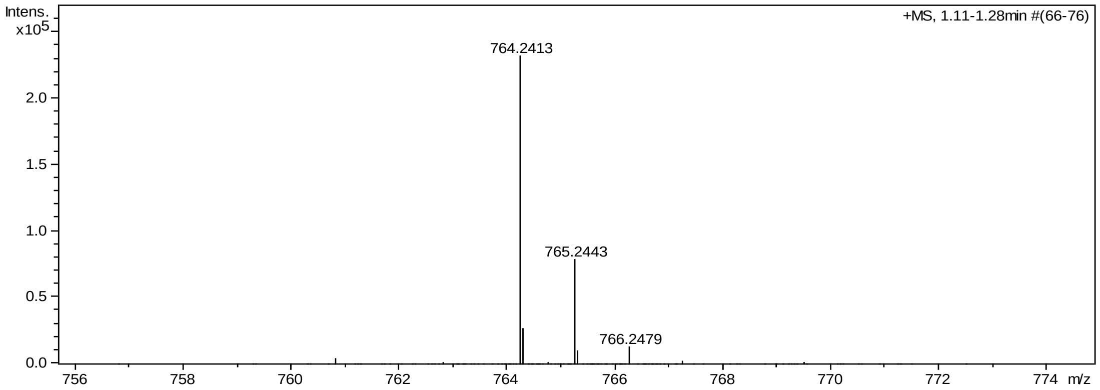

Diethyl 3-cyano-1-(1-cyano-2,3-bis(ethoxycarbonyl)-4-hydroxy-4-(3-methoxyphenyl)cyclo-pent-2-ene-1-carboxamido)-5-(3-methoxyphenyl)cyclopenta-2,4-diene-1,2-dicarboxylate (4b): fluorescent green solid, 40%, m.p. 108-110 °C; ^{1}H NMR (400 MHz, $CDCl_3$) δ 8.94 (s, 1H, NH), 7.30-7.28 (m, 1H, ArH), 7.26-7.23 (m, 1H, ArH), 7.07 (d, *J* = 7.6 Hz, 1H, ArH), 7.01 (d, *J* = 7.6 Hz, 2H, ArH), 6.97 (s, 1H, CH), 6.94-6.90 (m, 2H, ArH), 6.84-6.81 (m, 1H, ArH), 4.65 (s, 1H, OH), 4.37-4.32 (m, 2H, OCH_2), 4.28-4.22 (m, 3H, OCH_2), 4.13-4.01 (m, 3H, OCH_2), 3.81 (s, 3H, OCH_3), 3.80 (s, 3H, OCH_3), 2.77 (d, *J* = 14.8 Hz, 1H, CH_2), 2.50 (d, *J* = 15.2 Hz, 1H, CH_2), 1.40 (t, *J* = 7.2 Hz, 3H, CH_3), 1.26 (t, *J* = 7.2 Hz, 3H, CH_3), 1.16 (t, *J* = 7.2 Hz, 3H, CH_3), 1.06 (t, *J* = 7.2 Hz, 3H, CH_3). ^{13}C NMR (100 MHz, $CDCl_3$) δ 166.3, 164.3, 163.4, 160.3, 160.0, 159.6, 159.4, 153.2, 150.9, 144.1, 141.5, 131.4, 130.6, 130.3, 129.4, 127.3, 125.4, 118.6, 118.0, 117.4, 116.7, 114.1, 113.2, 110.9, 110.8, 86.3, 72.9, 64.4, 62.6, 62.2, 61.9, 55.3, 55.2, 53.2, 51.3, 13.9, 13.7, 13.6. MS (*m/z*): HRMS (ESI) Calcd. for $C_{39}H_{39}NaN_3O_{12}$ ($[M+Na]^+$): 764.2431, found: 764.2419.

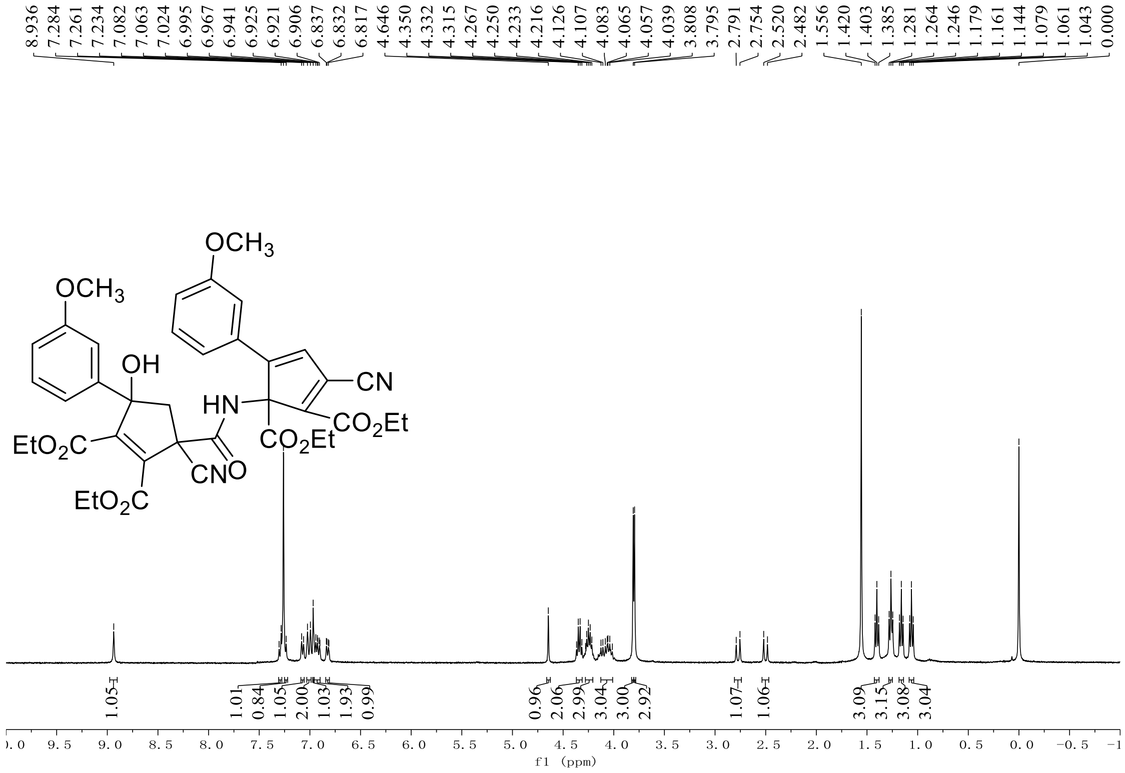
8.936 7.284 7.261 7.234 7.082 7.063 7.024 6.995 6.967 6.941 6.925 6.921 6.906 6.837 6.832 6.817 4.646 4.350 4.332 4.315 4.267 4.250 4.233 4.216 4.126 4.107 4.083 4.065 4.057 4.039 3.808 3.795 2.791 2.754 2.520 2.482 1.556 1.420 1.403 1.385 1.281 1.264 1.246 1.179 1.161 1.144 1.079 1.061 1.043 0.000
OCH3
OCH3
OH
HN
CN
EtO2C
CO2Et
CO2Et
CN
O
EtO2C
1.05 1.01 0.84 1.05 2.00 1.03 1.93 0.99 0.96 2.06 2.99 3.04 3.00 2.92 1.07 1.06 3.09 3.15 3.08 3.04
f1 (ppm)

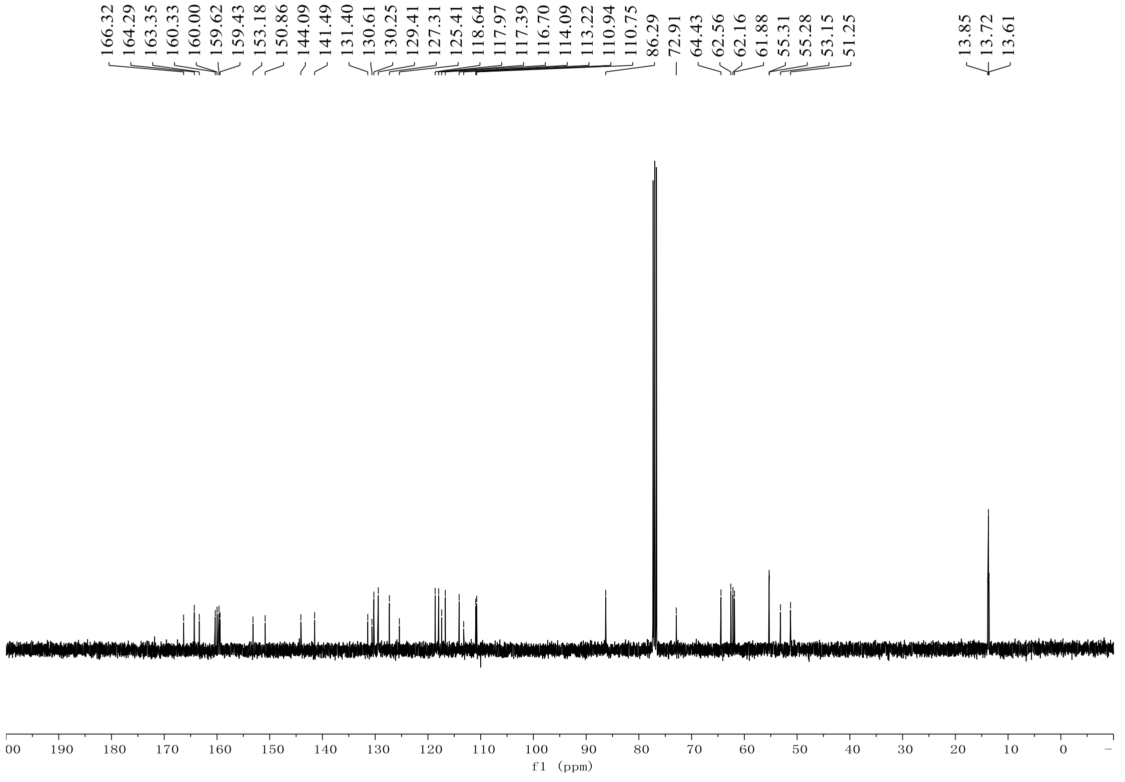
166.32 164.29 163.35 160.33 160.00 159.62 159.43 153.18 150.86 144.09 141.49 131.40 130.61 130.25 129.41 127.31 125.41 118.64 117.97 117.39 116.70 114.09 113.22 110.94 110.75 86.29 72.91 64.43 62.56 62.16 61.88 55.31 55.28 53.15 51.25 13.85 13.72 13.61
f1 (ppm)

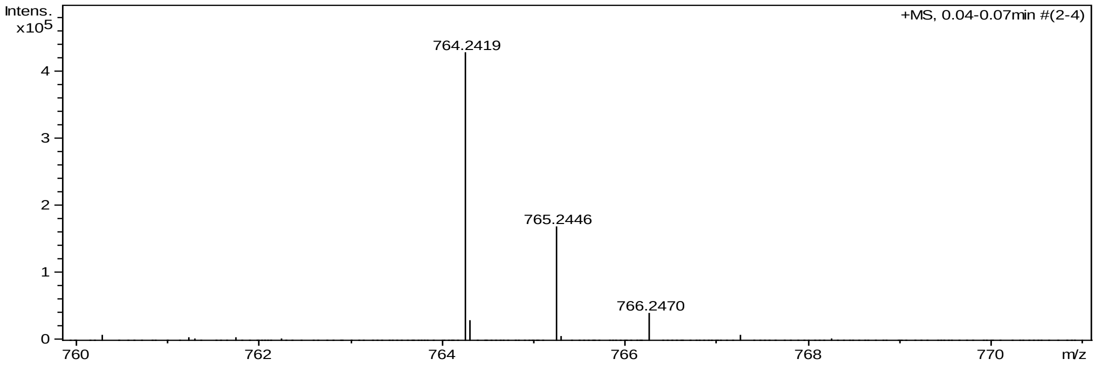

Diethyl 3-cyano-1-(1-cyano-2,3-bis(ethoxycarbonyl)-4-hydroxy-4-(*p*-tolyl)cyclopent-2-ene-1-carboxamido)-5-(*p*-tolyl)cyclopenta-2,4-diene-1,2-dicarboxylate (4c): fluorescent green solid, 60%, m.p. 178-180 °C; ^{1}H NMR (400 MHz, $CDCl_3$) δ 8.91 (s, 1H, NH), 7.38 (d, *J* = 8.4 Hz, 2H, ArH), 7.30 (d, *J* = 8.0 Hz, 2H, ArH), 7.19-7.13 (m, 4H, ArH), 6.92 (s, 1H, CH), 4.58 (s, 1H, OH), 4.36-4.31 (m, 2H, OCH_2), 4.27-4.19 (m, 3H, OCH_2), 4.12-4.03 (m, 3H, OCH_2), 2.75 (d, *J* = 14.8 Hz, 1H, CH_2), 2.44 (d, *J* = 14.8 Hz, 1H, CH_2), 2.33 (s, 3H, CH_3), 2.32 (s, 3H, CH_3), 1.40 (t, *J* = 7.2 Hz, 3H, CH_3), 1.26 (t, *J* = 7.2 Hz, 3H, CH_3), 1.15 (t, *J* = 7.2 Hz, 3H, CH_3), 1.05 (t, *J* = 7.2 Hz, 3H, CH_3). ^{13}C NMR (100 MHz, $CDCl_3$) δ 166.3, 164.3, 163.4, 160.4, 159.5, 153.5, 151.1, 143.4, 140.7, 138.1, 136.8, 130.2, 130.0, 128.9, 127.5, 126.1, 125.7, 125.5, 117.5, 113.3, 86.2, 72.8, 64.3, 62.5, 62.1, 61.8, 53.0, 51.4, 21.4, 21.0, 13.9, 13.7, 13.6. MS (*m/z*): HRMS (ESI) Calcd. for $C_{39}H_{39}NaN_3O_{10}$ ([M+Na]$^+$): 732.2533, found: 732.2530.

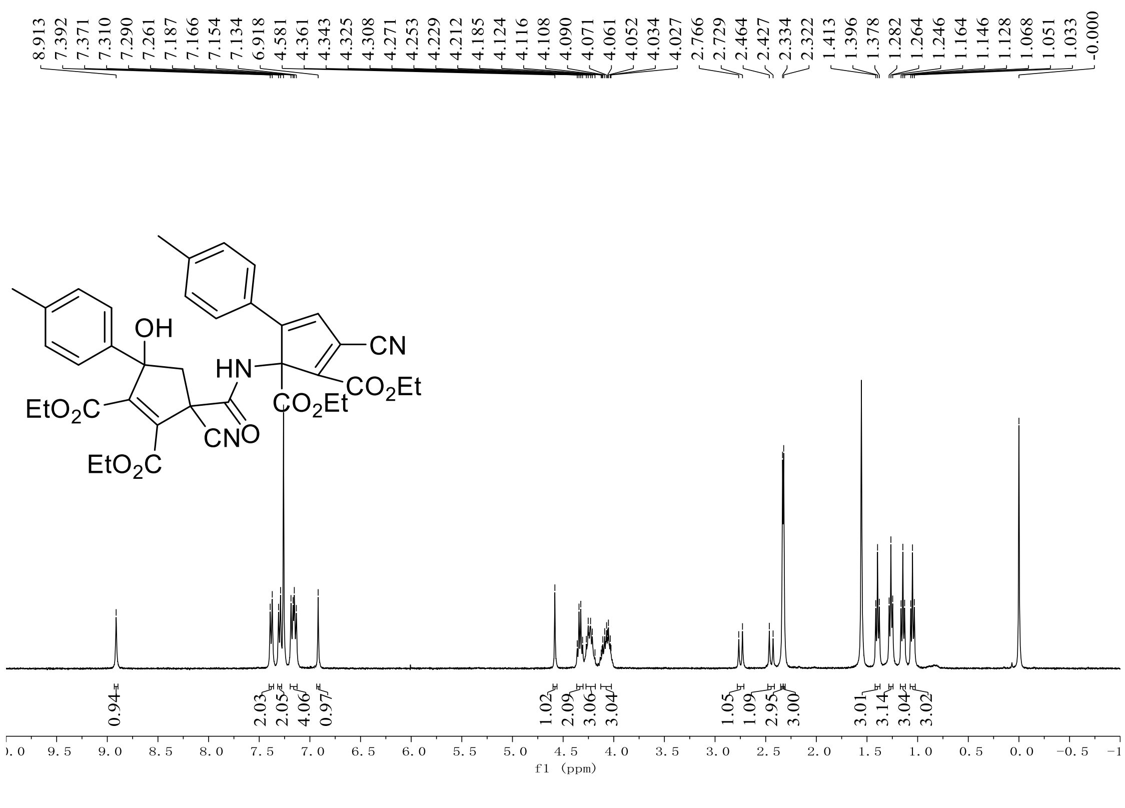

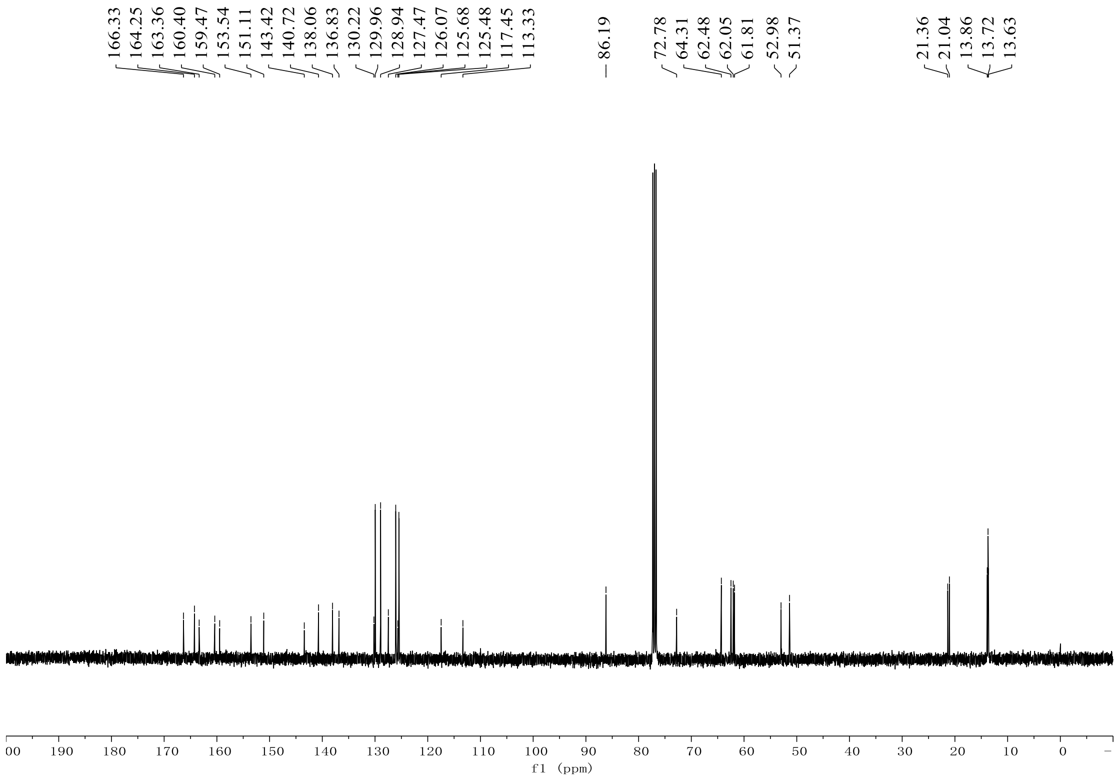

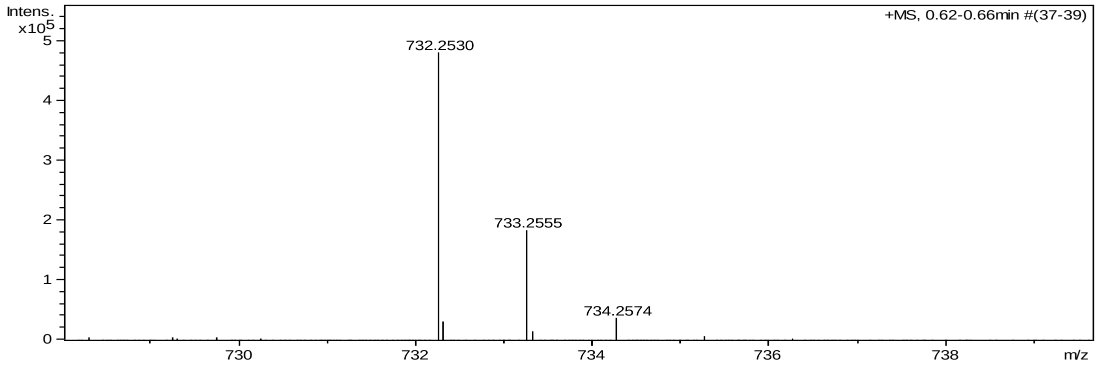

Diethyl 3-cyano-1-(1-cyano-2,3-bis(ethoxycarbonyl)-4-hydroxy-4-phenylcyclopent-2-ene-1-carboxamido)-5-phenylcyclopenta-2,4-diene-1,2-dicarboxylate (4d): fluorescent green solid, 56%, m.p. 169-171 °C; ^{1}H NMR (400 MHz, $CDCl_3$) δ 8.93 (s, 1H, NH), 7.49-7.47 (m, 2H, ArH), 7.43-7.33 (m, 7H, ArH), 7.29 (d, *J* = 6.8 Hz, 1H, ArH), 6.98 (s, 1H, CH), 4.61 (s, 1H, OH), 4.37-4.32 (m, 2H, OCH_2), 4.27-4.22 (m, 3H, OCH_2), 4.13-4.02 (m, 3H, OCH_2), 2.76 (d, *J* = 14.4 Hz, 1H, CH_2), 2.43 (d, *J* = 14.4 Hz, 1H, CH_2), 1.41 (t, *J* = 7.2 Hz, 3H, CH_3), 1.26 (t, *J* = 7.2 Hz, 3H, CH_3), 1.16 (t, *J* = 7.2 Hz, 3H, CH_3), 1.02 (t, *J* = 7.2 Hz, 3H, CH_3). ^{13}C NMR (100 MHz, $CDCl_3$) δ 166.2, 164.3, 163.3, 160.4, 159.4, 153.3, 151.0, 144.0, 139.8, 130.5, 130.2, 129.3, 128.3, 127.0, 126.2, 125.6, 117.4, 113.3, 86.3, 72.9, 64.4, 62.6, 62.2, 61.8, 53.1, 51.3, 13.9, 13.8, 13.7, 13.6. MS (*m*/*z*): HRMS (ESI) Calcd. for $C_{39}H_{39}NaN_3O_{12}$ ($[M+Na]^+$): 704.2220, found: 704.2211.

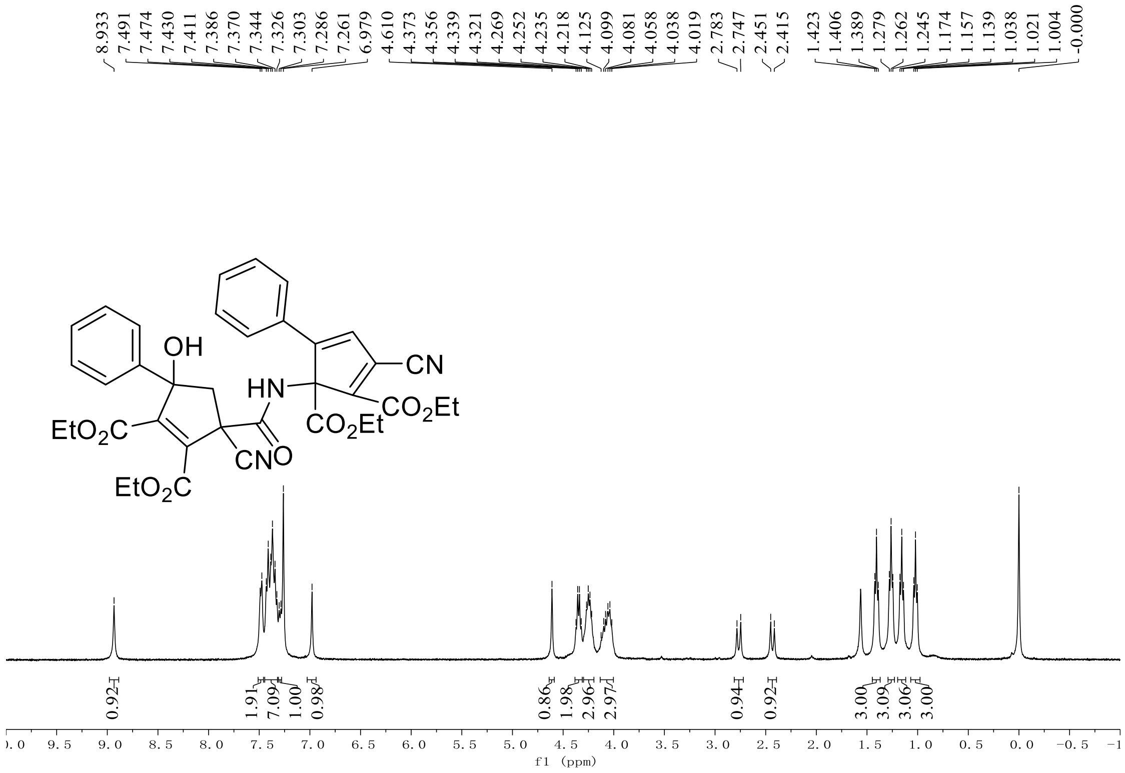

8.933
7.491
7.474
7.430
7.411
7.386
7.370
7.344
7.326
7.303
7.286
7.261
6.979
4.610
4.373
4.356
4.339
4.321
4.269
4.252
4.235
4.218
4.125
4.099
4.081
4.058
4.038
4.019
2.783
2.747
2.451
2.415
1.423
1.406
1.389
1.279
1.262
1.245
1.174
1.157
1.139
1.038
1.021
1.004
-0.000
OH
HN
CN
EtO2C
CO2Et
CO2Et
CN
O
EtO2C
0.92
1.91
7.09
1.00
0.98
0.86
1.98
2.96
2.97
0.94
0.92
3.00
3.09
3.06
3.00
f1 (ppm)

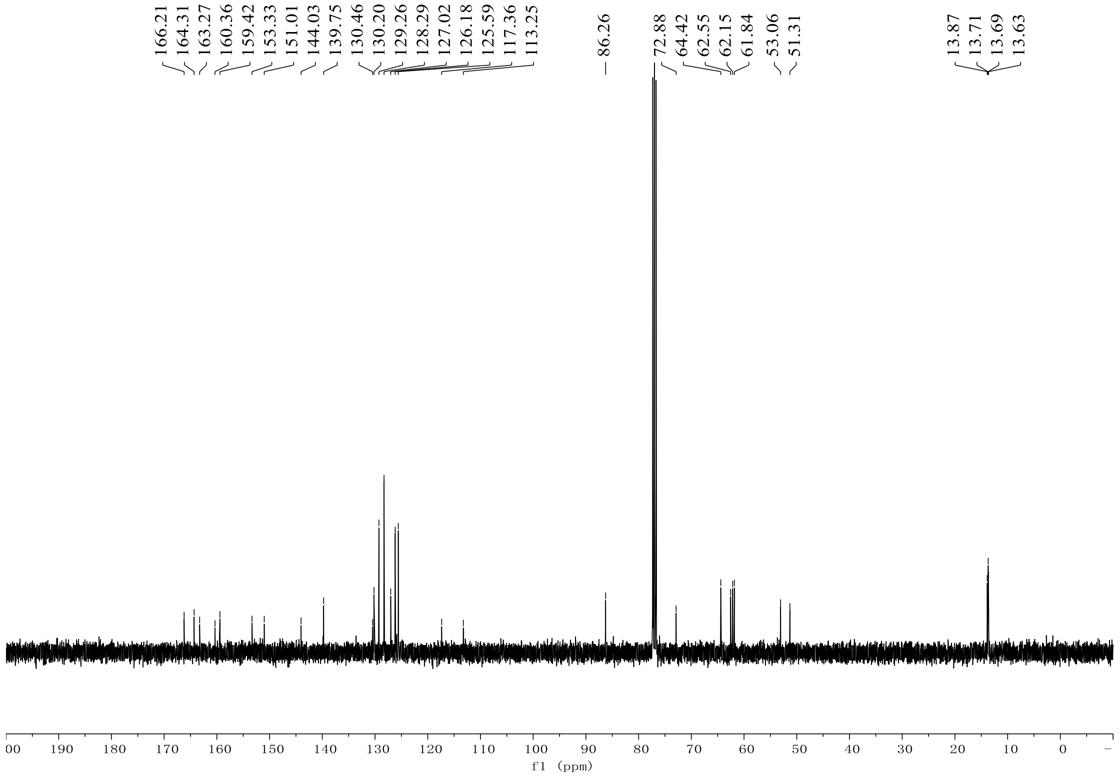

166.21
164.31
163.27
160.36
159.42
153.33
151.01
144.03
139.75
130.46
130.20
129.26
128.29
127.02
126.18
125.59
117.36
113.25
86.26
72.88
64.42
62.55
62.15
61.84
53.06
51.31
13.87
13.71
13.69
13.63
f1 (ppm)

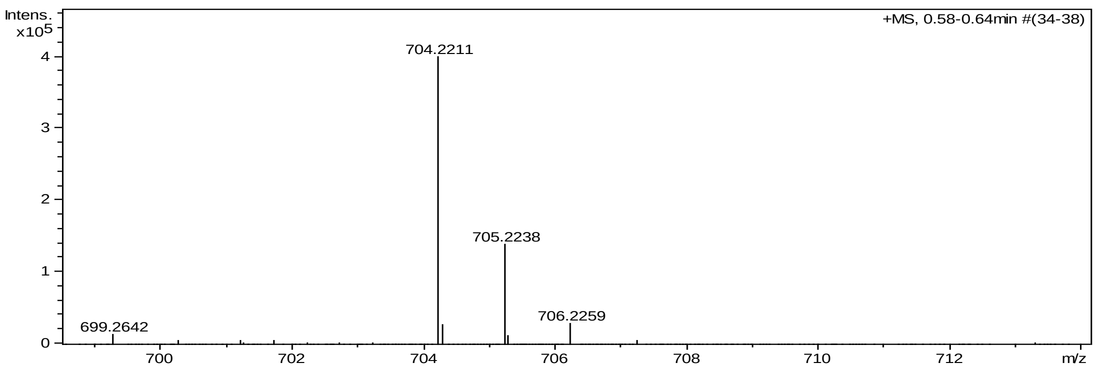

Diethyl 3-cyano-1-(1-cyano-2,3-bis(ethoxycarbonyl)-4-(4-fluorophenyl)-4-hydroxycyclopent-2-ene-1-carboxamido)-5-(4-fluorophenyl)cyclopenta-2,4-diene-1,2-dicarboxylate (4e): pale green solid, 35%, m.p. 198-200 °C; ^{1}H NMR (400 MHz, $CDCl_3$) δ 8.89 (s, 1H, NH), 7.51-7.48 (m, 2H, ArH), 7.43-7.39 (m, 2H, ArH), 7.09 (t, *J* = 8.4 Hz, 2H, ArH), 7.03 (t, *J* = 8.4 Hz, 2H, ArH), 6.91 (s, 1H, CH), 4.68 (s, 1H, OH), 4.37-4.32 (m, 2H, OCH_2), 4.29-4.21 (m, 3H, OCH_2), 4.19-4.03 (m, 3H, OCH_2), 2.73 (d, *J* = 14.8 Hz, 1H, CH_2), 2.45 (d, *J* = 14.8 Hz, 1H, CH_2), 1.40 (t, *J* = 7.2 Hz, 3H, CH_3), 1.30 (t, *J* = 7.2 Hz, 3H, CH_3), 1.16 (t, *J* = 7.2 Hz, 3H, CH_3), 1.06 (t, *J* = 7.2 Hz, 3H, CH_3). ^{13}C NMR (100 MHz, $CDCl_3$) δ 166.1, 164.9, 164.4, 163.8, 163.1, 162.3, 161.3, 160.4, 159.4, 153.1, 149.7, 143.9, 135.5, 130.3, 128.3, 128.2, 127.8, 127.6, 126.8, 126.5, 125.4, 117.2, 116.7, 116.4, 115.3, 115.1, 113.1, 85.8, 72.9, 64.6, 62.7, 62.2, 61.9, 52.7, 51.4, 13.8, 13.7, 13.6. MS (*m*/*z*): HRMS (ESI) Calcd. for $C_{37}H_{33}F_2NaN_3O_{10}$ ([M+Na]$^+$): 740.2032, found: 740.2024.

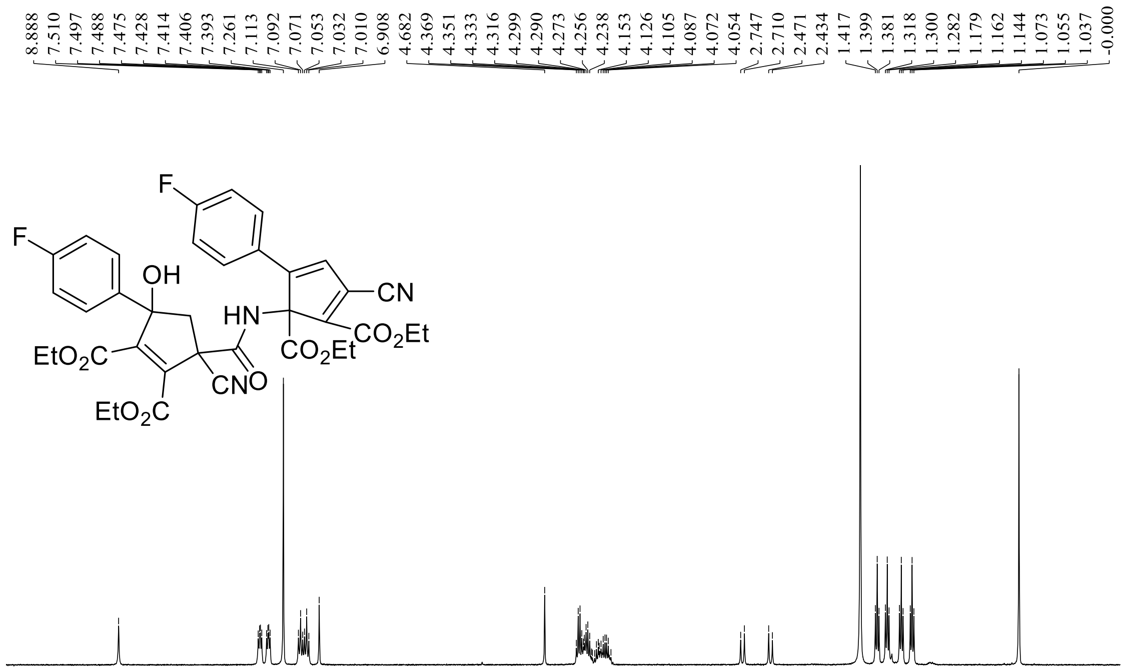

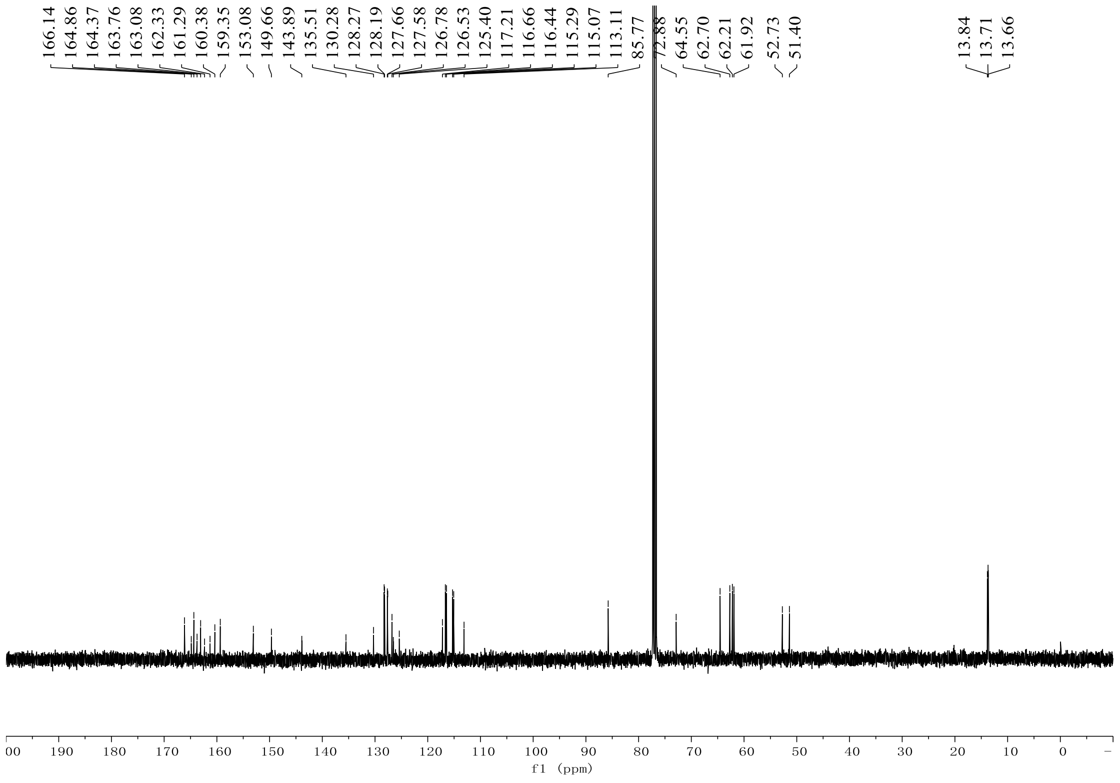

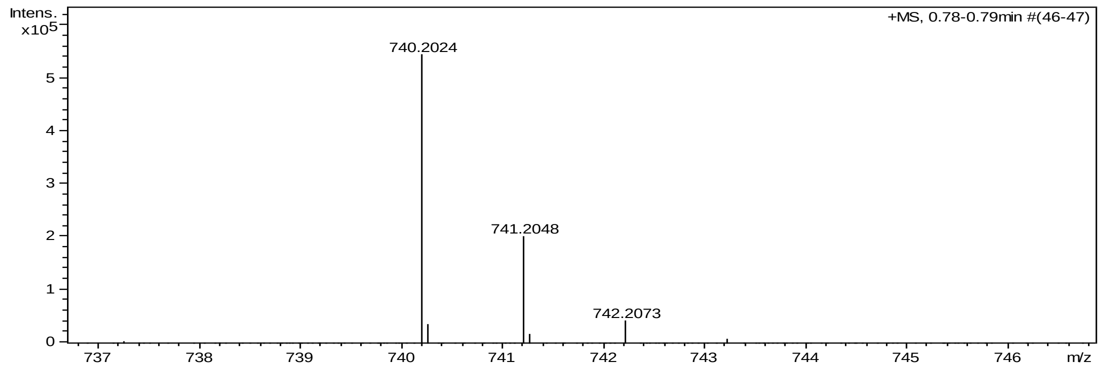

Diethyl 5-(4-chlorophenyl)-1-(4-(4-chlorophenyl)-1-cyano-2,3-bis(ethoxycarbonyl)-4-hydroxycyclopent-2-ene-1-carboxamido)-3-cyanocyclopenta-2,4-diene-1,2-dicarboxylate (4f): pale green solid, 42%, m.p. 197-199 °C; ^{1}H NMR (400 MHz, $CDCl_3$) δ 8.90 (s, 1H, NH), 7.45-7.42 (m, 2H, ArH), 7.37 (d, *J* = 6.4 Hz, 4H, ArH), 7.33-7.31 (m, 2H, ArH), 6.97 (s, 1H, CH), 4.67 (s, 1H, OH), 4.37-4.32 (m, 2H, OCH_2), 4.30-4.22 (m, 3H, OCH_2), 4.17-4.06 (m, 3H, OCH_2), 2.72 (d, *J* = 14.8 Hz, 1H, CH_2), 2.48 (d, *J* = 14.8 Hz, 1H, CH_2), 1.40 (t, *J* = 7.2 Hz, 3H, CH_3), 1.30 (t, *J* = 7.2 Hz, 3H, CH_3), 1.16 (t, *J* = 7.2 Hz, 3H, CH_3), 1.07 (t, *J* = 7.2 Hz, 3H, CH_3). ^{13}C NMR (100 MHz, $CDCl_3$) δ 166.1, 164.3, 163.0, 160.4, 159.3, 152.8, 149.5, 144.2, 138.3, 136.4, 134.3, 130.6, 129.6, 128.6, 128.5, 127.4, 127.3, 127.2, 125.3, 117.1, 113.0, 110.0, 85.8, 72.8, 64.6, 62.8, 62.3, 62.0, 52.8, 51.3, 13.9, 13.8, 13.7, 13.6. MS (*m*/*z*): HRMS (ESI) Calcd. for $C_{37}H_{33}NaN_3O_{10}$ ([M+Na]$^+$): 772.1441, found: 772.1428.

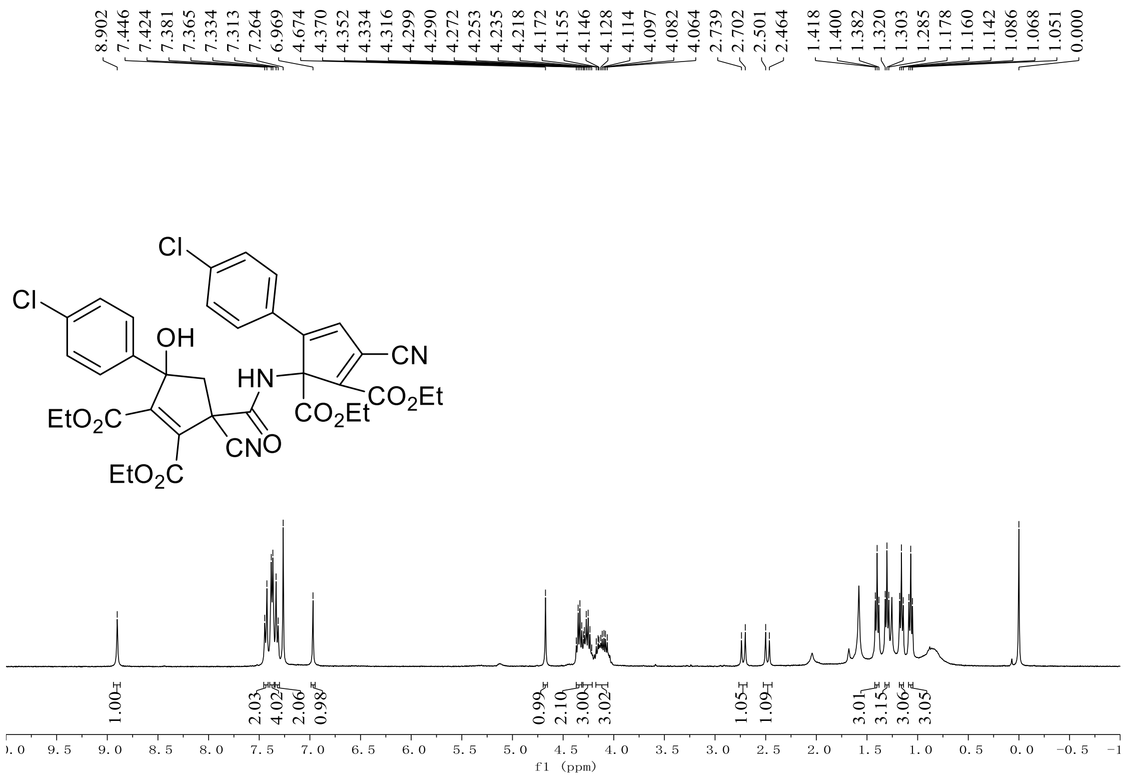
8.902
7.446
7.424
7.381
7.365
7.334
7.313
7.264
6.969
4.674
4.370
4.352
4.334
4.316
4.299
4.290
4.272
4.253
4.235
4.218
4.172
4.155
4.146
4.128
4.114
4.097
4.082
4.064
2.739
2.702
2.501
2.464
1.418
1.400
1.382
1.320
1.303
1.285
1.178
1.160
1.142
1.086
1.068
1.051
0.000
Cl
Cl
OH
HN
CN
CO_2Et
EtO_2C
CO_2Et
CN
O
EtO_2C
1.00
2.03
4.02
2.06
0.98
0.99
2.10
3.00
3.02
1.05
1.09
3.01
3.15
3.06
3.05
f1 (ppm)

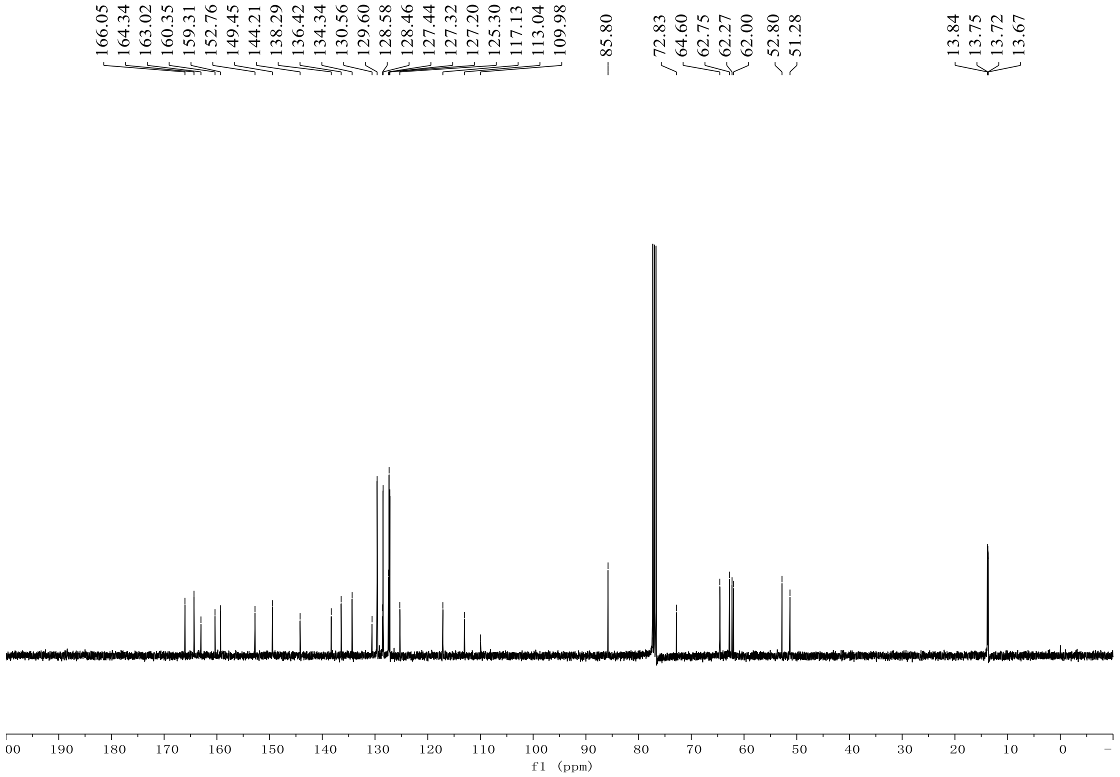
166.05
164.34
163.02
160.35
159.31
152.76
149.45
144.21
138.29
136.42
134.34
130.56
129.60
128.58
128.46
127.44
127.32
127.20
125.30
117.13
113.04
109.98
85.80
72.83
64.60
62.75
62.27
62.00
52.80
51.28
13.84
13.75
13.72
13.67
f1 (ppm)

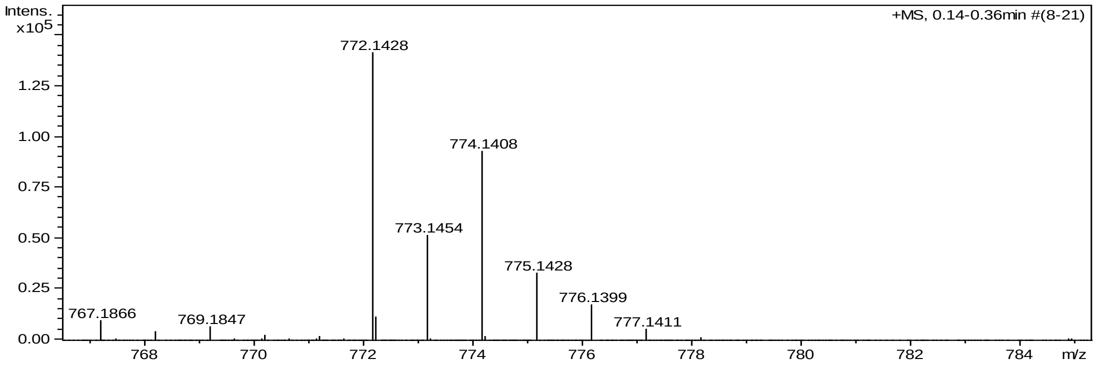

Diethyl 5-(4-bromophenyl)-1-(4-(4-bromophenyl)-1-cyano-2,3-bis(ethoxycarbonyl)-4-hydroxycyclopent-2-ene-1-carboxamido)-3-cyanocyclopenta-2,4-diene-1,2-dicarboxylate (4g): pale green solid, 46%, m.p. 199-201 °C; ^{1}H NMR (400 MHz, $CDCl_3$) δ 8.89 (s, 1H, NH), 7.54-7.47 (m, 4H, ArH), 7.37-7.30 (m, 4H, ArH), 6.98 (s, 1H, CH), 4.66 (s, 1H, OH), 4.37-4.23 (m, 5H, OCH_2), 4.17-4.07 (m, 3H, OCH_2), 2.72 (d, *J* = 14.8 Hz, 1H, CH_2), 2.49 (d, *J* = 15.2 Hz, 1H, CH_2), 1.40 (t, *J* = 7.2 Hz, 3H, CH_3), 1.30 (t, *J* = 7.2 Hz, 3H, CH_3), 1.16 (t, *J* = 7.2 Hz, 3H, CH_3), 1.07 (t, *J* = 7.2 Hz, 3H, CH_3). ^{13}C NMR (100 MHz, $CDCl_3$) δ 166.0, 164.3, 163.0, 160.3, 159.3, 152.7, 149.5, 144.3, 138.8, 132.6, 131.4, 130.6, 129.0, 127.5, 127.4, 125.3, 124.8, 122.6, 117.1, 113.0, 85.9, 72.8, 64.6, 62.8, 62.3, 62.0, 52.8, 51.2, 13.9, 13.8, 13.7, 13.6. MS (*m/z*): HRMS (ESI) Calcd. for $C_{37}H_{33}Br_2NaN_3O_{10}$ ($[M+Na]^+$): 860.0430, found: 860.0411.

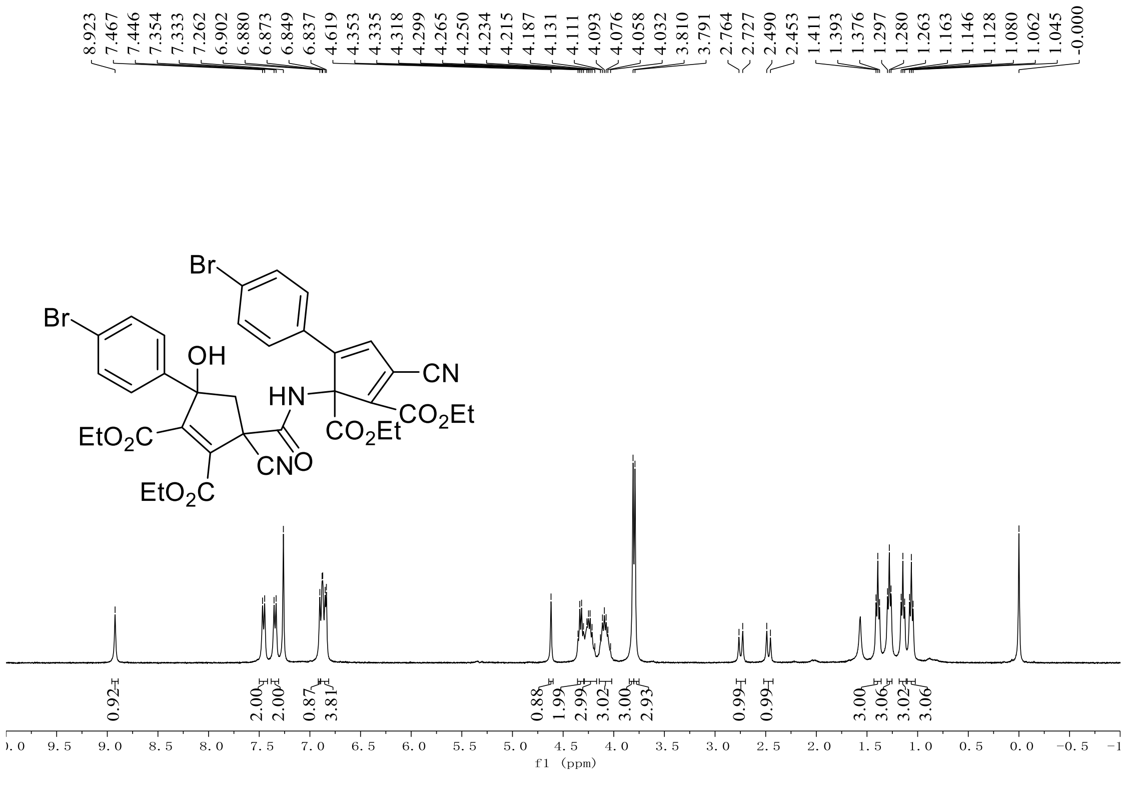

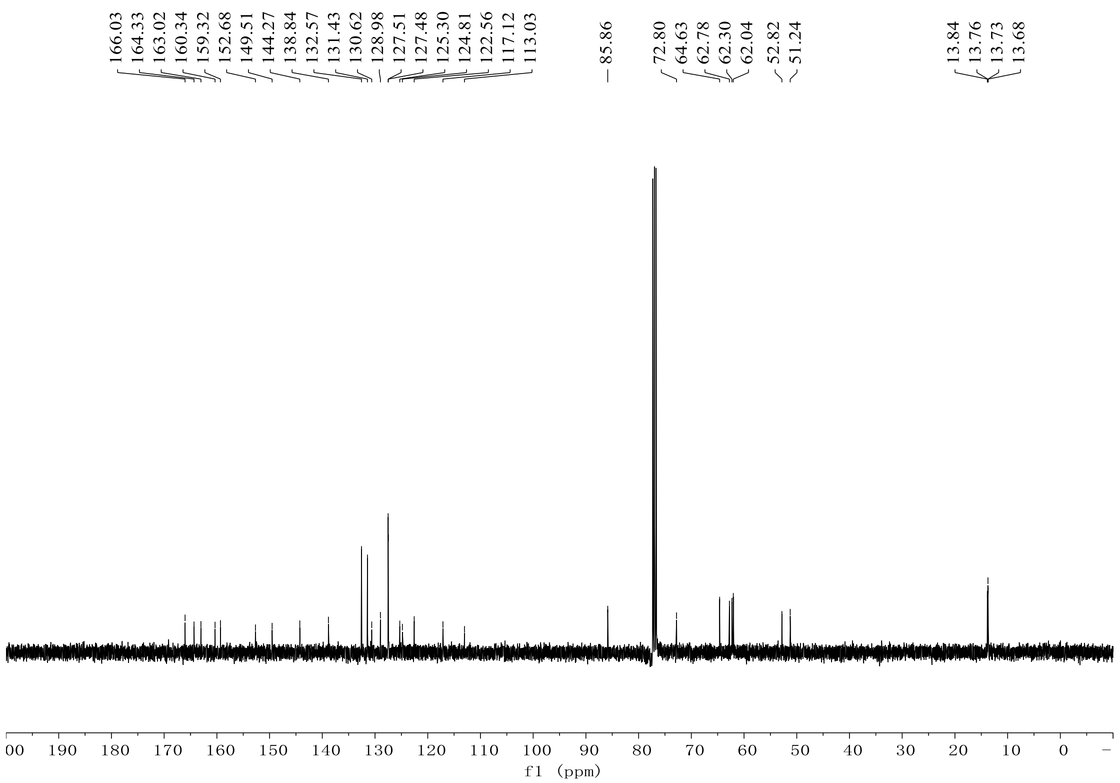

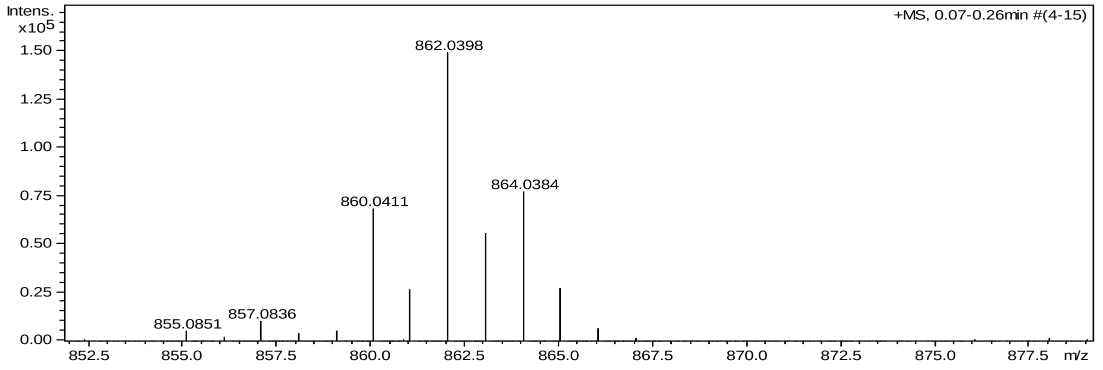

Dimethyl 3-cyano-1-(1-cyano-4-hydroxy-2,3-bis(methoxycarbonyl)-4-(4-methoxyphenyl)cyclopent-2-ene-1-carboxamido)-5-(4-methoxyphenyl)cyclopenta-2,4-diene-1,2-dicarboxylate (4h): yellow solid, 54%, m.p. 172-174 °C; ^{1}H NMR (400 MHz, $CDCl_3$) δ 8.95 (s, 1H, NH), 7.45 (d, *J* = 8.4 Hz, 2H, ArH), 7.34 (d, *J* = 8.0 Hz, 2H, ArH), 6.91 (s, 1H, CH), 6.89-6.86 (m, 4H, ArH), 4.63 (s, 1H, OH), 3.89 (s, 3H, OCH_3), 3.82 (s, 3H, OCH_3), 3.80 (s, 3H, OCH_3), 3.76 (s, 6H, OCH_3), 3.66 (s, 3H, OCH_3), 2.74 (d, *J* = 14.8 Hz, 1H, CH_2), 2.53(d, *J* = 14.0 Hz, 1H, CH_2). ^{13}C NMR (100 MHz, $CDCl_3$) δ 167.2, 164.2, 163.9, 161.3, 160.8, 160.0, 159.5, 154.0, 150.9, 142.0, 131.7, 130.3, 127.8, 126.8, 126.1, 124.9, 122.8, 117.3, 114.8, 113.7, 113.3, 86.0, 72.7, 55.4, 55.2, 54.9, 53.1, 52.8, 52.7, 52.5, 51.4. MS (*m*/*z*): HRMS (ESI) Calcd. for $C_{35}H_{31}NaN_3O_{12}$ ($[M+Na]^+$): 708.1805, found: 708.1800.

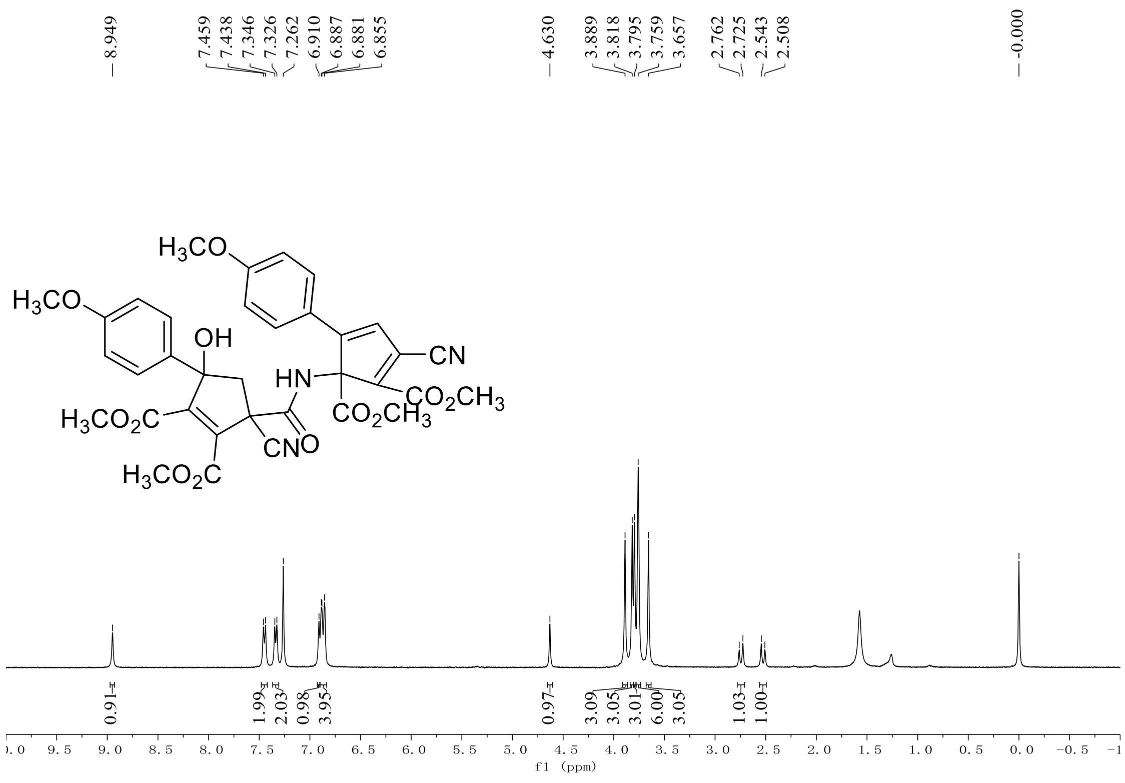
8.949
7.459
7.438
7.346
7.326
7.262
6.910
6.887
6.881
6.855
4.630
3.889
3.818
3.795
3.759
3.657
2.762
2.725
2.543
2.508
-0.000
H3CO
H3CO
OH
HN
CN
H3CO2C
CO2CH3
CO2CH3
CN
O
H3CO2C
0.91
1.99
2.03
0.98
3.95
0.97
3.09
3.05
3.01
6.00
3.05
1.03
1.00
f1 (ppm)

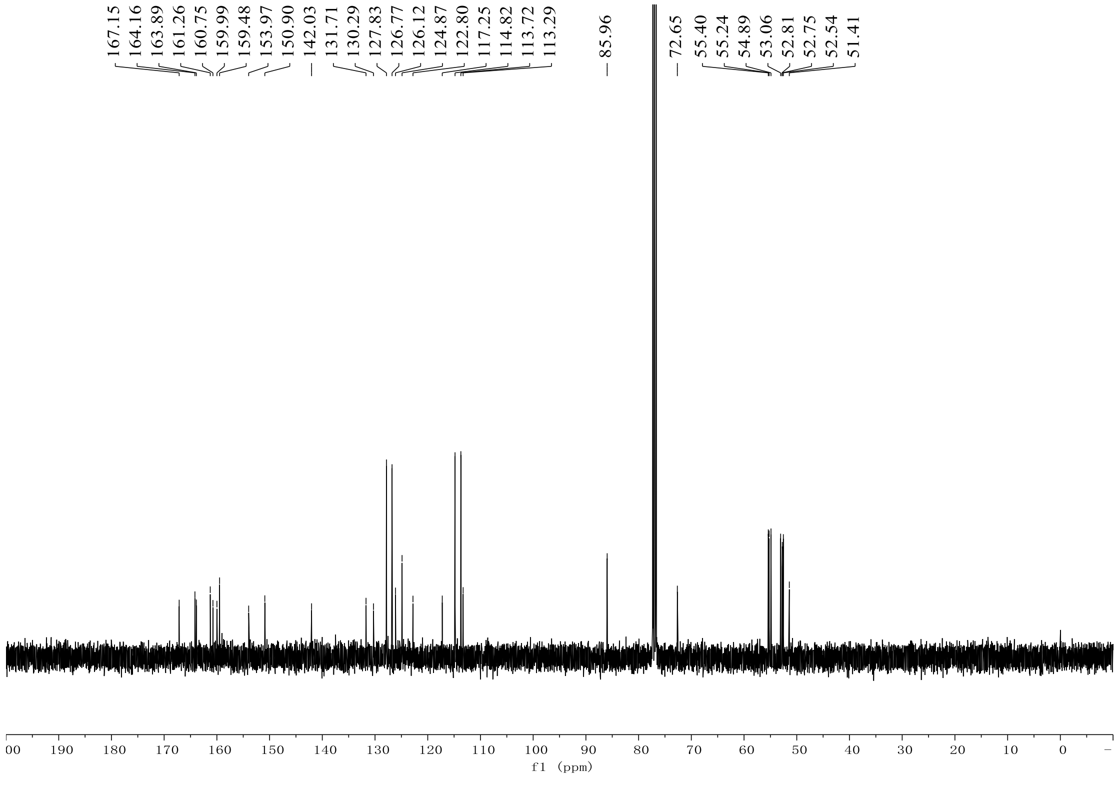
167.15
164.16
163.89
161.26
160.75
159.99
159.48
153.97
150.90
142.03
131.71
130.29
127.83
126.77
126.12
124.87
122.80
117.25
114.82
113.72
113.29
85.96
72.65
55.40
55.24
54.89
53.06
52.81
52.75
52.54
51.41
f1 (ppm)

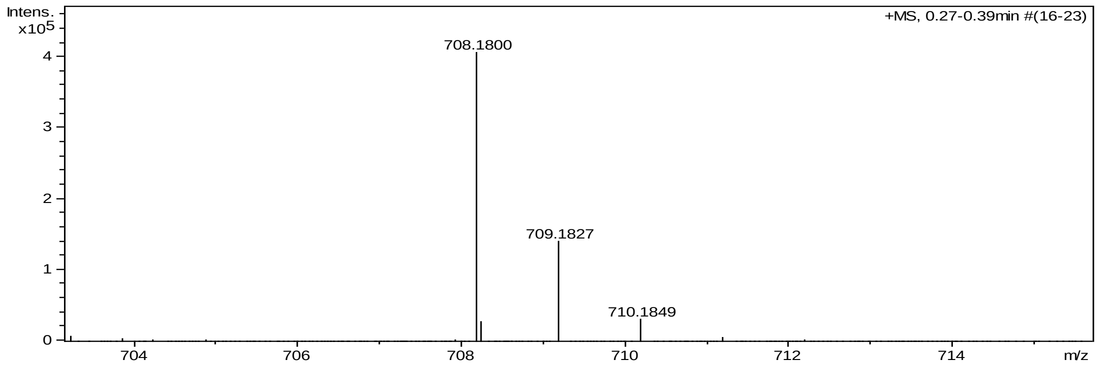

Dimethyl 3-cyano-1-(1-cyano-4-hydroxy-2,3-bis(methoxycarbonyl)-4-phenylcyclopent-2-ene-1-carboxamido)-5-phenylcyclopenta-2,4-diene-1,2-dicarboxylate (4i): fluorescent green solid, 53%, m.p. 181-183 °C; ^{1}H NMR (400 MHz, $CDCl_3$) δ 8.96 (s, 1H, NH), 7.48-7.47 (m, 2H, ArH), 7.42-7.36 (m, 7H, ArH), 7.31 (d, J = 6.8 Hz, 1H, ArH), 6.99 (s, 1H, CH), 4.63 (s, 1H, OH), 3.91 (s, 3H, OCH_3), 3.78 (s, 3H, OCH_3), 3.71 (s, 3H, OCH_3), 3.64 (s, 3H, OCH_3), 2.76 (d, J = 14.8 Hz, 1H, CH_2), 2.49(d, J = 15.2 Hz, 1H, CH_2). ^{13}C NMR (100 MHz, $CDCl_3$) δ 166.9, 164.2, 163.8, 160.7, 159.9, 153.6, 151.1, 143.5, 139.6, 130.9, 130.4, 130.0, 129.4, 128.5, 127.1, 126.2, 125.8, 125.4, 117.1, 113.2, 86.3, 72.8, 55.0, 53.1, 53.0, 52.8, 52.7, 51.3. MS (m/z): HRMS (ESI) Calcd. for $C_{33}H_{27}NaN_3O_{10}$([M+Na]$^+$): 648.1594, found: 648.1593.

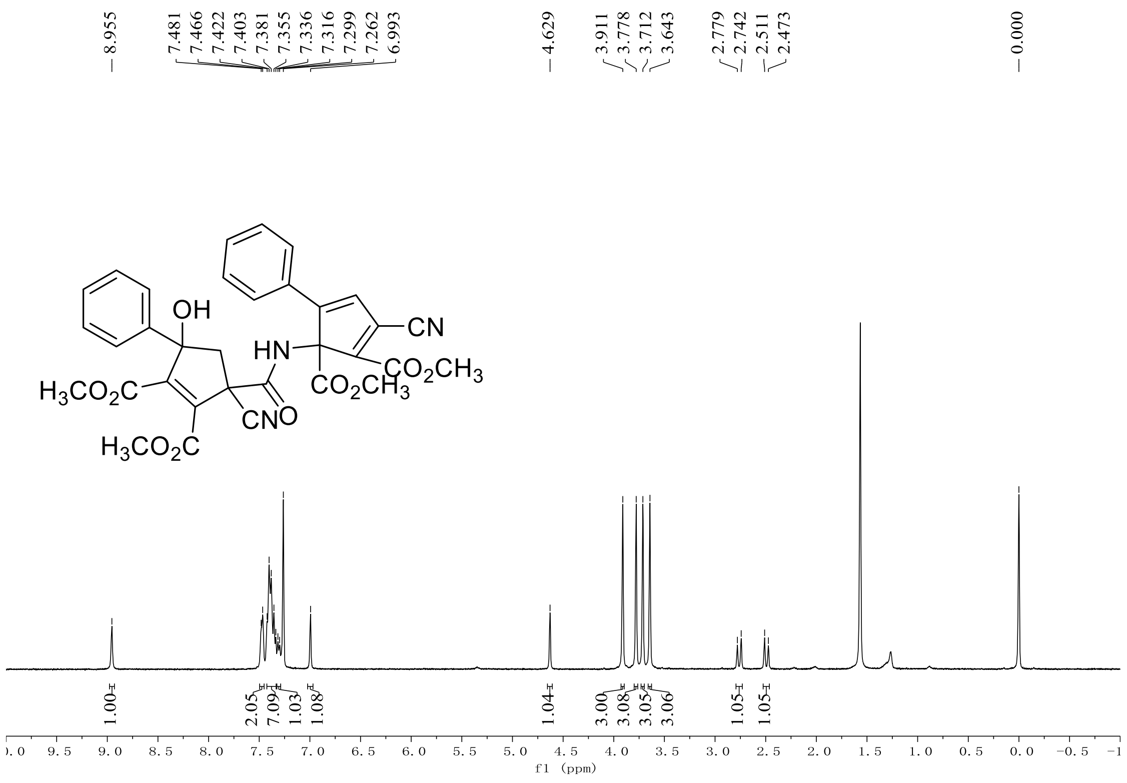

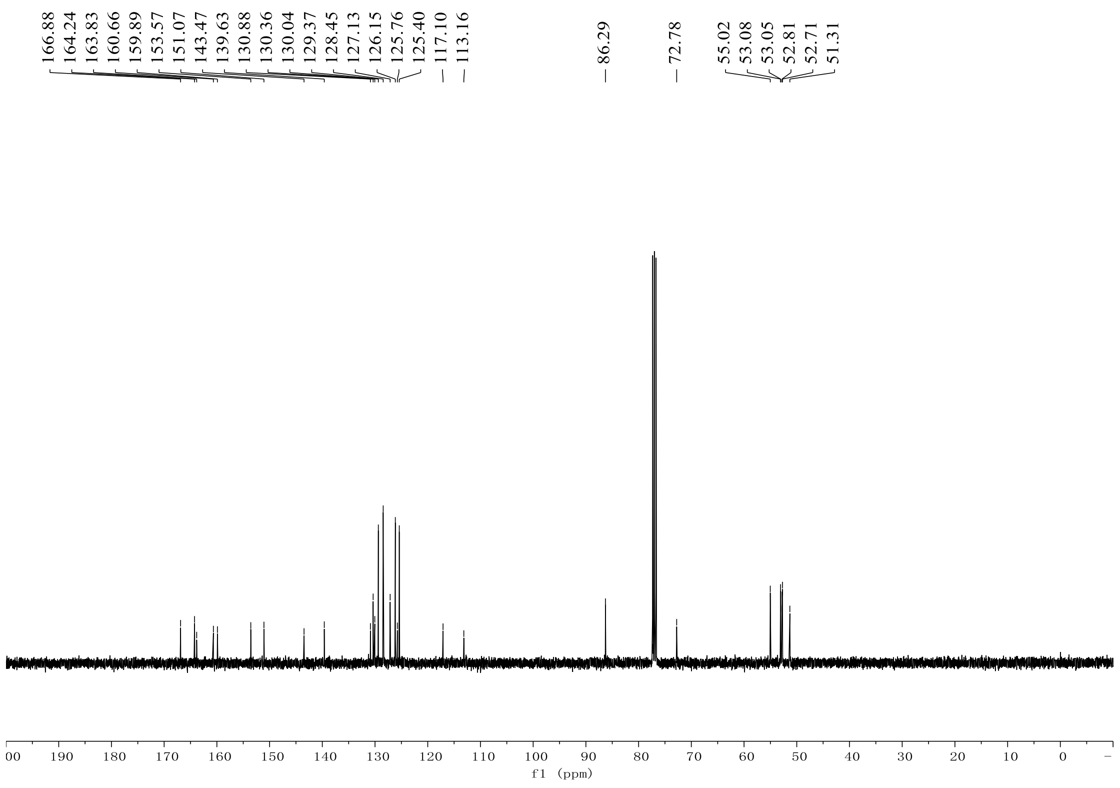

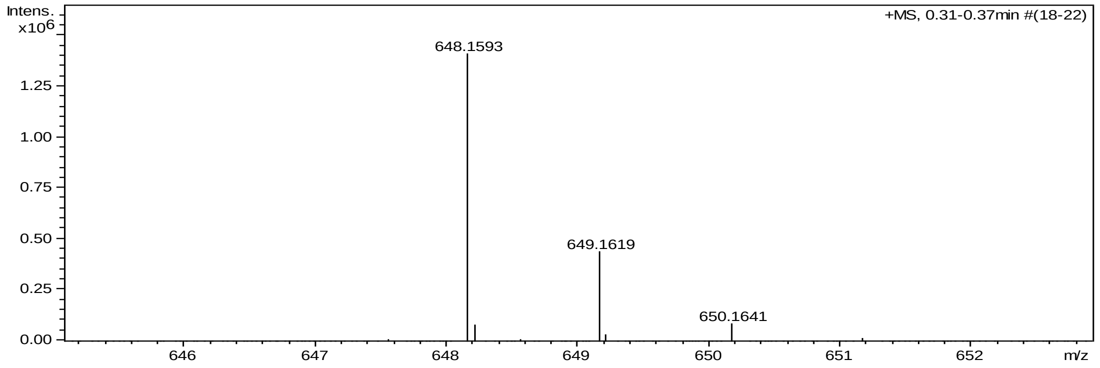

Dimethyl 5-(4-chlorophenyl)-1-(4-(4-chlorophenyl)-1-cyano-4-hydroxy-2,3-bis(methoxycarbonyl)cyclopent-2-ene-1-carboxamido)-3-cyanocyclopenta-2,4-diene-1,2-dicarboxylate (4j): pale green solid, 38%, m.p. 192-194 °C; ^{1}H NMR (400 MHz, $CDCl_3$) δ 8.91 (s, 1H, NH), 7.43-7.32 (m, 8H, ArH), 6.98 (s, 1H, CH), 4.67 (s, 1H, OH), 3.90 (s, 3H, OCH_3), 3.80 (s, 3H, OCH_3), 3.78 (s, 3H, OCH_3), 3.66 (s, 3H, OCH_3), 2.71 (d, J = 14.4 Hz, 1H, CH_2), 2.52(d, J = 14.4 Hz, 1H, CH_2). ^{13}C NMR (100 MHz, $CDCl_3$) δ 166.7, 164.3, 163.5, 160.7, 159.8, 153.1, 149.5, 143.7, 138.2, 136.6, 134.5, 130.9, 129.7, 128.6, 128.4, 127.5, 127.3, 127.0, 125.6, 116.9, 112.9, 85.8, 72.8, 55.1, 53.2, 52.9, 52.8, 52.7, 51.3. MS (*m/z*): HRMS (ESI) Calcd. for $C_{33}H_{25}Cl_2NaN_3O_{10}$ ($[M+Na]^+$): 716.0815, found: 716.0805.

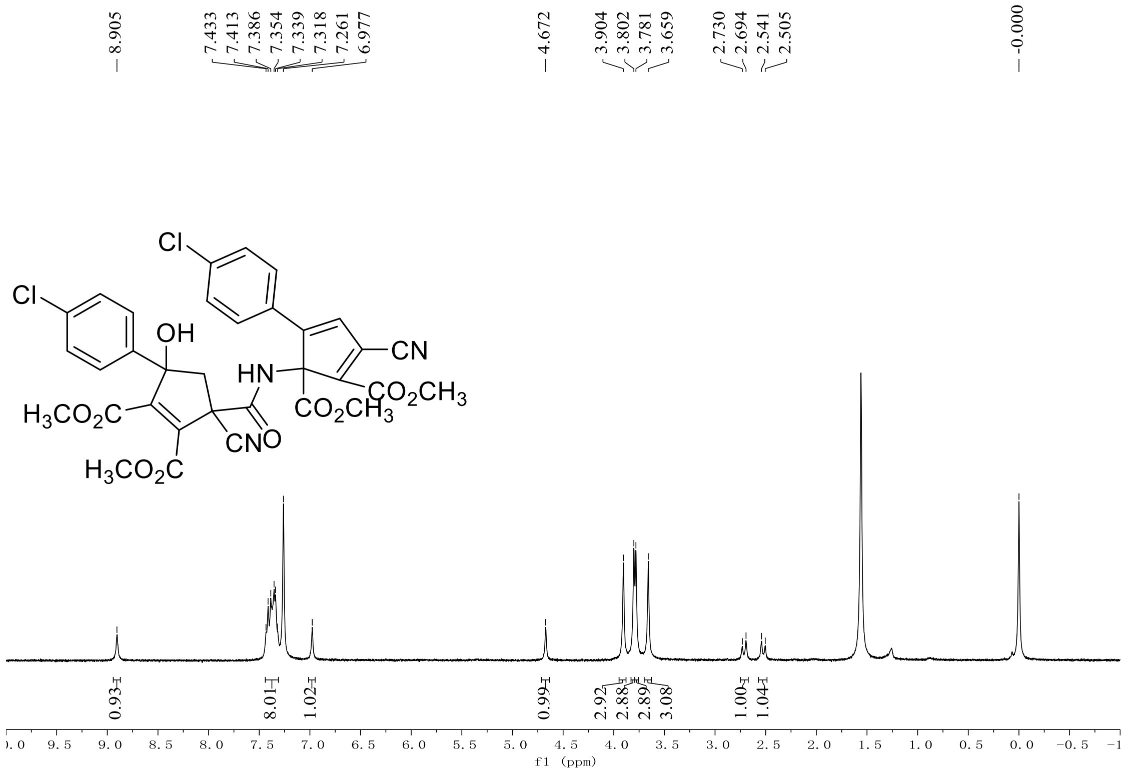
8.905
7.433
7.413
7.386
7.354
7.339
7.318
7.261
6.977
4.672
3.904
3.802
3.781
3.659
2.730
2.694
2.541
2.505
-0.000
Cl
Cl
OH
HN
CN
CO_2CH_3
CO_2CH_3
H_3CO_2C
H_3CO_2C
CN
O
0.93
8.01
1.02
0.99
2.92
2.88
2.89
3.08
1.00
1.04
f1 (ppm)

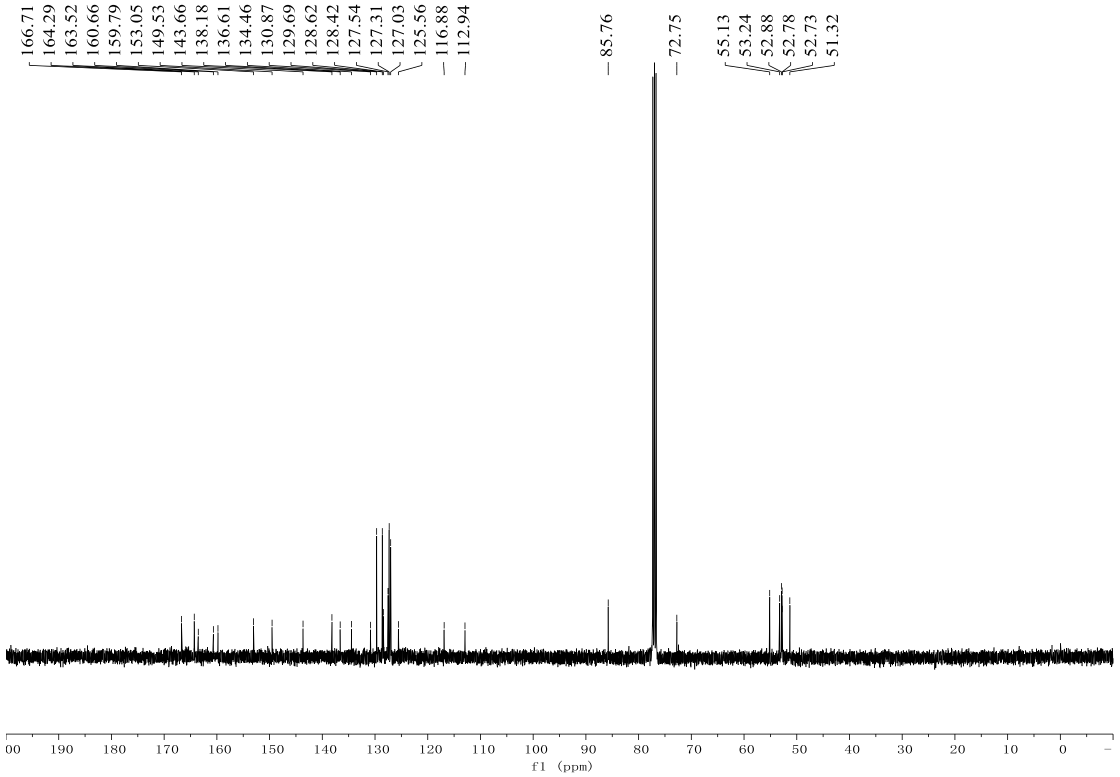
166.71
164.29
163.52
160.66
159.79
153.05
149.53
143.66
138.18
136.61
134.46
130.87
129.69
128.62
128.42
127.54
127.31
127.03
125.56
116.88
112.94
85.76
72.75
55.13
53.24
52.88
52.78
52.73
51.32
f1 (ppm)

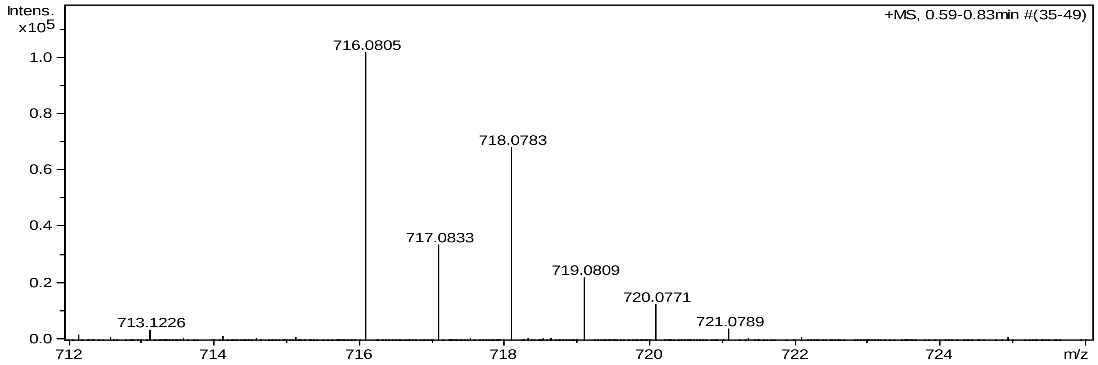

Dimethyl 5-(4-bromophenyl)-1-(4-(4-bromophenyl)-1-cyano-4-hydroxy-2,3-bis(methoxycarbonyl)cyclopent-2-ene-1-carboxamido)-3-cyanocyclopenta-2,4-diene-1,2-dicarboxylate (4k): pale green solid, 42%, m.p. 188-190 °C; ^{1}H NMR (400 MHz, $CDCl_3$) δ 8.91 (s, 1H, NH), 7.55-7.48 (m, 4H, ArH), 7.36-7.29 (m, 4H, ArH), 7.00 (s, 1H, CH), 4.66 (s, 1H, OH), 3.90 (s, 3H, OCH_3), 3.80 (s, 3H, OCH_3), 3.78 (s, 3H, OCH_3), 3.67 (s, 3H, OCH_3), 2.71 (d, *J* = 15.2 Hz, 1H, CH_2), 2.53(d, *J* = 15.2 Hz, 1H, CH_2). ^{13}C NMR (100 MHz, $CDCl_3$) δ 166.7, 164.3, 163.5, 160.7, 159.8, 152.9, 149.6, 143.7, 138.7, 132.7, 131.6, 131.0, 128.8, 127.6, 127.5, 127.3, 125.6, 125.0, 122.7, 116.9, 112.9, 85.8, 72.7, 55.1, 53.3, 52.9, 52.8, 52.7, 51.3. MS (*m*/*z*): HRMS (ESI) Calcd. for $C_{33}H_{25}Br_2NaN_3O_{10}$ ($[M+Na]^+$): 803.9804, found: 803.9788.

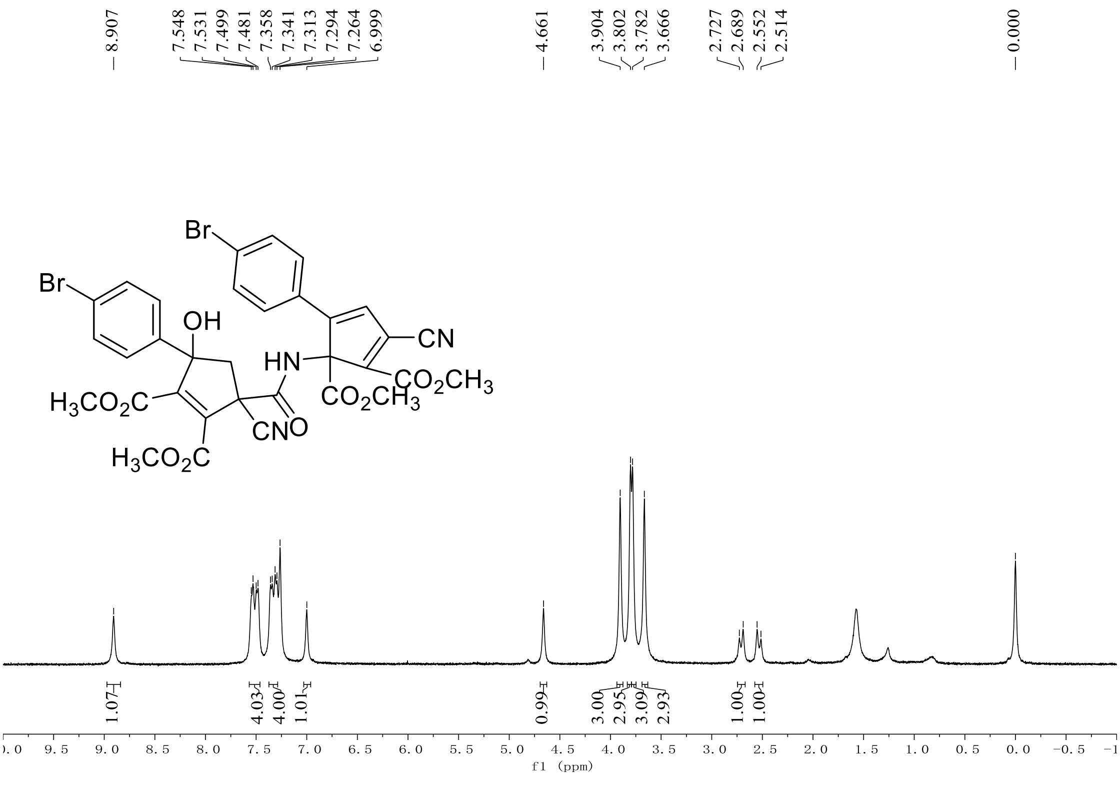

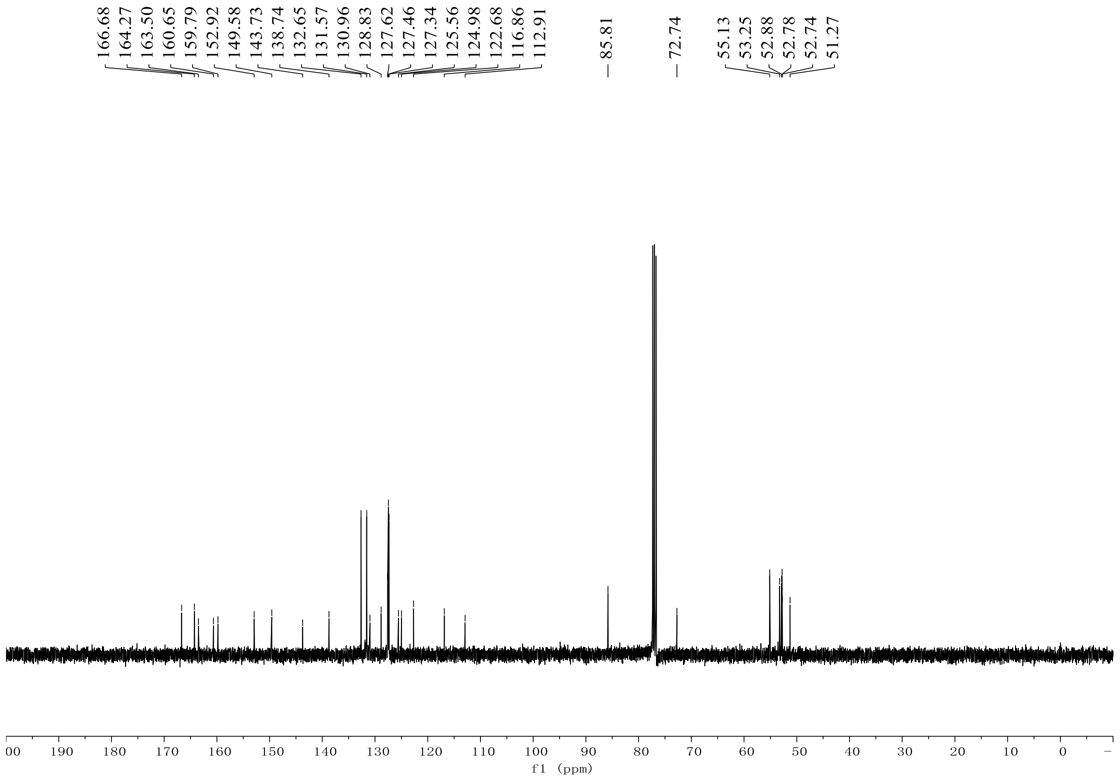

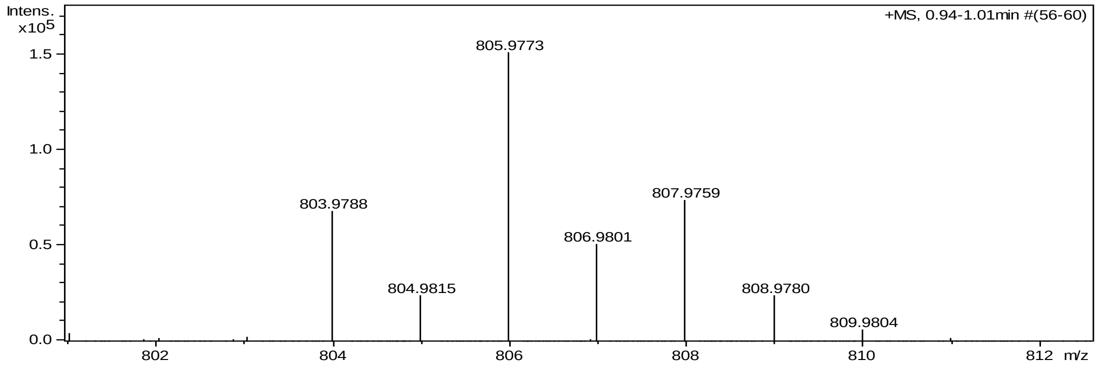

Diethyl 2-(1,1-dicyano-3-(4-methoxyphenyl)-3-oxopropyl)maleate (5): white solid, 12%. m.p. 58-60 °C; ^{1}H NMR (400 MHz, $CDCl_3$) δ 7.91 (d, *J* = 6.8 Hz, 2H, ArH), 6.97 (d, *J* = 6.8 Hz, 2H, ArH), 6.84 (s, 1H, CH), 4.36-4.27 (m, 4H, OCH_2), 4.03 (s, 2H, CH_2), 3.89 (s, 3H, OCH_3), 1.33 (s, 6H, CH_3). ^{13}C NMR (100 MHz, $CDCl_3$) δ 189.4, 164.7, 163.2, 135.3, 130.6, 129.3, 127.4, 114.2, 112.3, 63.3, 62.1, 55.6, 45.4, 36.6, 14.0, 13.7. MS (*m*/*z*): HRMS (ESI) Calcd. for $C_{20}H_{20}NaN_2O_6$ ($[M+Na]^+$): 407.1219, found: 407.1216.

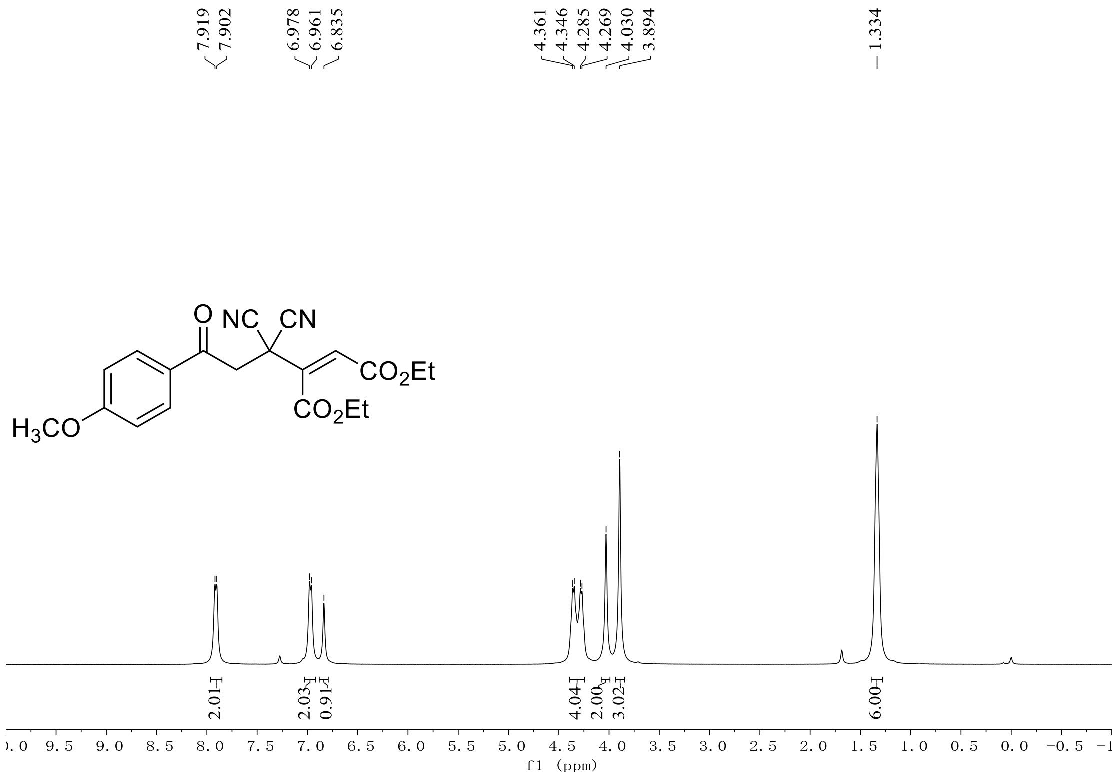
7.919
7.902
6.978
6.961
6.835
4.361
4.346
4.285
4.269
4.030
3.894
1.334
O
NC
CN
CO_2Et
CO_2Et
H_3CO
2.01
2.03
0.91
4.04
2.00
3.02
6.00
f1 (ppm)

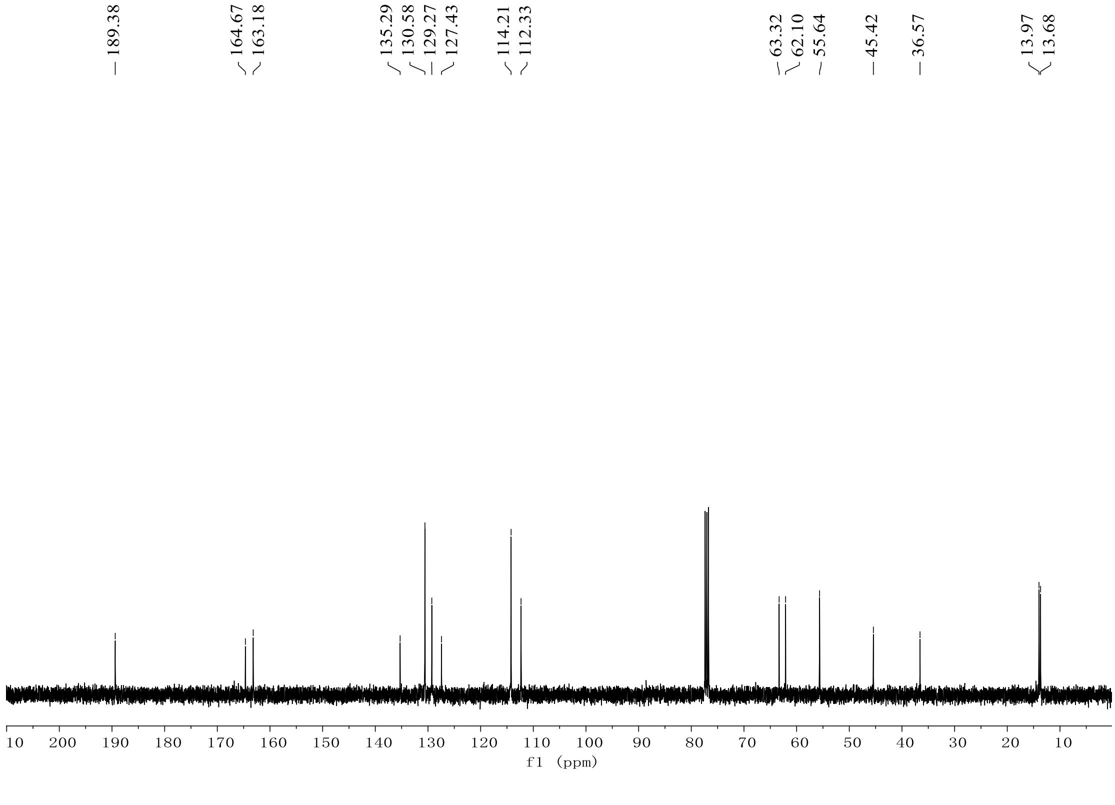
189.38
164.67
163.18
135.29
130.58
129.27
127.43
114.21
112.33
63.32
62.10
55.64
45.42
36.57
13.97
13.68
f1 (ppm)

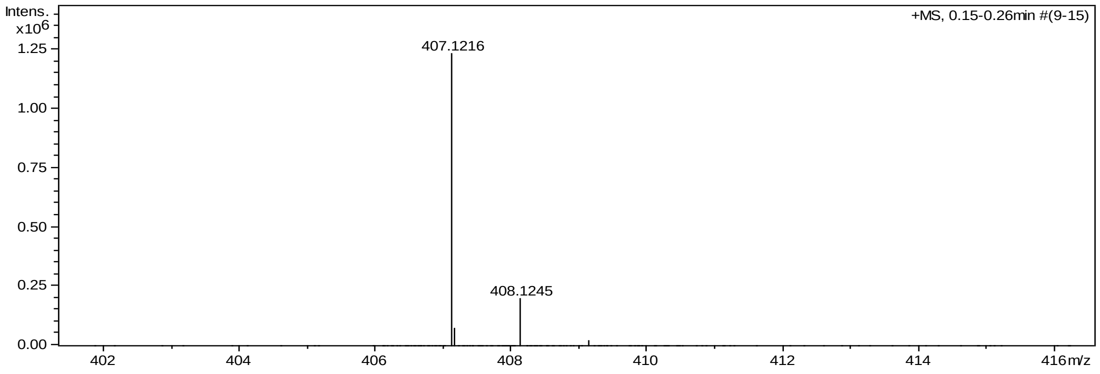
Intens.
x10^6
1.25
1.00
0.75
0.50
0.25
0.00
+MS, 0.15-0.26min #(9-15)
407.1216
408.1245
402
404
406
408
410
412
414
416
m/z
